# Ligand-directed oral lipidic nanoplatform enables sustained ferroptosis and immune reprogramming via multivalent transporter-mediated metronomic delivery

**DOI:** 10.7150/thno.124189

**Published:** 2026-01-01

**Authors:** Laxman Subedi, In Ho Im, Arjun Dhwoj Bamjan, Jiwon Jeon, Susmita Phuyal, Yun-Hwa Jeong, Seung Hyun Kim, Jung-Hyun Shim, Jeong Uk Choi, Jin Woo Park

**Affiliations:** 1 Department of Biomedicine, Health & Life Convergence Sciences, BK21 Four, Biomedical and Healthcare Research Institute, Mokpo National University, Jeonnam 58554, Republic of Korea.; 2 Biomedicine Cutting Edge Formulation Technology Center, Mokpo National University, Jeonnam 58554, Republic of Korea.; 3 Department of Regulatory Science, Graduate School, Kyung Hee University, Seoul 02447, Republic of Korea.; 4 College of Pharmacy, Kyung Hee University, Seoul 02447, Republic of Korea.; 5 College of Pharmacy and Natural Medicine Research Institute, Mokpo National University, Jeonnam 58554, Republic of Korea.

**Keywords:** combination chemotherapy, metronomic low-dose regimen, oral nanoplatform, multimodal transporter-facilitated absorption, ferroptosis, immune modulation

## Abstract

**Rationale:** Ferroptosis-induced tumor cell death and immune activation represent promising strategies for overcoming therapeutic resistance in triple-negative breast cancer (TNBC). However, clinical application remains limited by poor oral absorption, transient immune activation, and systemic toxicity.

**Methods:** We developed an orally administrable nanoplatform (MCT-NE#9) co-delivering docetaxel (DTX) and atorvastatin (ATV), designed to enhance intestinal uptake via bile acid and vitamin transporters. Pharmacokinetic, *in vitro*, and *in vivo* studies were conducted to evaluate drug absorption, sustained ferroptosis, and immune modulation.

**Results:** MCT-NE#9 markedly improved oral bioavailability (659% for ATV, 851% for DTX) and sustained intratumoral drug levels under a low-dose metronomic regimen. Mechanistically, it induced sustained ferroptosis by promoting iron accumulation, lipid peroxidation, and GPX4 suppression, while remodeling the tumor immune microenvironment. Treatment increased M1 macrophages and antigen-presenting cells and reduced TGFβ1, regulatory T cells, and M2 macrophages. *In vivo*, oral MCT-NE#9 suppressed tumor growth by 50.4%, with enhanced efficacy (70.3% inhibition) when combined with anti-CD47 therapy.

**Conclusion:** MCT-NE#9 enables a synergistic, low-toxicity chemo-immunotherapeutic strategy by sustaining ferroptosis and reprogramming the immune microenvironment via transporter-targeted oral delivery. This ligand-directed nanoplatform offers a clinically translatable approach for effective TNBC treatment.

## Introduction

A major challenge in breast cancer treatment is therapeutic resistance 1. Various approaches, including radiation, chemotherapy, and targeted therapy, are used, based on cancer stage and subtype; however, their effectiveness is inherently limited. Triple-negative breast cancer (TNBC) is particularly challenging because of its lack of estrogen receptor, progesterone receptor, and human epidermal growth factor receptor 2 (HER2), making it unresponsive to hormonal and HER2-targeted therapies. While immunotherapy shows promise, its efficacy varies among patients 2-5. A deeper understanding of the tumor microenvironment (TME) and immune modulation is crucial for improving treatment outcomes, highlighting the need for novel therapeutic strategies for TNBC 6.

Docetaxel (DTX) is a first-generation chemotherapeutic that is widely used for breast cancer treatment, inducing apoptosis through microtubule disruption 7. However, its high-dose intravenous (IV) administration causes significant toxicity, and prolonged exposure leads to resistance, limiting its clinical efficacy 8. Combining DTX with other agents to reduce its effective dose may help to overcome resistance and enhance its anticancer activity 9. Atorvastatin (ATV), a lipid-lowering agent, inhibits HMG-CoA reductase in the mevalonate pathway, suppressing cholesterol biosynthesis and compromising cell viability 10. Additionally, by reducing farnesyl pyrophosphate levels, it disrupts the Ras-Raf signaling cascade that supports cell proliferation and survival 11,12. These mechanisms highlight ATV's potential anticancer properties by inhibiting cancer cell growth and survival pathways 13.

Ferroptosis is defined as iron-dependent regulated cell death driven by lipid peroxidation (LPO) due to iron accumulation and reactive oxygen species production 14. Inducing ferroptosis in cancer cells can reshape the TME by releasing damage-associated molecular patterns (DAMPs) such as high-mobility group box 1 protein (HMGB1) and adenosine triphosphate, activating immune cells 15,16. Additionally, lipid peroxides generated during ferroptosis are recognized by Toll-like receptor 2 (TLR2) on macrophages, triggering phagocytosis and tumor antigen presentation 17. These mechanisms highlight the induction of ferroptosis as a promising strategy for breast cancer therapy. Recent evidence indicates that both DTX and ATV can induce ferroptosis 18,19; DTX inhibits the System Xc-/glutathione peroxidase 4 (GPX4) 20, a key regulator of ferroptosis, whereas ATV enhances ferroptosis by inhibiting both the System Xc-/GPX4 and mevalonate pathways, leading to GPX4 downregulation 21,22. Because high-dose DTX causes significant adverse effects, combining it with ATV at a reduced dose may provide an effective strategy for inducing ferroptosis in breast cancer cells 23,24. Recent studies have shown that ferroptosis progresses through distinct stages, with only the terminal stage characterized by robust DAMP release. These DAMPs include HMGB1 and adenosine triphosphate, which are crucial for effective dendritic cell maturation and immune activation. In particular, Wiernicki *et al.* demonstrated that early-stage ferroptotic cells fail to promote dendritic cell maturation and antigen cross-presentation due to insufficient DAMP signaling. By contrast, sustained ferroptosis—extending beyond the early phase—may enhance immunogenicity and reprogram the TME, thereby improving therapeutic efficacy 25.

To optimize the therapeutic effects of combining DTX and ATV and achieve sustained tumor cell ferroptosis, precise dose modulation and scheduling are crucial 26-28. Low-dose metronomic chemotherapy (MCT), which involves frequent low-dose administration without drug-free intervals, maintains effective drug levels, reduces toxicity, and may promote sustained tumor cell ferroptosis 29. Additionally, it persistently recruits immune cells, such as natural killer (NK) cells, dendritic cells, and macrophages, preventing tumor recurrence and metastasis 30-32. However, poor oral bioavailability due to low solubility and first-pass metabolism limits its effectiveness 33,34. To overcome this, surface-modified nanocarriers targeting intestinal transporters, combined with P-glycoprotein (P-gp) efflux inhibitors such as D-α-tocopheryl polyethylene glycol 1000 succinate (TPGS), have been developed. Our previous studies showed that this approach increased DTX and ATV bioavailability by 249% and 1,091%, respectively, compared with free drugs 35,36.

Combining MCT with immunotherapeutic agents can amplify antitumor immune activation, as chemotherapy by itself rarely elicits a sufficiently robust immune response, while immunotherapy alone often shows limited efficacy 30. Growing interest has focused on pairing MCT with immune checkpoint blockade, as metronomic dosing promotes initial immune cell activation, whereas checkpoint inhibitors help maintain and prolong this antitumor immune response 37. Blocking CD47, a “do not eat me” signal, in ferroptotic cancer cells can enhance macrophage activation 38-40. Moreover, combining ferroptosis inducers with anti-CD47 therapy may help reprogram the immune microenvironment, offering a promising multimodal anticancer strategy 41.

**Scheme 1:**
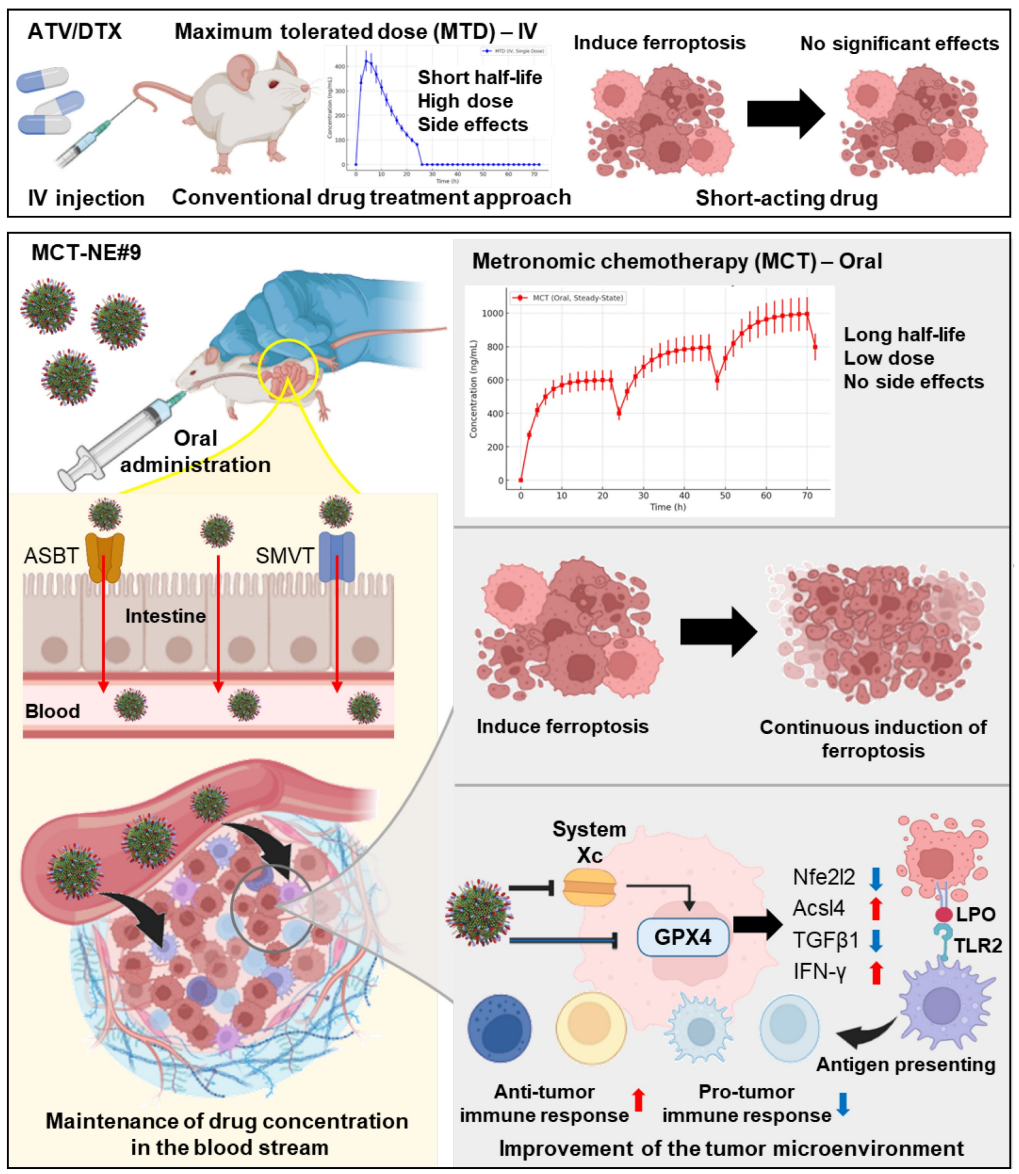
Schematic overview of strategy for sustained ferroptosis-driven immunotherapy using MCT-NE. ASBT: Apical sodium-dependent bile acid transporter; SMVT: Sodium-dependent multivitamin transporter; GPX4: Glutathione peroxidase 4; LPO: Lipid peroxidation.

As this study aimed to optimize sustained ferroptosis activation and immunomodulation using a low-dose metronomic regimen of ATV and DTX, the addition of an anti-CD47 antibody was expected to produce synergistic effects (Scheme 1). To enhance oral bioavailability and efficacy, TPGS was incorporated to inhibit P-gp activity, and the formulation was designed to engage apical sodium-dependent bile acid transporter (ASBT) as well as sodium-dependent multivitamin transporter (SMVT) to facilitate active intestinal uptake. The ATV/DTX-loaded nanoplatform (MCT-NE) was generated using a combined surfactant system designed to increase the solubility and a functional ligand complex for improved absorption. Permeability and transport mechanisms were evaluated in Caco-2/HT29-MTX-E12 monolayers. *In vitro* studies were performed to assess ferroptosis markers, while *in vivo* experiments were performed to confirm pharmacokinetics, tumor inhibition, and immunomodulatory effects.

## Materials and Methods

### Materials

ATV and simvastatin, employed as the internal standard (IS) for ATV quantification, were sourced from Shangyu Jingxin Pharmaceutical Co., Ltd. (Weisan, China). DTX, paclitaxel (PTX; IS of DTX), sodium deoxycholate (DOCA), TPGS, polyoxyethylene sorbitan monooleate 80 (Tween 80), actinomycin D (Act D), clofazimine (CFZ), pantothenic acid (PA), chlorpromazine, genistein, methyl-β-cyclodextrin (MBCD), brefeldin A, cyclosporine A (Cys A), RAS-selective lethal 3 (RSL3), and ferrostatin-1 (Fer-1) were purchased from Sigma-Aldrich (St. Louis, MO, USA). Propylene glycol monocaprylate type II (Capryol 90) and diethylene glycol monoethyl ether (Transcutol HP) were obtained by Gattefossé (Saint-Priest, France). Staurosporine (STS) and CU-CPT22 were purchased from MedChemExpress (Monmouth Junction, NJ, USA). Biotinylated phosphatidylethanolamine (Biotinyl PE) and the cationic lipid 1,2-dioleoyl-3-trimethylammonium-propane (DOTAP) were purchased from Avanti Polar Lipids (Alabaster, AL, USA). Reagents required for chromatographic analyses, including those for high-performance liquid chromatography (HPLC) and ultra-performance liquid chromatography-tandem mass spectrometry (UPLC-MS/MS) assays, were purchased from Thermo Fisher Scientific (Waltham, MA, USA) and Alfa Aesar (Ward Hill, MA, USA). All materials employed in the experiments met analytical-grade specifications. The antibodies utilized for flow cytometry and western blot analyses are summarized in Table S1.

### Animals

Sprague-Dawley rats (males, 200-250 g) and BALB/c mice (females, 20-25 g) were obtained from G-Bio (Gwangju, Republic of Korea). C57BL/6 mice were obtained from Orient Bio (Seongnam, Republic of Korea). The animals were housed under standard laboratory conditions, maintained at a temperature of 23 ± 2 °C, with a relative humidity (RH) of 55 ± 10%, and a 12-h light/dark cycle. The animals were maintained on a conventional laboratory chow (Nestlé Purina, St. Louis, MO, USA) and were allowed free access to ion-sterilized drinking water. All experimental procedures involving animals were reviewed and approved by the Institutional Animal Care and Use Committee (IACUC) of Mokpo National University (Jeonnam, Republic of Korea) (approval nos. MNU-IACUC-2023-002 and MNU-IACUC-2023-009) and Kyung Hee University (Seoul, Republic of Korea) (approval no. KHSASP-24-239). All experimental activities were performed in compliance with the guidelines outlined by the National Institutes of Health for the care and ethical use of laboratory animals, as well as IACUC guidelines.

### Preparation of MCT-NE formulations

To promote intestinal entry of the nanoemulsions (NEs) co-encapsulating ATV and DTX via bile acid- and/or multivitamin-transporter-assisted uptake, DOCA was combined with the cationic lipid DOTAP at an equimolar ratio to generate an ionically associated DOCA-TAP complex 42. In brief, an aqueous solution of DOCA (3.33 mg/mL) was added dropwise to DOTAP in ethanol (28.1 mg/mL) with continuous stirring. Separately, an aqueous solution of L-lysine-linked deoxycholic acid (DCK, 3.33 mg/mL) was added dropwise to Biotinyl PE in ethanol (28.1 mg/mL) with continuous stirring, forming an ionic complex of DCK/Biotinyl PE at a 1:1 molar ratio to facilitate dual transporter (ASBT and SMVT)-mediated cellular uptake of the NE. The two mixtures were then completely dried using a rotary evaporator at 50 °C and 75 °C, respectively, for 3 h. The resulting dried mixtures were re-suspended in 30 mL of water and then subjected to freeze-drying at -70 °C, yielding the corresponding formulations as dry powders.

To determine whether DCK formed an ionic complex with Biotinyl PE, the samples were analyzed by ultrafiltration before and after freeze-drying. For this assessment, each preparation was first diluted with water and loaded into a centrifugal ultrafiltration unit equipped with a 1,000 Da molecular weight cut-off (MWCO) membrane (Thermo Fisher Scientific). The tubes were centrifuged at 12,000 × *g* for 30 minutes, after which the permeate was collected. Free, non-associated DCK in the filtrate was quantified via HPLC using a C18 analytical column (4.6 × 150 mm, 5 µm, 100 Å) set to 35 °C. Chromatographic separation was achieved with a mobile phase composed of acetonitrile, 20 mM sodium acetate buffer (pH 4.3), and methanol at a ratio of 60:35:5 (v/v/v), delivered at a flow rate of 1.5 mL/min, and monitored at 210 nm. In all samples, the filtrates contained no detectable free DCK, indicating that DCK was fully ionically paired with Biotinyl PE in the DCK/Biotinyl PE complex.

Differential scanning calorimetry (DSC) was employed to characterize the thermal properties of the DCK/Biotinyl PE complex. Individual components (DCK and Biotinyl PE), their physical mixture, and the prepared complex were subjected to DSC analysis using a TA Instruments system equipped with Universal Analysis software (V4.5A; New Castle, DE, USA). In addition, Fourier transform infrared (FT-IR) spectroscopy was carried out to evaluate ionic interactions within the complex. FT-IR spectra were recorded on a Frontier system (PerkinElmer, Wellesley, MA, USA) equipped with an attenuated total reflectance (ATR) module.

To determine the optimal weight ratio of ATV to DTX in NEs that would yield the maximum anti-cancer effects, a preliminary cytotoxicity study was conducted. Briefly, 1 × 10^4^ 4T1 cells were seeded in each well of 96-well plates in 100 µL of Dulbecco's modified Eagle's medium (DMEM) supplemented with 10% (v/v) fetal bovine serum (FBS) and 1% (v/v) penicillin/streptomycin, then cultured at 37 °C for 48 h. The cells were subsequently treated with drug solutions at concentrations of 50, 10, or 100 µg/mL in 0.5% DMSO, containing ATV and DTX at weight ratios of 1:0, 0:1, 0.25:1, 0.5:1, 1:0.25, 1:0.5, and 1:1 for each concentration. Following an additional 24 h incubation period, cell viability was quantified using the CCK-8 assay. A 10-µL aliquot of the CCK-8 reagent (2-(2-methoxy-4-nitrophenyl)-3-(4-nitrophenyl)-5-(2,4-disulfophenyl)-2H-tetrazolium monosodium salt; Dojindo Molecular Technologies, Rockville, MD, USA) was dispensed into each well, and plates were incubated for 30 min. Absorbance at 450 nm was subsequently recorded with a multimode microplate reader (PerkinElmer, Wellesley, MA, USA). Cell viability was expressed relative to the untreated control group. Based on the synergistic cytotoxic effects of ATV and DTX, the optimal weight ratio of ATV to DTX was identified for further NE formulation.

A mixed NE formulation was generated to enhance the solubility and intestinal uptake of ATV and DTX. For this purpose, ATV (1 mg) and DTX (4 mg), at a weight ratio of 0.25:1, were dispersed in 16.8 µL of Capryol 90, which served as the oil phase of the system. The drug-oil mixture was then combined with a surfactant/co-surfactant blend composed of Tween 80 and Transcutol HP (S_mix_; 1:2, w/w). Transporter-targeting excipients—including TPGS, DCK, DOTAP, DOCA, Biotinyl PE, DOCA-TAP, and/or the DCK/Biotinyl PE complex—were subsequently added to promote active, transporter-assisted cellular internalization. Upon introduction into the external aqueous phase, the formulation spontaneously generated a stable o/w NE, as summarized in Table S2.

### Characterization of MCT-NEs

The physicochemical properties of the MCT-NE aqueous dispersions—including droplet diameter, polydispersity index (PDI), and zeta potential—were evaluated using a dynamic light-scattering instrument (Zetasizer Nano ZS90, Malvern Instruments, Malvern, UK). Morphological evaluation of the NEs was conducted through transmission electron microscope (TEM) (JEM-200; JEOL Ltd., Tokyo, Japan) after negative staining with an aqueous solution of phosphotungstic acid (2%, w/v). Encapsulation efficiencies for ATV and DTX were determined following an additional dilution step in water. A 0.5-mL aliquot of the MCT-NE formulation—containing DTX at 100 µg/mL—was mixed with water, after which an equal volume (0.5 mL) was loaded into the reservoir of an ultrafiltration spin device (Pierce Protein Concentrator, MWCO 50,000; Thermo Fisher Scientific). The diluted samples were centrifuged at 15,000 × *g* for 30 min at 4 °C to remove any non-encapsulated ATV or DTX. The amount of non-encapsulated drug present in the filtrate was quantified using HPLC. ATV was quantified using a C18 analytical column (4.6 × 250 mm, 5 µm, 100 Å; 30 µL injection volume). Chromatographic separation was carried out with a mobile phase composed of 0.05 M dipotassium hydrogen phosphate buffer (pH adjusted to 3.0 with phosphoric acid) and acetonitrile at a 35:65 (v/v) ratio, operated at a flow rate of 1 mL/min and monitored at 246 nm. For DTX, chromatographic separation was performed on a C18 column (4.6 × 250 mm, 5 µm, 100 Å; injection volume 50 µL) maintained at 30 °C. Water and acetonitrile (55:45, v/v) were used as the mobile phase at a flow rate of 1.2 mL/min, and detection was carried out at 230 nm. Encapsulation efficiency (%) was computed using the formula: encapsulation efficiency (%) = (C_i_ - C_f_)/C_i_ × 100, where C_i_ (µg/mL) represents the initial concentration of ATV or DTX added, and C_f_ (µg/mL) is the concentration of ATV or DTX in the filtrate.

### *In vitro* artificial intestinal membrane permeability

The effective *in vitro* permeability (*P_e_*) of ATV and DTX, either dispersed in water or incorporated into MCT-NEs, was assessed using a parallel artificial membrane permeability assay (PAMPA; BD Biosciences, San Jose, CA, USA). To prepare the test samples, each NE was diluted with phosphate-buffered saline (PBS, pH 6.8) to obtain final concentrations of 20 µg/mL for ATV and 80 µg/mL for DTX. For the PAMPA experiment, 300 µL of PBS (pH 6.8) containing 0.5% DMSO was placed into the receiver well, and 200 µL of the diluted formulation was added to the donor well. Following a 5-h incubation at room temperature, aliquots were collected from both compartments. ATV and DTX levels were quantified by HPLC as described previously. The *P_e_* was then computed using the following equation: *P_e_* = -*ln*(1-C_r_(t)/C_Eq_)/(A × (1/V_d_ + 1/V_r_)), where *P_e_* denotes the effective permeability (cm/s), and the permeation area is given by A = 0.288 cm^2^. The donor and receiver well volumes are represented as V_d_ = 0.2 mL and V_r_ = 0.3 mL, respectively. The variable t corresponds to the total incubation time (s). The drug concentration in the receiver compartment at time t is written as C_r_(t). The equilibrium concentration, C_Eq_, is defined as: C_Eq_ = (C_d_(t) × V_d_ + C_r_(t) × V_r_)/(V_d_ + V_r_), where C_d_(t) denotes the concentration remaining in the donor compartment at time t.

### *In vitro* apparent permeability across a Caco-2/HT-29-MTX-E12 cell monolayer

To evaluate the apparent permeability (*P_app_*) of ATV and DTX across an intestinal epithelial model, an 8:2 mixture of Caco-2 and HT-29-MTX-E12 cells (calculated by cell count) was used to establish the co-culture. Caco-2 cells (HTB-37^TM^; ATCC, Manassas, VA, USA) and HT-29-MTX-E12 cells (EACC 12040401; PHE, Oxford, UK) were suspended in DMEM supplemented with 10% (v/v) FBS and 1% (v/v) penicillin/streptomycin, and seeded onto 24-well transwell inserts at 1 × 10^5^ cells per well. The cultures were maintained for 14 days until a fully differentiated monolayer was established, indicated by a transepithelial electrical resistance (TEER) exceeding 350 Ω·cm^2^. After the differentiation period, the wells were washed and replaced with Hank's balanced salt solution (HBSS), followed by a 20-min equilibration at 37 °C. Each diluted formulation in HBSS (final concentrations: 20 µg/mL ATV and 80 µg/mL DTX) was administered to the apical chamber in a volume of 0.1 mL and 0.6 mL of HBSS was introduced into the basolateral chamber. Throughout the permeability assay conducted at 37 °C, a 200 µL aliquot was collected from each basolateral well at 0.5, 1, 2, 3, 4, and 5 h, after which the same volume of fresh HBSS was promptly added to restore the original volume. The TEER was measured again at the end of the assay to verify that the barrier properties of the cell layer were preserved. The post-experiment TEER values, averaging around 270 ± 30 Ω·cm^2^, demonstrated that the monolayer integrity was maintained throughout the study. The amount of ATV or DTX that traversed the cell layer was quantified by HPLC as described in the analytical section. Apparent permeability was then obtained by applying the equation* P_app_* = (dQ/dt)/(S × C_i_), in which dQ/dt represents the rate at which each drug appeared on the basolateral side, C_i_ denotes the initial apical concentration, and S corresponds to the effective surface area of the cell monolayer. The resulting value is expressed in centimeters per second and reflects the overall transport efficiency of the formulation.

### Intracellular uptake by Caco-2 and ASBT-transfected MDCK cells

To assess whether ASBT and SMVT contribute to the internalization of MCT-NEs, Caco-2 cells (5 × 10^4^ per coverslip) were plated onto Cell-Tak-coated substrates and cultured in DMEM containing 10% FBS and antibiotics until a consistent epithelial monolayer was formed. The monolayers were subsequently exposed to 100 µL of MCT-NEs co-loaded with coumarin 6, ATV (20 µg/mL), and DTX (80 µg/mL), followed by a 1-hour incubation at 37 °C. Afterward, the cells were rinsed with chilled PBS (pH 7.4), fixed in 4% formaldehyde, and labeled with 100 nM phalloidin-rhodamine and DAPI to visualize cytoskeletal structures and nuclei. Fluorescence uptake of coumarin 6 was then examined using confocal laser scanning microscopy (CLSM; Carl Zeiss, Oberkochen, Germany).

To confirm the involvement of specific transporters, MDCK cells (ATCC^®^ CCL-34^TM^) were plated at 1 × 10^4^ cells per coverslip and subsequently engineered to express human ASBT by transfecting SLC10A2 cDNA (OriGene Technologies, Inc., Rockville, MD, USA) with Lipofectamine 2000^®^ and P300^TM^ (Thermo Fisher). After achieving a confluent layer, the cells were exposed for 1 h to coumarin 6-labeled ATV/DTX (prepared in water or 0.5% DMSO) or to the corresponding MCT-NE formulation, with parallel treatments conducted in the presence of the SMVT blocker PA. Following incubation, the cells were washed, fixed with 4% formaldehyde, and subsequently permeabilized and blocked in PBS containing 0.3% Triton X-100 and 10% normal goat serum. ASBT expression was then visualized by sequential staining with a primary anti-ASBT antibody (1:500), an Alexa Fluor 546-conjugated secondary antibody (10 µg/mL), and DAPI. Fluorescent signals were examined using CLSM.

### Quantitative cellular uptake of ATV and DTX by Caco-2 and ASBT-transfected Madin-Darby canine kidney (MDCK) cells

The cellular uptake of ATV and DTX from MCT-NEs was quantitatively assessed in Caco-2 cells and MDCK cells, including ASBT-transfected and non-transfected variants. After reaching confluence in six-well plates, the cells were treated with one of the following: free ATV/DTX dispersed in water, the same drug combination dissolved in 0.5% DMSO, or an MCT-NE preparation adjusted to provide final concentrations of 20 µg/mL ATV and 80 µg/mL DTX. For transporter-targeting analysis, MDCK cells were treated with MCT-NEs incorporating DOCA-TAP, Biotinyl PE, or DCK/Biotinyl PE, with or without the SMVT inhibitor PA. Incubation was performed for 1 or 3 h at 37 °C. After incubation, the cells were washed thrice with ice-cold PBS (pH 7.4) to remove extracellular drug. Cells were harvested using trypsinization and centrifuged. The resulting cell pellets were subjected to methanol extraction by adding 1 mL of methanol to each sample. Following protein precipitation, the samples were centrifuged, and 1 mL of the supernatant was mixed with 100 µL of IS: simvastatin (1,000 ng/mL) for ATV or PTX (1,000 ng/mL) for DTX. After solvent removal under reduced pressure with a rotary evaporator, the dried extract was dissolved in 200 µL of a 1:1 (v/v) mixture of 10 mM ammonium formate and 0.04% formic acid, which served as the mobile phase.

For quantification of ATV, a 10-µL portion of the reconstituted sample was subjected to UPLC analysis on an ACQUITY system (Waters, Milford, MA) equipped with a BEH C18 column (2.1 × 100 mm, 1.7 µm). The separation was performed at 40 °C with a flow rate of 0.4 mL/min, applying an acetonitrile gradient (mobile phase B) that progressed from 30% at injection to 95% by 2.5 minutes and then returned to 30% between 5.0 and 6.0 minutes. Detection was performed using a Xevo TQ-S mass spectrometer (Waters Corp.) operated in positive-ion electrospray ionization mode with multiple reaction monitoring (MRM). ATV and IS were detected at *m/z* 559.2 → 440.2 and 436.3 → 285.2, respectively. Cone voltage and collision energy (CE) were 45 V and 22 eV for ATV, and 18 V and 13 eV for IS, respectively.

For DTX quantification, an ACQUITY BEH C18 column (50 × 2.1 mm, 1.7 µm) was used at 35 °C with a flow rate of 0.3 mL/min. The mobile phase comprised 10 mM ammonium formate in water with 0.2% formic acid (A) and acetonitrile with 0.2% formic acid (B). The gradient elution protocol was: 90% A (0-0.9 min), 100% B (0.9-1.6 min), and return to 90% A (1.6-2.5 min). DTX and PTX were detected at *m/z* 830.4 → 549.4 and 876.2 → 307.9, respectively, with cone voltages of 40 V (DTX) and 90 V (PTX), and CE values of 33 eV and 30 eV, respectively. The ion source operated at 150 °C, while the desolvation stage was maintained at 600 °C, accompanied by a desolvation gas flow rate of 1,000 L per hour.

### Intestinal transport mechanism of MCT-NEs

To investigate the mechanisms by which MCT-NEs cross the intestinal epithelial layer, transport experiments were performed using fully differentiated monolayers generated from a co-culture of Caco-2 and HT-29 MTX-E12 cells. In experiments assessing the effects of transporter blockade, the apical compartment was exposed to 0.1 mL of HBSS supplemented with the corresponding inhibitors listed in Table S3, while 0.6 mL of inhibitor-free HBSS was simultaneously applied to the basolateral compartment. After 30 min of preincubation at 37 °C, the apical solution was replaced with 0.1 mL of MCT-NE diluted in HBSS (equivalent to 20 µg/mL ATV and 80 µg/mL DTX), containing the same inhibitor, while 0.6 mL of newly prepared HBSS was added to the basolateral side. The cells were kept under standard conditions at 37 °C, and 200-µL samples were withdrawn from the basolateral chamber at designated time points (0.5, 1, 2, 3, 4, and 5 h) and replaced with an equal volume of fresh HBSS. Finally, the transported ATV or DTX was isolated using methanol, and the resulting extracts were analyzed by UPLC-MS/MS for quantitative determination.

### *In vitro* drug release and stability of MCT-NE#9

Dissolution testing for ATV and DTX released from the MCT-NE#9 formulation was performed using a United States Pharmacopeia (USP) Apparatus I (basket configuration). Size #00 gelatin capsules were packed either with the free drug mixture (2.5 mg ATV and 10 mg DTX) or with an equivalent dose of the MCT-NE#9 preparation. Each capsule was immersed in 500 mL of dissolution medium—either 0.1 N HCl (pH 1.2) or PBS (pH 6.8)—and, when required, supplemented with 2% (w/v) Tween 80 to ensure sink conditions. The dissolution test was performed at 37 ± 0.2 °C with the basket maintained at a rotation speed of 100 rpm. At scheduled sampling points, 0.1-mL portions of the medium were collected, passed through 0.45-µm polyvinylidene fluoride (PVDF) filters, and subsequently examined by HPLC, monitoring absorbance at 246 nm for ATV and 230 nm for DTX.

For the stability assessment, size #00 gelatin capsules containing MCT-NE#9 (equivalent to 2.5 mg of ATV and 10 mg of DTX) were placed in sealed high-density polyethylene containers and maintained under two environmental conditions: 25 ± 2 °C with 65 ± 5% RH, and 40 ± 2 °C with 75 ± 5% RH. Over a 4-week period, samples were removed at weekly intervals and examined for drug retention and for the 2-hour dissolution profile in 500 mL of 0.1 N HCl (pH 1.2) supplemented with 2% (w/v) Tween 80, using USP Apparatus I followed by HPLC analysis. In parallel, the colloidal properties of MCT-NE#9—including droplet diameter, PDI, and zeta potential—were measured after redispersion in water to monitor physical stability.

### *In vivo* oral absorption of MCT-NE#9 in rats

To evaluate systemic exposure to ATV and DTX following low-dose oral administration of the optimized formulation (MCT-NE#9), rats received 2.5 mg/kg of ATV and 10 mg/kg of DTX by gavage for pharmacokinetic profiling. For comparison, additional groups were dosed with ATV intravenously (2.5 mg/kg bolus), ATV suspended in water for oral delivery (2.5 mg/kg), DTX given intravenously (2.5 mg/kg bolus), or DTX administered orally as an aqueous dispersion (10 mg/kg). Blood was drawn from the femoral vein cannula at scheduled intervals, immediately centrifuged at 13,000 × *g* for 5 minutes, and the resulting plasma was stored at -80 °C until measurement. The concentrations of both agents in plasma were quantified by UPLC-MS/MS analysis.

### *In vitro* cytotoxicity of MCT-NE

To assess the cytotoxic effects of ATV/DTX delivered either in 0.5% DMSO, as the MCT-NE#9 formulation, or as the blank-NE#9 vehicle, 4T1 cells were subjected to both the maximum tolerated dose (MTD) and MCT dosing schedules, and cell viability was assessed with the WST-8 colorimetric assay (Dojindo, Rockville, MD). Cells were first seeded into 96-well plates at 1 × 10^4^ cells per well in 100 µL of DMEM enriched with 10% FBS and 1% penicillin/streptomycin, followed by a 24-hour attachment period at 37 °C. Subsequently, 100 µL of the designated test sample—ATV/DTX dissolved in 0.5% DMSO, MCT-NE#9, or the blank NE—was dispensed into each well. All formulations were prepared to yield final drug concentrations of 0.1, 0.5, 1, 5, 10, 50, 100, or 200 µg/mL. For MCT groups, the medium was replaced every 24 h with fresh medium containing the same treatment concentrations for a total of 72 h. In the MTD group, the medium was changed at intervals of 24 h and 48 h only. Cell viability was assessed by adding 10 µL of WST-8 solution to 100 µL of DMEM in each well, then incubating for 2 h. Absorbance at 450 nm was measured using a microplate reader (Multimode Plate Reader; PerkinElmer, Waltham, MA, USA). Cell viability percentages were calculated by comparing the absorbance values of treated cells with those of untreated controls, which were considered 100% viable.

### *In vitro* cell adhesion, invasion, and migration assays of MCT-NE

The inhibitory effects of ATV/DTX and MCT-NEs on 4T1 cell adhesion to the extracellular matrix (ECM) were evaluated using a 48-well ECM Cell Adhesion Kit (CytoSelect^TM^; Cell Biolabs, Inc., San Diego, CA, USA) following the manufacturer's instructions. Briefly, 4T1 cells (1 × 10^6^ per well) were plated onto wells that had been pretreated with ECM components—fibronectin, collagen II, collagen IV, laminin I, or fibrinogen—while bovine serum albumin-coated wells served as the negative control surface. Subsequently, the cells were exposed to one of several treatment preparations: ATV/DTX dissolved in 0.5% DMSO at either 8.95 µM ATV plus 27.8 µM DTX or 17.9 µM ATV plus 49.5 µM DTX; the ATV-NE#9 formulation providing 8.95 µM ATV; the DTX-NE#9 formulation delivering 24.8 µM DTX; or the combined MCT-NE#9 system containing 8.95 µM ATV and 27.8 µM DTX. Certain experimental sets received drug exposure every third day, whereas the MCT regimen involved dosing once every 24 hours for a total of 72 hours. For the MTD condition, a single treatment was applied at the 24-hour mark, and the medium was refreshed at 48 hours without any additional dosing. At the end of the 72-hour period, the cells were rinsed with PBS (pH 7.4), incubated with the staining reagent for 10 minutes, gently rinsed with sterile water, and then subjected to the extraction buffer. Following a 10-minute shaking step, absorbance at 560 nm was recorded to determine the extent of cell adhesion inhibition.

*In vitro* invasive and migratory capacities were evaluated using the Cultrex^®^ 24-well basement membrane extract (BME) invasion system (Trevigen, Gaithersburg, MD, USA). For the invasion setup, each transwell insert was pre-coated with 100 µL of diluted (0.5×) BME and allowed to stabilize at 37 °C, whereas inserts left uncoated served as the migration controls. 4T1 cells suspended in RPMI supplemented with 0.5% FBS were plated at a concentration of 1 × 10^6^ cells in each insert, and 500 µL of RPMI containing 10% FBS was added to the basal compartment as a chemoattractant. Following a 24-h incubation, both compartments were washed, and MTD-treated cells received the drug for 24 h followed by fresh medium replacement every 24 h for an additional 48 h. In MCT-treated cells, fresh ATV/DTX or NE#9 were administered every 24 h for 2 consecutive days. After treatment, calcein-AM-containing cell dissociation solution was added to both compartments, and after 1-h incubation at 37 °C, fluorescence was measured at excitation/emission wavelengths of 485/520 nm to quantify invasion and migration.

### *In vitro* ferroptosis marker analysis

To evaluate the induction of ferroptosis *in vitro*, 4T1 cells were seeded at a density of 5 × 10^4^ cells per well in 12-well plates and treated for 72 hours under five different conditions: untreated control, blank-NE (MCT), free ATV/DTX at high dose (maximum tolerated dose [MTD], ATV 5 µg/mL + DTX 20 µg/mL), free ATV/DTX at low dose (MCT, ATV 1 µg/mL + DTX 4 µg/mL), and MCT-NE#9 (same dose as MCT). While the MTD group received a single treatment at 24 hours, the MCT and MCT-NE#9 groups were treated daily for 3 days. After 72 hours, cells were harvested and stained with specific fluorescent probes to assess key ferroptosis-related markers: 100 nM FerroOrange (Dojindo, Kumamoto, Japan) for intracellular ferrous iron (Fe^2+^), 10 µM H_2_DCF-DA (Thermo Fisher Scientific) for ROS, 2 µM BODIPY-C11 581/591 (Thermo Fisher Scientific) for LPO, and PE-conjugated anti-mouse CD71 antibody (1:200 dilution) for transferrin receptor expression. Staining was performed for 30 minutes in the dark at 37 °C for FerroOrange, H_2_DCF-DA, and BODIPY-C11, and at 4 °C for CD71. Fluorescence intensities were then measured using flow cytometry.

To further confirm the activation of ferroptotic signaling at the protein level, western blot analysis was conducted. 4T1 cells were seeded in 6-well plates at a density of 1 × 10^5^ cells per well and treated under four different conditions: untreated control, MTD, MCT, and MCT-NE#9. While the MTD group received a single treatment at 24 hours, the MCT and MCT-NE#9 groups were treated daily for 3 days. Following treatment, cells were lysed with 100 µL of CETi lysis buffer (TransLab, Daejeon, Korea), and lysates were centrifuged at 13,000 rpm for 15 minutes. Protein concentration in the supernatant was determined by bicinchoninic acid assay. Equal amounts of protein were separated by SDS-PAGE using a 12% gel and transferred to PVDF membranes. The membranes were blocked with 5% skim milk (BD DIFCO^TM^, Franklin Lakes, NJ, USA) for 1 h and incubated overnight at 4 °C with primary antibodies (diluted 1:1000 in TBST). After washing, membranes were incubated with appropriate secondary antibodies for 1 h at room temperature and visualized using a chemiluminescent substrate (West Pico Plus).

To assess the immunogenic release of DAMPs, HMGB1 levels in cell culture supernatants were quantified by ELISA using the HMGB1 ELISA KIT (Elabscience, Houston, TX, USA). For this assay, 4T1 cells were seeded at 2 × 10^5^ cells per well in 6-well plates and treated according to the MTD or MCT protocols. After 72 h, supernatants were collected, and 200 µL from each sample was added to ELISA plates pre-coated with capture antibodies. Plates were incubated at 37 °C for 2 h, followed by five washes with PBST. Detection antibody was added and incubated at 37 °C for 1 h, followed by horse radish peroxidase (HRP)-conjugated secondary antibody for 30 min. After additional washes, TMB substrate was added and incubated for 20 minutes before stopping the reaction. Absorbance was measured using a microplate reader to quantify HMGB1 release.

### *In vitro* phagocytosis assay

To assess macrophage TLR2 recognition of ferroptosis-induced “eat-me” signals, 4T1 cells were first stained with CellTracker^TM^ Deep Red (Thermo Fisher Scientific) and seeded at 5 × 10^4^ cells per well. Cells were then treated for 24 h under three conditions: RSL3 (0.5 μM) to induce ferroptosis, STS (5 μM) to induce apoptosis, and MCT-NE#9. In parallel, cells treated with MCT-NE#9 received the NE formulation (equivalent to ATV 1 µg/mL + DTX 4 µg/mL). In designated wells, Fer-1 (20 μM) was added 3 h before other treatments to block ferroptosis. After treatment, cells were washed, detached, and co-cultured with LPS-activated Raw 264.7 macrophages (CellTracker^TM^ Green CMFDA (Thermo Fisher Scientific, Waltham, MA, USA); 1 × 10^6^ cells per dish) at a 1:5 ratio for 3 h at 37 °C. CU-CPT-22 (20 μM) was pretreated 3 h before LPS exposure to blockade TLR2. Phagocytosis of Red-labeled 4T1 cells by Green-labeled macrophages was quantified by flow cytometry.

For additional phagocytosis assays, 4T1 cells were stained and treated according to the detailed MTD and MCT protocols described above, ensuring clarity in dosing schedule and drug formulation. Raw 264.7 cells were activated with LPS (100 ng/mL) for 24 h and stained with CellTracker^TM^ Green. After detachment, the two cell populations were mixed and incubated for 3 h at 37 °C. Phagocytic uptake was then analyzed by flow cytometry, gating on double-positive events.

### *In vitro* macrophage activation assay

To analyze macrophage activation following phagocytosis, 4T1 cells and Raw 264.7 cells were co-cultured and analyzed using macrophage activation markers. 4T1 cells were seeded in 12-well plates at a density of 5 × 10^4^ cells per well and treated for 24 h with MCT-NE#9 containing an equivalent concentration of ATV 1 µg/mL + DTX 4 µg/mL. Raw 264.7 cells were seeded in 100-mm culture dishes at a density of 1 × 10^6^ cells per dish and pretreated with CU-CPT-22 (20 µM), a TLR2 inhibitor, 3 h prior to co-culture. After the removal of supernatant from 4T1 wells, the Raw 264.7 cells were collected and added directly to the 4T1 cell monolayer and co-cultured for 24 h at 37 °C. Following co-culture, all cells were harvested using 1× trypsin, washed, and stained with fluorochrome-conjugated antibodies against CD11b, MHC class I, MHC class II, CD86, and inducible nitric oxide synthase (iNOS), each at a 1:200 dilution. Staining was performed in the dark at 4 °C for 30 min. After washing, the expression levels of macrophage activation markers were analyzed by flow cytometry.

### *Ex vivo* fluorescence-activated cell sorting (FACS) analysis

To evaluate immune cell activation and antigen presentation following cancer cell ferroptosis, B16F10.OVA melanoma cells and splenocytes from C57BL/6 mice were used. B16F10.OVA cells were seeded in 12-well plates at a density of 5 × 10^4^ cells per well. Drug treatments were administered according to the MTD and MCT regimens. Spleens were isolated from C57BL/6 mice, and splenocytes were extracted using a 70-μm strainer. To remove erythrocytes, 1× RBC lysis buffer (Thermo Fisher Scientific) was added to the splenocytes and incubated at room temperature for 10 min, followed by centrifugation at 1700 rpm for 3 min. After drug treatment, 5 × 10^6^ splenocytes were co-cultured with B16F10.OVA cells for 24 h. After co-culture, both supernatant and cells were collected and centrifuged together. Cells were then washed and stained with the appropriate markers for flow cytometric analysis to identify CD4 T cells (CD45^+^, CD3^+^, CD4^+^), CD8 T cells (CD45^+^, CD3^+^, CD8^+^), T_regs_ (CD45^+^, CD3^+^, CD4^+^, FoxP3^+^), NK cells (CD45^+^, CD3^-^, NK1.1^+^), NKT cells (CD45^+^, CD3^+^, NK1.1^+^), M1 macrophages (CD45^+^, CD11b^+^, F4/80^+^, CD86^+^, iNOS^+^), M2 macrophages (CD45^+^, CD11b^+^, F4/80^+^, Arginase^+^), antigen-presenting macrophages (CD45^+^, CD11b^+^, F4/80^+^, MHC-1 SIINFEKL^+^), dendritic cells (CD45^+^, CD11b^+^, CD11c^+^), and antigen-presenting dendritic cells (CD45^+^, CD11c^+^, MHC-1 SIINFEKL^+^).

### *In vivo* biodistribution and toxicity evaluation of MCT-NE#9

The biodistribution of ATV and DTX was evaluated in 4T1 tumor-bearing mice. When tumor volumes reached approximately 80-100 mm^3^, treatment was initiated. Two treatment regimens were evaluated: the MTD protocol delivered intravenously (ATV 2.5 mg/kg and DTX 10 mg/kg every three weeks) and the daily oral MCT-NE#9 regimen containing the same drug doses. On day 22, animals were euthanized at scheduled intervals following administration (0.5, 1, 2, 4, 6, 8, 10, and 24 hours). Blood samples were obtained via the retro-orbital route and transferred into tubes pre-coated with anticoagulant, followed by centrifugation at 2,500 × *g* for 15 minutes at 4 °C. The resulting plasma fraction was isolated and preserved at -80 °C for later analysis. Additionally, key tissues—including the liver, heart, lungs, kidneys, spleen, and tumor—were harvested, briefly washed with chilled PBS (pH 7.4), gently blotted, rapidly frozen in liquid nitrogen, and stored at -80 °C until further processing.

Extraction and quantification of ATV and DTX were conducted for both plasma and tissue samples. Plasma extraction and UPLC-MS/MS analyses were performed as described in the previous section. For tissue samples, approximately 100 mg of each frozen organ was homogenized in 1 mL of acetonitrile:isopropanol (70:30, v/v) containing 0.1% formic acid using a homogenizer on ice, followed by centrifugation at 14,000 × *g* for 10 min at 4 °C. The supernatants were collected, evaporated to dryness under a gentle nitrogen stream, and reconstituted in 100 µL of methanol:water (60:40, v/v, 0.1% formic acid). After a final centrifugation, the clear extracts were analyzed by UPLC-MS/MS as described earlier, and ATV and DTX concentrations were determined using validated calibration curves.

Next, systemic toxicity of ATV and DTX was also evaluated in 4T1 tumor-bearing mice. When tumor volumes reached approximately 50-60 mm^3^, treatment was initiated and continued for 4 weeks. The study included four cohorts: a healthy control group receiving saline, a tumor-bearing control group also treated with saline, an IV MTD group administered ATV (2.5 mg/kg) and DTX (10 mg/kg) every three weeks, and an oral MCT-NE#9 group receiving the same doses on a once-daily schedule.

At the end of treatment, blood samples were collected for serum biochemical analysis. Hepatic toxicity was evaluated using markers of liver function, including alanine aminotransferase (ALT), aspartate aminotransferase (AST), alkaline phosphatase (ALP), and total bilirubin (TBIL). Renal toxicity and electrolyte imbalance were assessed based on blood urea nitrogen (BUN), creatinine (CRE), inorganic phosphorus (PHOS), and calcium (Ca) levels. In addition, the small intestine, liver, heart, kidneys, lungs, and spleen were excised; fixed in 4% formalin; embedded in paraffin; sectioned; and stained with hematoxylin and eosin (H&E) for histopathological evaluation.

### *In vivo* tumor growth inhibition of MCT-NEs

To evaluate the antitumor activity of the orally administered co-delivery systems, 7-week-old female BALB/c mice were inoculated in the right dorsal flank with 4T1 breast cancer cells (1 × 10^6^ cells in 100 μL PBS, pH 7.4). Once tumors expanded to approximately 50-60 mm^3^, animals were randomized into eight treatment groups (n = 10 per group). These included: an untreated control; an IV MTD cohort receiving ATV (2.5 mg/kg) and DTX (10 mg/kg) every three weeks; an oral ATV/DTX group dosed daily at 2.5 mg/kg ATV and 10 mg/kg DTX; an ATV-NE#9 group given 2.5 mg/kg ATV orally each day; a DTX-NE#9 cohort receiving 10 mg/kg DTX orally; a daily oral MCT-NE#9 group delivering 2.5 mg/kg ATV and 10 mg/kg DTX; an anti-CD47 group administered 10 mg/kg intraperitoneally every three days; and a combination arm treated with both oral MCT-NE#9 and anti-CD47. Body weight and tumor growth were monitored at 3-day intervals, and tumor volume was calculated using the following equation: (width)^2^ × length × 0.52. The mice were monitored daily, and those exhibiting signs of distress or bearing tumors exceeding 10% of body weight (approximately 2000 mm^3^) were euthanized upon meeting the predefined endpoint criteria, as required by the approved IACUC protocol. Following a 21-day treatment period, the animals were euthanized, and the excised tumors were weighed for quantitative assessment.

### Immunohistochemistry (IHC)

Immunohistochemical staining was conducted using a commercial IHC kit (Acro Biosystems, Newark, DE, USA) following the manufacturer's instructions. Endogenous peroxidase activity was blocked using the hydrogen peroxide solution provided in the kit. After blocking with normal serum, the sections were incubated overnight at 4 °C with primary antibodies against GPX4 (1:100 dilution; Abcam, Cambridge, UK) and CD71 (1:100 dilution; Invitrogen, Waltham, MA, USA).

### RNA isolation

Total RNA extraction was carried out with TRIzol reagent (Invitrogen). The integrity of the isolated RNA was verified using the TapeStation 4000 system (Agilent Technologies, Amstelveen, The Netherlands), and RNA concentrations were subsequently determined with an ND-2000 spectrophotometer (Thermo Fisher Scientific).

### Library preparation and sequencing

Library construction was carried out using total RNA as input with the CORALL RNA-Seq V2 Library Prep Kit (LEXOGEN, Vienna, Austria). mRNA species were enriched via the Poly(A) RNA Selection Kit (LEXOGEN), after which the purified transcripts were subjected to cDNA synthesis and fragmentation according to the manufacturer's guidelines. Sample indexing employed Illumina index sets 1-12, and PCR amplification was used to enrich the resulting libraries. Quality assessment of fragment size was performed using the TapeStation HS D1000 ScreenTape system (Agilent Technologies). Final library quantification was obtained with a dedicated library quantification kit on the StepOne Real-Time PCR platform (Life Technologies, Carlsbad, CA, USA). High-throughput sequencing was performed as paired-end 100 sequencing using NovaSeq 6000 (Illumina, San Diego, CA, USA). Quality control of raw sequencing data was performed using FastQC. Adapter and low-quality reads were removed using Fastp. The trimmed reads were then mapped to the reference genome using STAR. The quantification of reads was done using Salmon. The read counts were processed based on the trimmed mean of m-values (TMM) + counts per million (CPM) normalization method using EdgeR. Data mining and graphic visualization were performed using ExDEGA (Ebiogen Inc., Seoul, Korea).

### *In vivo* immunomodulatory effects of MCT-NE#9

To evaluate the immunostimulatory potential of the oral metronomic MCT-NE#9 regimen *in vivo*, BALB/c mice were inoculated in the right flank with 4T1 cells (1 × 10^6^ in suspension). Once tumors expanded to roughly 60-70 mm^3^, animals were randomized into three groups (n = 10 each): an untreated control cohort; an IV MTD group receiving 2.5 mg/kg ATV plus 10 mg/kg DTX every three weeks; and a daily oral MCT-NE#9 group administered the same drug doses. Tumor growth and body weight were recorded at three-day intervals. After 3 weeks of treatment, mice were sacrificed, and tumors were excised. Tumor tissues and lymph nodes were dissociated using a mixture of collagenase (20 mg/mL), dispase (10 mg/mL), and DNase (10 mg/mL) to degrade connective tissues. Then, samples were centrifuged at 2000 rpm for 5 min, and the supernatant was discarded. RPMI 1640 medium supplemented with 10% FBS was added, and immune cells were isolated using Histopaque. Isolated immune cells were subsequently stained with specific FACS antibodies for analysis.

Tumor tissues were stained to identify various immune cell populations and associated markers, including T-cell activation markers (CD45⁺, CD3⁺, CD25⁺), T_regs_ (CD45⁺, CD3⁺, FoxP3⁺), myeloid-derived suppressor cells (MDSCs) (CD45⁺, CD11b⁺, Gr-1⁺), NK cells (CD45⁺, CD3⁻, CD49b⁺), M1 macrophages (CD45⁺, CD11b⁺, F4/80⁺, iNOS⁺), and M2 macrophages (CD45⁺, CD11b⁺, F4/80⁺, Arginase⁺), as well as non-immune markers such as CD71 (CD45⁻), CD47 (CD45⁻), and LPO (CD45⁻, BODIPY C11 581/591⁺). For peripheral immune cell analysis, cardiac blood was drawn from each mouse. The collected samples were then treated with 1× RBC lysis buffer for 10 minutes to eliminate red blood cells, after which they were centrifuged at 1700 rpm for 3 minutes. Cells were then stained with markers for flow cytometric analysis, including CD4 T cells (CD45^+^, CD3^+^, CD4^+^), CD8 T cells (CD45^+^, CD3^+^, CD8^+^), CD11b^+^ cells (CD45^+^, CD11b^+^), granulocytes (CD45^+^, CD11b^+^, Ly6G^+^, Ly6C^-^), and monocytes (CD45^+^, CD11b^+^, Ly6G^-^, Ly6C^+^).

In addition, immune populations within the tumor-draining lymph nodes (TDLNs) were profiled by staining for markers of CD4 T cells (CD45⁺, CD3⁺, CD4⁺), CD8 T cells (CD45⁺, CD3⁺, CD8⁺), NK cells (CD45⁺, CD3⁻, CD49b⁺), and NKT cells (CD45⁺, CD3⁺, CD49b⁺). This comprehensive staining approach allowed a detailed analysis of the immunological effects of the treatments, focusing on the activation and presence of various immune cell types within the TME and TDLNs.

### Pharmacokinetic and statistical analyses

Pharmacokinetic analysis was conducted in WinNonlin^®^ (version 5.3; Pharsight Corporation, Mountain View, CA, USA) using a non-compartmental model to obtain the corresponding pharmacokinetic parameters. Data are presented as mean ± standard deviation (SD) or standard error of the mean (SEM). Statistical comparisons among three or more groups were conducted using one-way analysis of variance, followed by Tukey's multiple comparison test. Values of p < 0.05 were considered statistically significant in all analyses.

## Results

### Preparation and characterization of MCT-NEs

To identify the most effective mixing proportion of ATV and DTX for combination treatment, cytotoxic responses were evaluated at constant overall drug concentrations while varying the ATV-to-DTX ratios. Each drug, used either alone or in combination, exhibited concentration-dependent cytotoxicity. The 0.25:1 (w/w) ratio showed the highest cytotoxicity and the lowest IC_50_ values [1.11-, 1.75-, 1.65-, and 1.36-fold lower than those for ATV/DTX (0.5:1), ATV/DTX (1:0.25), ATV/DTX (1:0.5), and ATV/DTX (1:1), respectively], outperforming single-drug treatments and other combinations, particularly due to the higher DTX content (Figure S1). This ratio was selected for nanoplatform formulation.

DSC analysis was conducted to confirm ionic complex formation between DCK and Biotinyl PE. DCK exhibited two broad thermal events—one in the range of 105-130 °C and another above 225 °C—indicating that DCK exists predominantly in an amorphous state. Biotinyl PE showed a broad transition around 165 °C, also characteristic of an amorphous material. The physical mixture of DCK and Biotinyl PE presented only subtle shifts in the endothermic pattern to approximately 120 °C, suggesting minimal thermal interaction between the two components. In contrast, the DSC thermogram of the DCK/Biotinyl PE complex revealed a sharp endothermic peak at approximately 245 °C, which was absent in both the individual components and their physical mixture. This distinct thermal event suggests the formation of a unique crystalline phase, likely arising from ionic interactions between DCK and Biotinyl PE (Figure S2A). The formation of the ionic complex was further confirmed by FT-IR spectroscopy. The FT-IR spectrum of DCK showed characteristic peaks at 2,924 and 2,861 cm^-1^ (C-H stretching), 1,645 cm^-1^ (amide I band), and 1,539 cm^-1^ (symmetric bending of the NH_3_^+^ group). Biotinyl PE exhibited typical absorption bands at 2,922 and 2,853 cm^-1^ (C-H stretching), a strong C=O stretching band at 1,688 cm^-1^ (urea group), and a prominent phosphate (PO_2_^-^) peak at 1,065 cm^-1^. The spectral profile of the physical mixture resembled a direct overlay of the two separate components, suggesting that little to no interaction occurred between them. However, the FT-IR spectrum of the DCK/Biotinyl PE complex displayed pronounced spectral changes, including enhanced NH_3_^+^ asymmetric and symmetric bending bands at 1,647 and 1,540 cm^-1^, respectively. Notably, the phosphate peak of Biotinyl PE shifted from 1,065 cm^-1^ to 1,043 cm^-1^ and became considerably sharper (Figure S2B). These spectral shifts, particularly in the amine and phosphate vibrational regions, strongly support the formation of an ionic complex via electrostatic interactions between the cationic DCK and the anionic Biotinyl PE.

For enhanced solubility and loading, ATV and DTX were encapsulated in Capryol 90, with S_mix_ (Tween 80:Transcutol HP, 1:2 [w/w]) as the emulsifier (MCT-NE#1). Transporter-targeting agents (e.g., TPGS, DCK, DOCA, Biotinyl PE) were incorporated to improve intestinal absorption (Table S2). All MCT-NEs formed stable oil-in-water (o/w) NEs with high encapsulation efficiency (~100%), except for formulation #5 (Table S4). Particle sizes of MCT-NEs ranged from 8.7 to 44 nm. Moreover, positively charged additives (DCK, DOTAP) increased zeta potentials, while negatively charged components (DOCA, Biotinyl PE) decreased them. This result confirmed its anchorage onto the oil droplets with surfactant and co-surfactant, as well as the exposure of phosphatidylethanolamine and DOTAP toward the inner oil phase, and DOCA or DCK and Biotin toward the outer aqueous phase.

**Figure 1:**
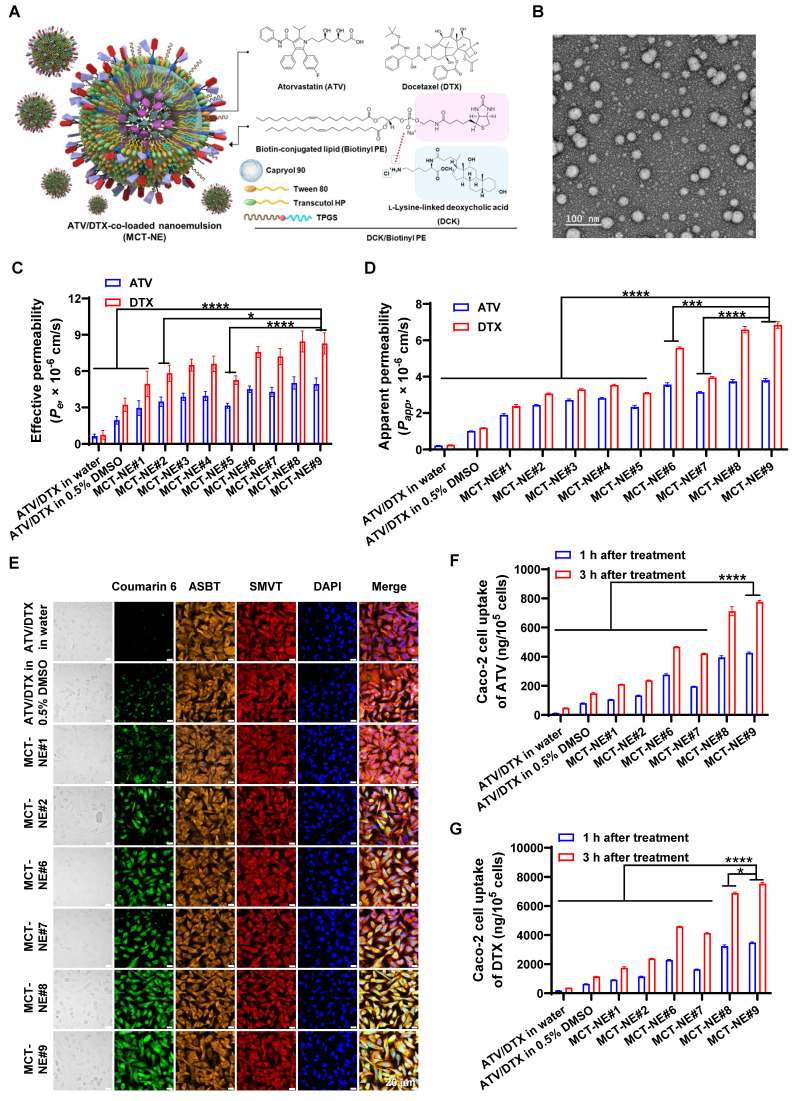
**Preparation and characterization of MCT-NEs.** (**A**) Schematic representation of ATV/DTX-loaded MCT-NEs functionalized with DCK/Biotinyl PE. (**B**) Transmission electron micrograph of the MCT-NE#9 formulation (scale bar: 100 nm). (**C**) *In vitro* effective permeability (*P_e_*) of ATV, DTX, and MCT-NE variants measured using an artificial intestinal membrane (mean ± SD, n = 7). (**D**) Apparent permeability (*P_app_*) of ATV, DTX, and MCT-NEs across Caco-2/HT-29 MTX-E12 cocultures (mean ± SD, n = 3). (**E**) Confocal images showing cellular uptake of coumarin 6-labeled ATV/DTX delivered in water, 0.5% DMSO, or MCT-NEs (scale bar: 20 μm). Quantified intracellular levels of (**F**) ATV and (**G**) DTX after exposure to ATV/DTX in water, 0.5% DMSO, or MCT-NEs (mean ± SD, n = 3). * p < 0.05, *** p < 0.001, **** p < 0.0001.

The final optimized formulation, MCT-NE#9, incorporating the DCK/Biotinyl PE ionic complex, demonstrated a mean droplet diameter of 15.8 ± 0.065 nm, a PDI of 0.332 ± 0.011, and complete (100%) encapsulation of the loaded drugs (Figure 1A, Table S4). TEM confirmed uniform nanoscale morphology (< 50 nm) (Figure 1B), indicating enhanced thermodynamic stability via interfacial energy reduction by S_mix_.

### *In vitro* permeability of MCT-NEs

To assess enhanced intestinal permeability in association with improved solubility, the artificial membrane permeability (*P_e_*) of ATV and DTX was measured. NE formulation (MCT-NE#1) increased *P_e_* values by 358% and 568%, respectively, compared with drug dispersion in water, attributed to improved solubility (Figure 1C). Further incorporation of TPGS, DCK, or DOCA (MCT-NE#2-4) yielded additional *P_e_* increases of 17.6%, 31.5%, and 33.6% for ATV and 17.8%, 31.4%, and 33.6% for DTX, respectively. Furthermore, the inclusion of DOCA-TAP, Biotinyl PE, or their combination (MCT-NE#6-8) led to greater enhancements of *P_e_*, being 52.8%, 45.1%, and 70.2% higher for ATV, and 53.2%, 45.7%, and 71.1% higher for DTX, respectively, compared to MCT-NE#1, likely due to increased membrane fluidity. MCT-NE#9 (DCK/Biotinyl PE) showed 67% higher *P_e_* for both drugs over MCT-NE#1, and 664% (ATV) and 1,019% (DTX) increases over the respective drug-in-water.

To evaluate transporter-facilitated uptake, the *P_app_* across Caco-2/HT-29-MTX-E12 monolayers was examined. MCT-NE#1 increased *P_app_* by 778% (ATV) and 828% (DTX) versus water dispersion (Figure 1D). Additives like TPGS, DCK, and DOCA (MCT-NE#2-4) further improved *P_app_* by up to ~48%, likely via barrier modulation and P-gp inhibition. DOCA-TAP (MCT-NE#6) and Biotinyl PE (MCT-NE#7) enhanced *P_app_* significantly through ASBT and SMVT pathways, with MCT-NE#8 showing synergistic effects. MCT-NE#9 exhibited the highest increases—101% (ATV) and 187% (DTX) over MCT-NE#1—corresponding to 1,662% and 2,563% increases over drug-in-water. These results underscored the synergistic impact of NE-mediated solubility enhancement and transporter-targeted uptake via DOCA- and biotin-conjugated lipid (DCK/Biotinyl PE).

### Cellular uptake of coumarin 6-co-loaded MCT-NE by Caco-2 and ASBT-transfected MDCK cells

Confocal microscopy confirmed enhanced intracellular uptake of MCT-NEs in Caco-2 cells, consistent with the *in vitro* permeability data. MCT-NE#2 (with coumarin 6) showed greater uptake than free drugs or MCT-NE#1, owing to improved solubility and membrane interactions from Tween 80, Transcutol HP, and TPGS (Figure 1E) 43,44. Incorporating DOCA-TAP or Biotinyl PE (MCT-NE#6 and #7) further increased coumarin 6 uptake, suggesting ASBT- and SMVT-mediated transport. Dual incorporation of DOCA-TAP and Biotinyl PE (MCT-NE#8), or DCK/Biotinyl PE (MCT-NE#9), showed the greatest uptake. At 3 h after treatment with MCT-NE#9, the intracellular levels of ATV were 15.9-, 3.68-, 1.66-, and 1.84-fold higher, and the levels of DTX were 19.9-, 4.32-, 1.64-, and 1.82-fold higher, relative to those in cells treated with free drug and MCT-NE#1, #6, and #7 (Figure 1F, G), respectively, confirming synergistic uptake via ASBT and SMVT. Although MCT-NE#8 and MCT-NE#9 exhibited comparable performance across the screening endpoints, a pre-specified design-based selection criterion was applied to advance MCT-NE#9. Specifically, MCT-NE#9 co-displays the bile acid (DCK) and biotin (Biotinyl PE) ligands as a single ionic complex, enabling co-localized and stable surface presentation while minimizing batch-to-batch variability relative to the co-inclusion approach used in MCT-NE#8. In addition, MCT-NE#9 demonstrated tighter quality attributes, including uniform ~16-nm droplet size, low PDI, and near-quantitative drug loading. Based on these physicochemical and formulation advantages, MCT-NE#9 was selected as the optimized NE and used as the representative formulation for transporter-targeting, pharmacokinetic, and efficacy studies.

**Figure 2:**
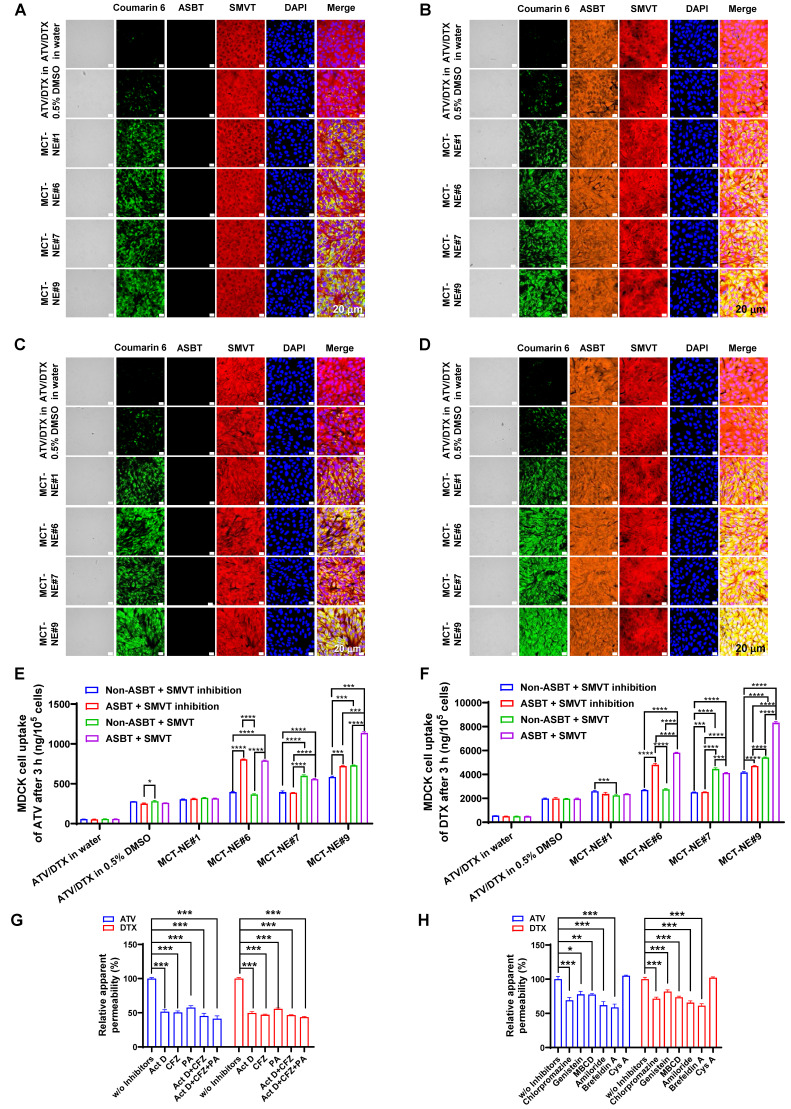
***In vitro* transport mechanism of MCT-NEs.** Confocal micrographs show coumarin 6-labeled ATV/DTX uptake from water, 0.5% DMSO, and MCT-NEs in (**A**) ASBT-negative MDCK cells treated with the SMVT inhibitor, (**B**) ASBT-expressing MDCK cells treated with the SMVT inhibitor, (**C**) ASBT-negative MDCK cells without SMVT inhibition, and (**D**) ASBT-expressing MDCK cells in the absence of SMVT inhibition (scale bar: 20 μm). Quantitative uptake of (**E**) ATV and (**F**) DTX after 3 h exposure to ATV/DTX in water, 0.5% DMSO, or MCT-NEs is reported as mean ± SD (n = 3). Relative permeability of MCT-NE#9 across Caco-2/HT-29 MTX-E12 monolayers is shown (**G**) under selective inhibition of ASBT, OST_α/β_, and SMVT alone or in combination, and (**H**) following incubation with mechanistic transcytosis inhibitors (mean ± SD, n = 3). * p < 0.05, ** p < 0.01, *** p < 0.001, **** p < 0.0001.

To confirm dual-transporter involvement, MDCK cells overexpressing ASBT, together with their non-transfected controls, were treated with MCT-NEs containing coumarin 6, either in the presence or absence of the SMVT blocker PA. Minimal uptake was seen for free drugs or MCT-NE#1, regardless of ASBT or SMVT expression (Figure 2A-D). In contrast, MCT-NE#6 showed significantly greater uptake in ASBT-expressing cells (with SMVT inhibition), with 2.10- and 1.79-fold higher intracellular ATV and DTX levels at 1 and 3 h, respectively, versus non-transfected controls (Figure 2E, F, S3). MCT-NE#7 displayed clear reliance on SMVT activity; in MDCK cells lacking ASBT, removal of the SMVT inhibitor led to elevated intracellular ATV and DTX by 1.45-1.78-fold at 1 and 3 h (Figure 2E, F, S3).

MCT-NE#9 exhibited the strongest dual-targeting effect. In ASBT-expressing MDCK cells without SMVT inhibition, coumarin 6 uptake significantly exceeded that in cells with SMVT inhibition and ASBT-negative cells (Figure 2A-D). At 3 h, in ASBT-positive MDCK cells without SMVT blockade, intracellular levels of ATV and DTX were 1.58-1.77 times greater than when SMVT was inhibited, and roughly 1.95-2.00 times higher compared with ASBT-negative cells treated concurrently with the inhibitor (Figure 2E, F). These findings confirmed that MCT-NE#9 facilitates synergistic ASBT- and SMVT-mediated drug delivery through DCK- and biotin-conjugated lipids.

### Intestinal transport mechanism of MCT-NE

To validate the transport mechanisms of MCT-NE#9, inhibitors of ASBT (Act D), SMVT (PA), and organic solute transporter (OST) α and β (OST_α/β_) (CFZ) were used in a Caco-2/HT-29-MTX-E12 monolayer model (Figure 2G). ASBT inhibition by Act D reduced the *P_app_* of ATV and DTX by ~50%. CFZ also decreased *P_app_* by ~50-60%, confirming OST_α/β_ involvement in basolateral transport. When CFZ was combined with Act D, further reductions were observed. SMVT inhibition with PA lowered *P_app_* by ~42-44%, and simultaneous inhibition of ASBT, SMVT, and OST_α/β_ led to a significant decrease of ~56-59% (Figure 2G). These findings indicated that MCT-NE#9 absorption involves multiple transporters: ASBT and SMVT mediate apical uptake, while OST_α/β_ facilitates basolateral efflux. The DCK/Biotinyl PE component of MCT-NE#9 enables targeted interaction with both ASBT and SMVT, enhancing cellular uptake through mimicry of natural ligands.

To explore additional uptake mechanisms of MCT-NE#9, various inhibitors were tested. Clathrin-mediated endocytosis was confirmed as a major route, as chlorpromazine reduced *P_app_* by ~30% for both ATV and DTX (Figure 2H). Caveola/lipid raft-mediated uptake was also involved, with genistein and MBCD decreasing *P_app_* by ~18-27%. Amiloride, a macropinocytosis inhibitor, further reduced permeability by ~34-38%. In contrast, P-gp efflux had minimal impact, with net apparent permeability ratio (NAPR) values close to 1, likely due to the presence of TPGS in the NE system, which inhibits P-gp. Finally, brefeldin A reduced *P_app_* by ~39-41%, suggesting that ER-Golgi-associated routes contribute to the intracellular trafficking process (Figure 2H). Overall, MCT-NE#9 was internalized via multiple endocytic routes and trafficked intracellularly through endoplasmic reticulum (ER)/Golgi-mediated processes, enhancing its transcellular transport across the intestinal epithelium.

### *In vitro* drug dissolution and stability of MCT-NE#9-loaded capsules

The dissolution behavior of MCT-NE#9 was assessed alongside that of free ATV and DTX in both 0.1 N HCl (pH 1.2) and PBS (pH 6.8), under conditions containing or lacking 2% (w/v) Tween 80. Under acidic conditions (pH 1.2), free ATV and DTX showed limited release, with cumulative values of 17.4% and 15.6% at 2 h, respectively. By contrast, MCT-NE#9 achieved 74.9% release of ATV and 44.9% of DTX at the same time point, indicating a marked improvement in dissolution (Figure S4A, B). Upon incorporation of 2% (w/v) Tween 80, the dissolution of free ATV and DTX rose to 50.9% and 68.6%, respectively. In contrast, MCT-NE#9 achieved over 90% release of both drugs within 2 hours, highlighting its markedly enhanced dissolution performance (Figure S4A, B).

In PBS (pH 6.8), free ATV and DTX exhibited cumulative release values of only 45.2% and 25.7% after 6 h, whereas MCT-NE#9 achieved 82.4% (ATV) and 68.1% (DTX) over the same period (Figure S4C, D). In the presence of 2% (w/v) Tween 80 to guarantee sink conditions, MCT-NE#9 discharged over 90% of both drugs within the first 2 hours and achieved full dissolution by 6 hours. By contrast, free ATV and DTX showed maximum release values of 66.1% and 74.1% after 6 hours (Figure S4C, D). Collectively, the results suggest that MCT-NE#9 substantially enhances the dissolution of both drugs across gastrointestinal pH conditions, particularly under surfactant-supplemented sink conditions.

The droplet size and PDI of MCT-NE#9 after dispersion in dissolution media were consistent with those measured in water: 10.9 ± 0.095 nm (PDI 0.195 ± 0.002) in pH 1.2 and 11.0 ± 0.056 nm (PDI 0.147 ± 0.033) in pH 6.8, compared with 12.9 ± 0.236 nm (PDI 0.264 ± 0.002) in water. After dissolution, the droplet size remained stable at 10.6 ± 0.233 nm (pH 1.2) and 11.3 ± 0.067 nm (pH 6.8). The zeta potential values were -3.15 ± 0.423 mV (pH 1.2) and -4.14 ± 0.334 mV (pH 6.8), comparable to that in water (-3.61 ± 0.695 mV), indicating that the formulation maintained uniform colloidal stability throughout the study.

During 1 month of storage, the contents of ATV and DTX in MCT-NE#9 remained stable, ranging from 96.5 ± 0.89% to 102 ± 2.26% and from 96.8 ± 0.94% to 102 ± 1.85%, respectively, with no significant differences observed between the two storage conditions (25 ± 2 °C/65 ± 5% RH and 40 ± 2 °C/75 ± 5% RH). The cumulative drug release at 2 h in 0.1 N HCl (pH 1.2) containing 2% (w/v) Tween 80 consistently exceeded 90% for both drugs (ATV: 91.7-94.1%; DTX: 96.9-99.6%), indicating that the formulation's dissolution performance was unaffected by storage. Moreover, the droplet size (13.0-15.5 nm), PDI (0.261-0.373), and zeta potential (-4.44 to -2.46 mV) of the reconstituted dispersions showed only minor variations compared with their initial values, confirming that MCT-NE#9 retained its physicochemical integrity and colloidal stability under both storage conditions (Table S5).

However, because this evaluation was limited to a 1-month period, additional long-term stability studies are warranted to verify the sustained preservation of drug content, dissolution characteristics, and nanoscale properties during extended storage.

### *In vivo* oral absorption of MCT-NE in rats

**Figure 3:**
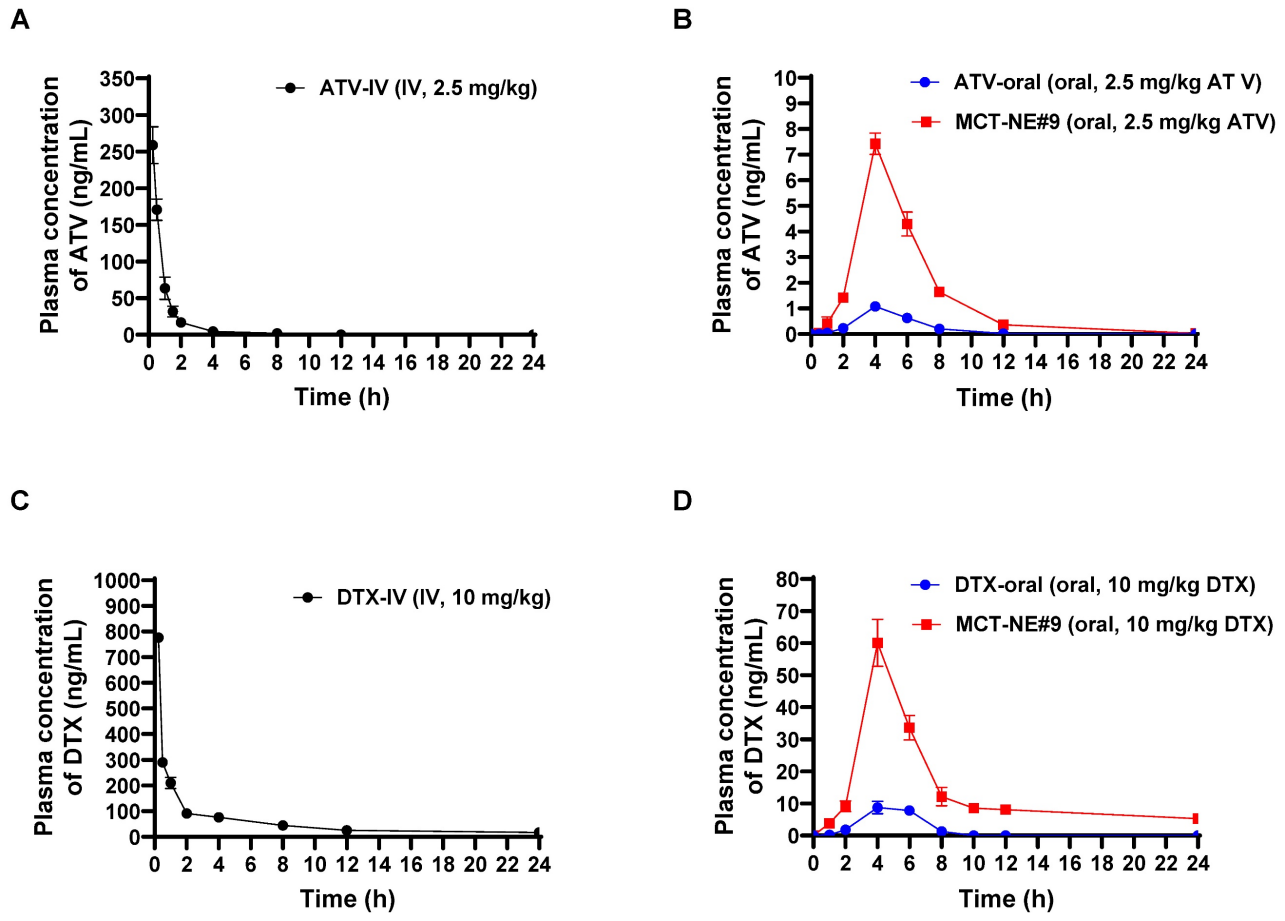
***In vivo* pharmacokinetic analysis of ATV/DTX.** Profiles of venous plasma concentrations of ATV and DTX in rats following (**A**) a single IV administration of 2.5 mg/kg ATV (ATV-IV), (**B**) oral administration of an aqueous dispersion of 2.5 mg/kg ATV (ATV-oral) or MCT-NE#9 (equivalent to 2.5 mg/kg of ATV), (**C**) a single IV administration of 10 mg/kg DTX (DTX-IV), and (**D**) oral administration of an aqueous dispersion of 10 mg/kg DTX (DTX-oral) or MCT-NE#9 (equivalent to 10 mg/kg of DTX). Values are shown as mean ± SD (n = 4).

The plasma concentration-time profiles and corresponding pharmacokinetic parameters after IV injection of free ATV or DTX, and oral administration of ATV and DTX as either ATV/DTX in water or MCT-NE#9 in rats, are shown in Figure 3 and Table S6. When contrasted with ATV/DTX administered as an aqueous dispersion, plasma drug levels were significantly elevated after incorporation into MCT-NE#9. In particular, compared with free oral ATV/DTX, the maximum plasma concentration (C_max_) and area under the plasma concentration-time curve (0-24 h) (AUC_last_) of ATV after oral administration of MCT-NE#9 were enhanced by 6.86- and 7.60-fold, respectively. Similarly, C_max_ and AUC_last_ for DTX after oral administration of MCT-NE#9 were 5.67- and 9.54-fold higher, respectively, than the corresponding parameters for free oral DTX. These improvements caused the oral bioavailabilities of ATV and DTX in MCT-NE#9 to be 659% and 851% greater, respectively, than the corresponding values for oral ATV/DTX dispersed in water (ATV: 1.66 ± 0.381% vs. 12.6 ± 0.753%; DTX: 2.24 ± 0.709% vs. 21.3 ± 3.82%).

The improved oral bioavailability observed for MCT-NE#9 aligns well with the findings from the *in vitro* permeability studies and the associated mechanistic evaluations. This improvement may be attributed to multiple factors, including multimodal transport mechanisms, inhibition of P-gp-mediated efflux by TPGS, ASBT- and SMVT-facilitated transport via DCK/Biotinyl PE in MCT-NE#9, as well as endocytosis and macropinocytosis of the NE systems.

### *In vitro* cytotoxicity of MCT-NEs

**Figure 4:**
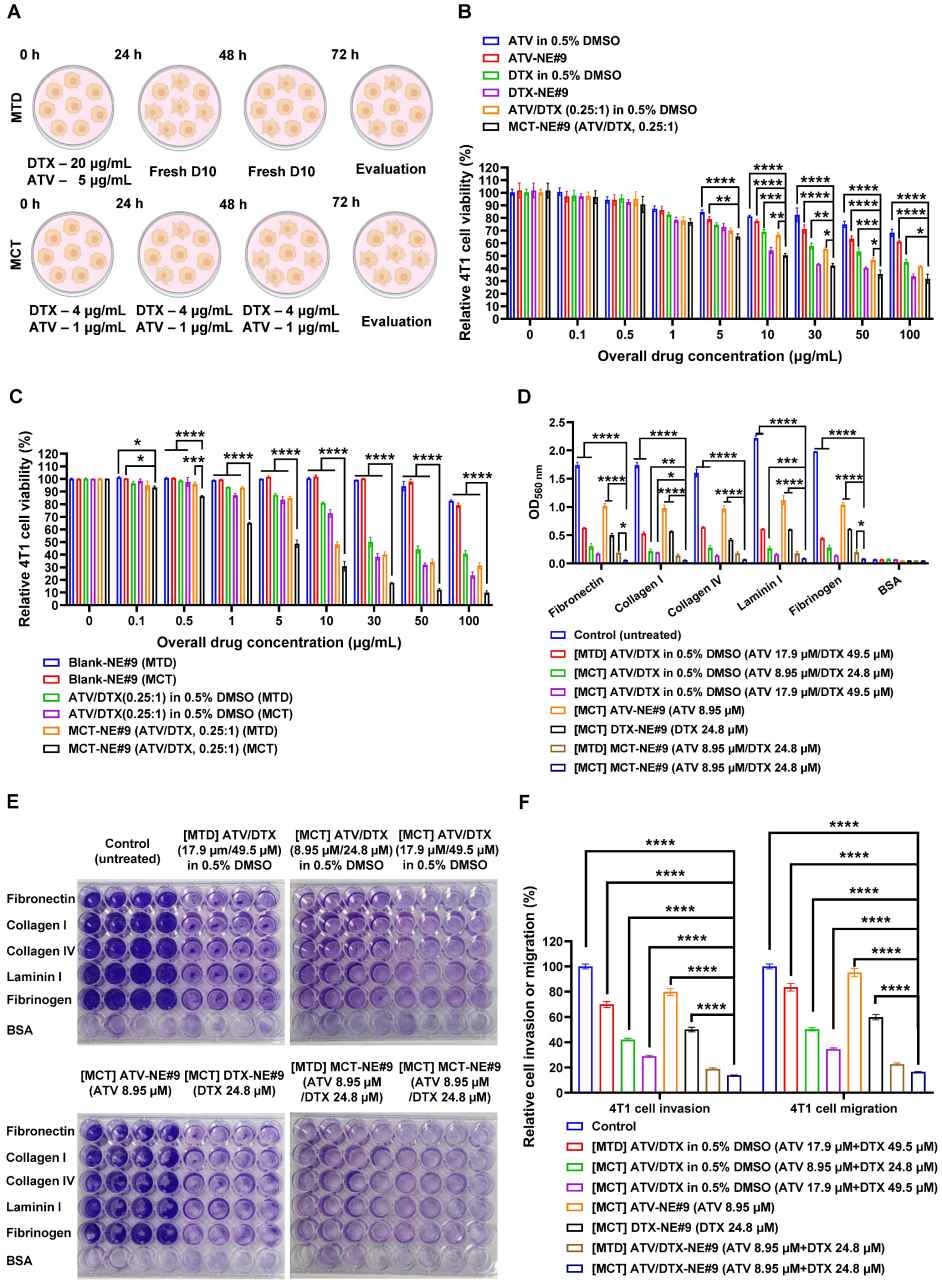
***In vitro* antitumor and antimetastatic activities of MCT-NEs.** (**A**) Schematic illustration of the cytotoxicity assessment of ATV and/or DTX in 4T1 cells under MTD or MCT regimens. (**B**) Combined effects of ATV/DTX delivered in 0.5% DMSO or MCT-NE#9 on 4T1 cells at different ATV-to-DTX weight ratios (mean ± SD, n = 4). (**C**) Cytotoxic responses of ATV/DTX in 0.5% DMSO or MCT-NE#9 following MTD or MCT schedules (mean ± SD, n = 4). (**D, E**) Quantification and representative images of 4T1 cell adhesion to ECM proteins (fibronectin, collagen I, collagen IV, laminin I, fibrinogen, and BSA) in 48-well plates (mean ± SD, n = 4). (**F**) Inhibitory effects of ATV/DTX in 0.5% DMSO or MCT-NE#9 on 4T1 cell migration and invasion under MTD or MCT treatment conditions (mean ± SD, n = 3). * p < 0.05, ** p < 0.01, *** p < 0.001, **** p < 0.0001.

The synergistic cytotoxicity of ATV and DTX against 4T1 cells was evaluated (Figure 4A-C). The *in vitro* cytotoxicity of ATV and DTX-loaded NE#9 against 4T1 cells showed dose-dependent effects at concentrations > 10 µg/mL. NE#9 formulations exhibited stronger anticancer activity than drugs in 0.5% DMSO, reducing cell viability more effectively. At 10 µg/mL, ATV-NE#9 and DTX-NE#9 reduced viability to 77.3% and 54.3%, respectively, versus 81.3% and 69.1% for free drugs, indicating enhanced solubility and cellular uptake (Figure 4B).

Conversely, ATV/DTX (0.25:1, w/w) in 0.5% DMSO showed greater cytotoxicity than either drug alone. MCT-NE#9 further enhanced anticancer effects, showing 1.53- and 1.08-fold higher efficacy than ATV-NE#9 and DTX-NE#9, respectively. Its IC_50_ (11.8 µg/mL) indicated significantly improved potency: 73.5%, 80.6%, and 45.8% higher than ATV/DTX in 0.5% DMSO, ATV-NE#9, and DTX-NE#9, respectively (Figure 4B).

Following confirmation of synergistic effects, MCT treatment further enhanced antitumor activity. At 10 µg/mL, cell viability dropped from 80.9% (MTD) to 72.9% (MCT) with ATV/DTX in 0.5% DMSO. MCT treatment of MCT-NE#9 reduced viability to 30.8%, a 1.56-fold decrease versus MTD treatment (Figure 4C). Its IC_50_ was 4.84 µg/mL, 10.2- and 2.15-fold lower than that of MTD-treated ATV/DTX and MCT-NE#9, respectively, indicating superior efficacy.

Overall, incorporation of ATV and DTX into an NE system significantly enhanced the anticancer efficacy of metronomic treatment. This approach, coupled with prolonged metronomic dosing, demonstrated superior performance relative to conventional treatment methods, substantially increasing the therapeutic impacts of ATV and DTX.

### *In vitro* cell migration, invasion, and adhesion assay of MCT-NE#9

The ability of MCT-NE#9 to inhibit metastatic behavior was examined using a series of *in vitro* assays evaluating adhesion, migration, and invasion in 4T1 cells. When cells were exposed to an MCT regimen of ATV/DTX (17.9 µM/49.5 µM) prepared in 0.5% DMSO, their attachment to ECM substrates—including fibronectin, collagen I and IV, laminin I, and fibrinogen—was markedly diminished relative to both the untreated control and the MTD-treated group (Figure 4D). Notably, MCT treatment with MCT-NE#9 (ATV 8.95 µM/DTX 24.8 µM) led to greater adhesion suppression than single drug-loaded NEs or MTD treatment, achieving up to 90.5% inhibition relative to MTD-treated ATV/DTX in 0.5% DMSO (Figure 4E).

Furthermore, MCT using ATV/DTX in 0.5% DMSO significantly reduced 4T1 cell invasion and migration by 57.9% and 49.7%, respectively, with 39.7% higher inhibition than MTD treatment. MCT with MCT-NE#9 further enhanced these effects, decreasing invasion and migration by 80.2% and 52.2%, respectively, relative to MTD and MCT treatments with ATV/DTX in 0.5% DMSO (Figure 4F). These superior antimetastatic effects are attributed to improved solubility, cellular permeability, and synergistic drug action. Compared to the MTD method, MCT with MCT-NE#9 showed 27.0% lower migration/invasion capacity, with overall reductions of 86.2% and 83.5%, respectively, versus controls. These findings suggest that MCT of MCT-NE#9 offers enhanced therapeutic efficacy against tumor progression and metastasis.

### MCT-NE#9 treatment of ATV/DTX enhances ferroptosis in 4T1 cells

**Figure 5:**
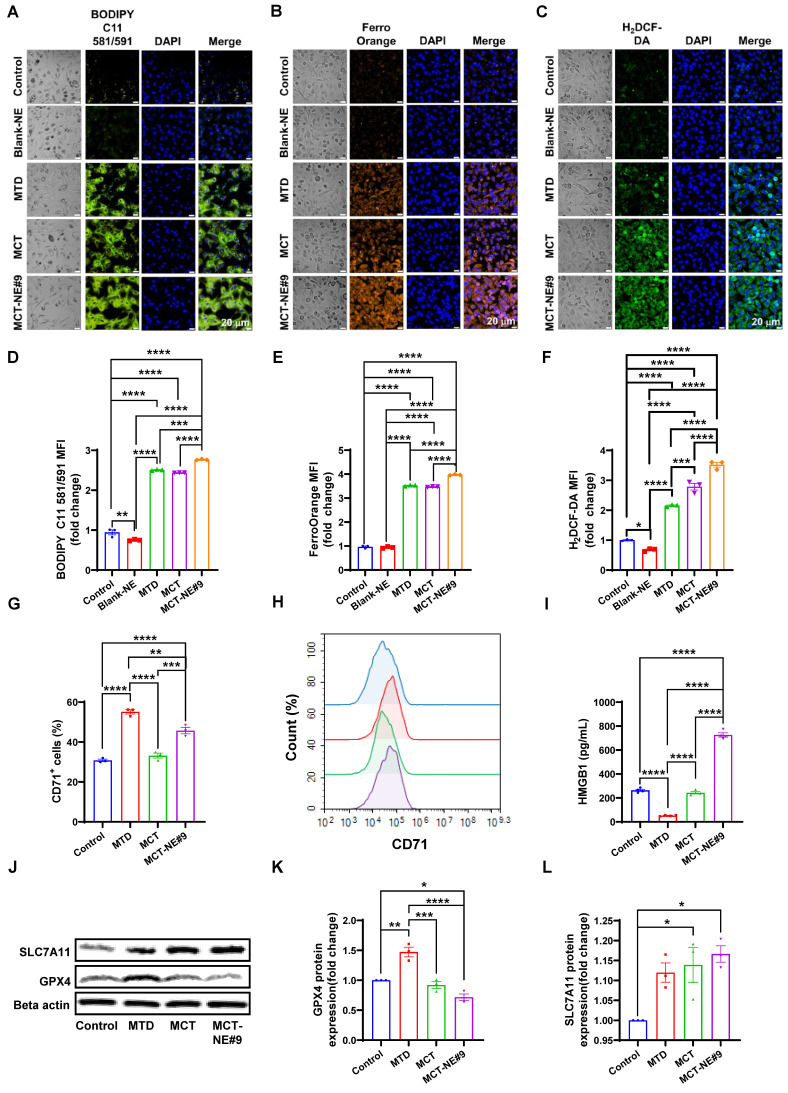
**MCT-NE#9 induces breast cancer ferroptosis.** Confocal laser scanning microscopy images of (**A**) LPO, (**B**) iron ion accumulation, and (**C**) ROS generation after treatment with blank-NE, MTD, MCT and MCT-NE#9. Scale bar: 20 μm. Flow cytometry of ferroptosis after treatment with blank-NE#9, MTD, MCT, and MCT-NE#9. (**D**) Quantification of the MFI of LPO. (**E**) Quantification of the MFI of iron ion accumulation. (**F**) Quantification of the MFI of ROS. Values are shown as mean ± SEM (n = 3 per group). (**G**) Quantification of the percentages of CD71 after treatment with MTD, MCT, and MCT-NE#9. Values are shown as mean ± SEM (n = 3 per group). (**H**) Histogram representation of CD71 expression levels, corresponding to the quantification shown in panel (G). (**I**) Quantification of HMGB1 levels measured by ELISA after treatment with MTD, MCT, and MCT-NE#9. Values are shown as mean ± SEM (n = 4 per group). (**J**) Representative western blot analysis of SLC7A11 and GPX4 protein expression in the control, MTD, MCT, and MCT-NE#9 groups. β-actin was used as a loading control. (**K**) Quantification of GPX4 protein expression (fold change) normalized to β-actin, corresponding to the western blot results in panel (J). Values are shown as mean ± SEM (n = 3 per group). (**L**) Quantification of SLC7A11 protein expression (fold change) normalized to β-actin, corresponding to the western blot results in panel (J). Values are shown as mean ± SEM (n = 3 per group). * p < 0.05, ** p < 0.01, *** p < 0.001, **** p < 0.0001.

To evaluate whether MCT-NE#9 induces ferroptosis in 4T1 cells, we assessed key markers including LPO, intracellular iron, and ROS. BODIPY C11 staining revealed 2.51-, 2.44-, and 2.77-fold increases in mean fluorescence intensity (MFI) for the MTD group (single treatment with free ATV 5 µg/mL + DTX 20 µg/mL), the MCT group (repeated treatment with the free ATV 1 µg/mL + DTX 4 µg/mL every 24 h for 72 h), and the MCT-NE#9 group (NE formulation containing ATV 1 µg/mL + DTX 4 µg/mL, administered once daily for 72 h), respectively, compared to the untreated control. Notably, MCT-NE#9 treatment resulted in significantly higher LPO than either MTD or MCT (Figure 5A, D). In contrast, the blank-NE group showed a 1.27-fold decrease in MFI relative to control, indicating no LPO induction in the absence of active drugs.

FerroOrange staining showed increased intracellular iron accumulation by 3.53-, 3.47-, and 3.97-fold in the MTD, MCT, and MCT-NE#9 groups, respectively, with MCT-NE#9 again showing the highest accumulation (Figure 5B, E). ROS levels measured via H_2_DCF-DA staining were also significantly elevated: 2.16-, 2.79-, and 3.52-fold increases in MTD, MCT, and MCT-NE#9 groups, respectively (Figure 5C, F).

Surface expression of the ferroptosis marker CD71 was elevated by 1.79- (MTD) and 1.48-fold (MCT-NE#9), while MCT alone showed no change (Figure 5G, H). HMGB1 release, a DAMP associated with ferroptosis-induced cell death, decreased 5.05-fold in the MTD group, showed no significant change with MCT, but increased 2.75-fold in the MCT-NE#9 group (Figure 5I).

Western blot analysis revealed that GPX4 expression increased 1.47-fold with MTD, but decreased to 1.39-fold with MCT-NE#9, indicating ferroptosis induction (Figure 5J, K). SLC7A11 expression increased slightly in both the MCT (1.14-fold) and MCT-NE#9 (1.17-fold) groups (Figure 5J, L).

In summary, MCT-NE#9 most effectively induced ferroptosis in 4T1 cells, as evidenced by elevated LPO, iron accumulation, ROS generation, and downregulation of GPX4. These effects were likely due to sustained intracellular drug levels and enhanced permeability enabled by the NE formulation.

### MCT-NE#9 enhances ability of macrophages to recognize and phagocytose 4T1 cells

**Figure 6:**
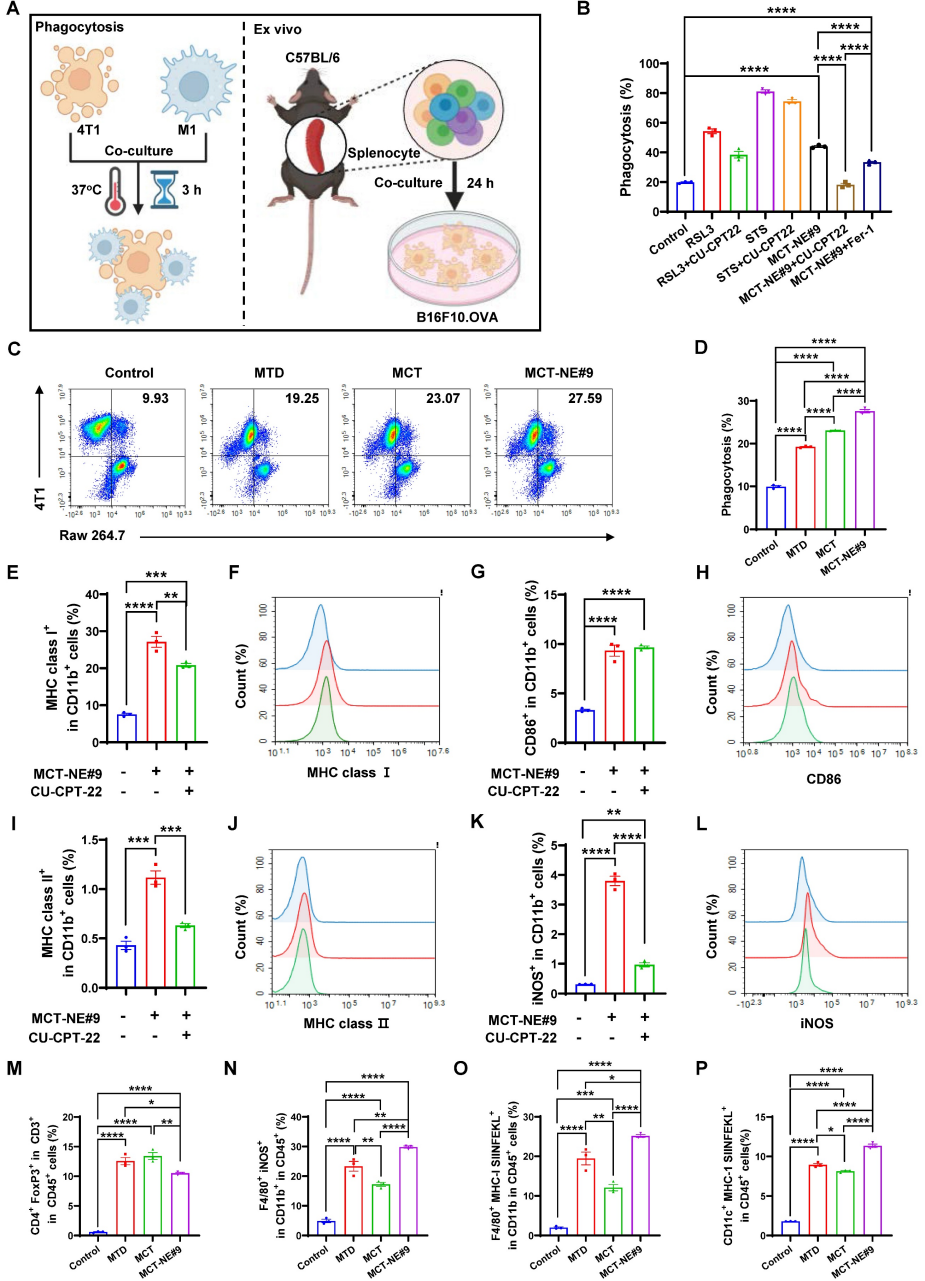
***In vitro* phagocytosis and *ex vivo* analysis of immune cell activation.** (**A**) Schematic representation of the experimental setup. (**B**) Quantification of the phagocytosis rate (%) of macrophages after treatment with RSL3, STS, MCT-NE#9, and CU-CPT22, analyzed by flow cytometry. Values are shown as mean ± SEM (n = 3 per group). (**C**) Representative flow cytometry dot plots showing phagocytosis of 4T1 cells by Raw 264.7 macrophages under different treatment conditions. (**D**) Quantification of the phagocytosis rate (%) of Raw 264.7 macrophages following different treatments. (**E, F**) Quantification and histogram representation of MHC class I expression on CD11b^+^ cells after MCT-NE#9 and CU-CPT22 treatment. (**G, H**) Quantification and histogram representation of CD86 expression on CD11b^+^ cells after MCT-NE#9 and CU-CPT22 treatment. (**I, J**) Quantification and histogram representation of MHC II class expression on CD11b^+^ cells after MCT-NE#9 and CU-CPT22 treatment. (**K, L**) Quantification and histogram representation of iNOS expression on CD11b^+^ cells after MCT-NE#9 and CU-CPT22 treatment. (**M**) Quantification of T_regs_ in the splenocyte co-culture system after treatment with MTD, MCT, and MCT-NE#9. (**N**) Quantification of M1 macrophages in the splenocyte co-culture system after treatment with MTD, MCT, and MCT-NE#9. (**O**) Quantification of macrophage-mediated antigen presentation in the splenocyte co-culture system after treatment with MTD, MCT, and MCT-NE#9. (**P**) Quantification of dendritic cell-mediated antigen presentation in the splenocyte co-culture system after treatment with MTD, MCT, and MCT-NE#9. Values are shown as mean ± SEM (n = 3). * p < 0.05, ** p < 0.01, *** p < 0.001, **** p < 0.0001.

To assess phagocytosis efficiency under MCT and MTD treatment conditions, we evaluated TLR2-mediated responses using a phagocytosis assay (Figure 6A). Ferroptosis inducer (RSL3) and apoptosis inducer (STS) served as controls. Co-culture of drug-treated 4T1 cells with Raw264.7 macrophages revealed that the TLR2 antagonist CU-CPT-22 selectively suppressed phagocytosis in the RSL3-treated group, but not in STS-treated cells, indicating that ferroptosis-triggered phagocytosis is TLR2-dependent. In MCT-NE#9-treated cells, co-treatment with CU-CPT-22 or the ferroptosis inhibitor Fer-1 reduced phagocytosis by 2.41- and 1.32-fold, respectively (Figure 6B, S5).

Phagocytosis was enhanced more by MCT treatment than by MTD: MTD increased macrophage uptake by 1.94-fold, whereas MCT resulted in a 2.32-fold increase. MCT-NE#9 showed the greatest enhancement, with a 2.78-fold increase in phagocytosis relative to the control (Figure 6C, D). These results suggested that MCT-NE#9 enhances ferroptosis and subsequent phagocytosis.

Following 24 h co-culture, MCT-NE#9 increased macrophage MHC class I expression by 3.61-fold, CD86 by 2.83-fold, MHC class II by 2.60-fold, and iNOS by 12.3-fold compared to the control. Co-treatment with CU-CPT-22 reduced these increases to 1.30-fold for MHC class I, 1.78-fold for MHC class II, and 3.92-fold for iNOS (Figure 6E-L). These effects were not due to direct CU-CPT-22 stimulation, as it independently increased MHC class I, MHC class II, and CD86, but not iNOS (Figure S6), confirming that MCT-NE#9 activates macrophages through TLR2-dependent mechanisms.

Next, we analyzed whether ferroptosis induced by MCT-NE#9 could enhance the antigen-presenting ability of antigen-presenting cells and the activation of other immune cells using flow cytometry (Figure 6A, M-P, S7). In B16F10.OVA-splenocyte co-cultures, MCT-NE#9 slightly reduced T_reg_ populations by 1.20-fold compared to MTD, with no significant difference versus MCT (Figures 6M, S7A). CD4⁺, CD8⁺ T cells, NK, and NKT cells all increased across treatment groups, with no major differences among MTD, MCT, and MCT-NE#9 (Figure S8, S9).

Among the total immune cells, myeloid lineage cells significantly increased in all treatment groups, showing 3.88-fold, 4.32-fold, and 4.53-fold increases in the MTD, MCT, and MCT-NE#9 groups, respectively, compared to the control (Figure S10A, B). Among CD11b⁺ splenocytes, M1 macrophage levels increased in all treated groups, with MCT-NE#9 showing a 1.28- and 1.73-fold increase compared to MTD and MCT, respectively (Figure 6N, S7B). CD86 expression on M1 macrophages increased by 1.21-fold in the MCT-NE#9 group versus MTD (Figure S10C, E). Antigen-presenting capacity, measured by CD11b⁺ cells, was elevated in all treatment groups, with MCT-NE#9 exhibiting a 1.29- and 2.08-fold increase compared to MTD and MCT, respectively (Figure 6O, S7C). M2 macrophages increased modestly in all groups, without significant differences (Figure S10D, F). The antigen-presenting capacity of dendritic cells was increased in all treatment groups, with MCT-NE#9 showing a 1.26- and 1.39-fold enhancement compared to MTD and MCT, respectively (Figure 6P, S7D). Both the distribution and activation of dendritic cells were increased in all treatment groups compared to the control. Specifically, the distribution of dendritic cells in the MCT-NE#9 group increased by 1.22- and 1.36-fold compared to the MTD and MCT groups, respectively, while dendritic cell activation increased by 1.18-fold relative to both MTD and MCT (Figure S11).

### Biodistribution and safety evaluation of MCT-NE#9

**Figure 7:**
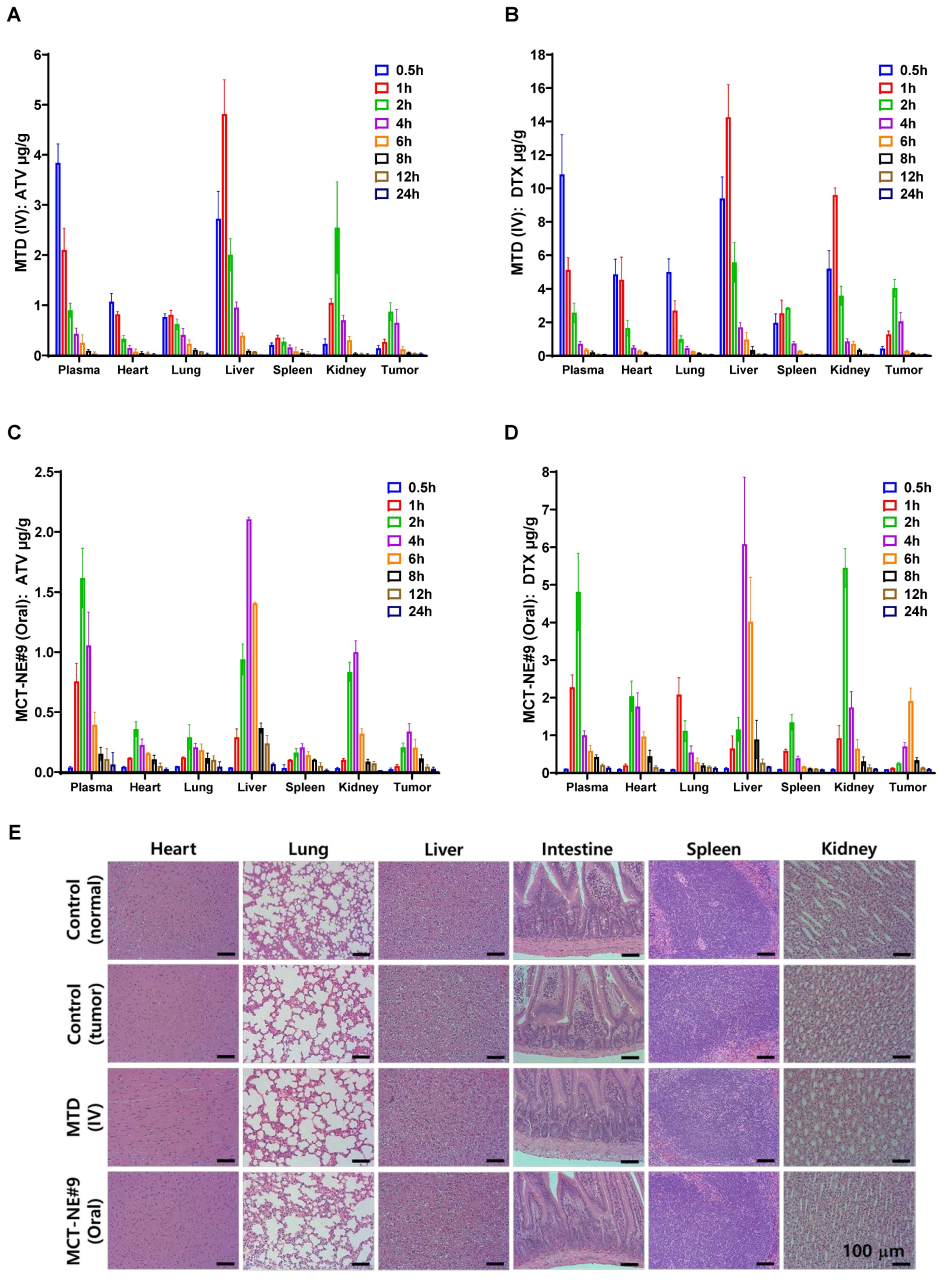
***In vivo* biodistribution and histological evaluation of MCT-NE#9.** (**A**-**D**) Plasma and tissue distribution of ATV and DTX in 4T1 tumor-bearing mice after IV administration of MTD (ATV 2.5 mg/kg + DTX 10 mg/kg, once every 3 weeks) and once-daily oral administration of MCT-NE#9 (ATV 2.5 mg/kg + DTX 10 mg/kg) for 21 days. (**E**) Representative hematoxylin and eosin (H&E)-stained sections of heart, lungs, liver, intestine, spleen, and kidneys from control, MTD (IV), and MCT-NE#9 (Oral) groups after 4 weeks of repeated administration. Scale bar: 100 µm.

Following administration of MTD (IV, once every 3 weeks) and MCT-NE#9 (oral, once daily), distinct differences in the pharmacokinetic and biodistribution profiles of ATV and DTX were observed in 4T1 tumor-bearing mice. In the IV group, the plasma concentrations of both drugs rose sharply to very high levels immediately after injection (ATV: 3.84 ± 0.378 µg/g; DTX: 10.8 ± 2.37 µg/g at 0.5 h), but declined rapidly, becoming nearly undetectable within 12 h. Tumor concentrations also peaked transiently (ATV: 0.873 ± 0.176 µg/g; DTX: 4.04 ± 0.519 µg/g at 2 h), followed by a rapid clearance, resulting in negligible levels well before the next injection at 3 weeks (Figure 7A, B). This intermittent exposure pattern suggests that, although an initial cytotoxic effect is achieved, residual tumor cells may proliferate during the drug-free intervals.

By contrast, daily oral administration of MCT-NE#9 produced lower peak plasma concentrations (ATV: 1.62 ± 0.248 µg/g; DTX: 4.81 ± 1.03 µg/g at 2 h) compared with IV dosing, but maintained measurable drug levels for up to 24 h (ATV: 0.062 ± 0.101 µg/g; DTX: 0.137 ± 0.027 µg/g). Tumor concentrations were also lower than those in the IV group at their respective peaks (ATV: 0.337 ± 0.066 µg/g at 4 h; DTX: 1.91 ± 0.345 µg/g at 6 h), yet remained detectable for up to 24 h after dosing (Figure 7C, D). Repeated daily dosing ensured continuous systemic and intratumoral exposure, consistent with a metronomic profile that exerts sustained cytotoxic pressure and reduces the likelihood of tumor regrowth between treatment cycles.

The drug distribution in metabolic and excretory organs also showed marked differences. In the IV group, both ATV and DTX accumulated rapidly in the liver and kidneys (liver: ATV 4.81 ± 0.684 µg/g, DTX 14.3 ± 1.94 µg/g at 1 h; kidneys: ATV 2.55 ± 0.913 µg/g at 2 h, DTX 9.60 ± 0.421 µg/g at 1 h), resulting in transient but substantial organ burden (Figure 7A, B). By contrast, the oral MCT-NE#9 group exhibited significantly lower hepatic and renal concentrations (liver: ATV 2.11 ± 0.017 µg/g, DTX 6.08 ± 1.78 µg/g at 4 h; kidneys: ATV 1.00 ± 0.095 µg/g at 4 h, DTX 5.45 ± 0.515 µg/g at 2 h), which declined gradually over time, indicating a reduced risk of acute organ toxicity (Figure 7C, D).

These distributional patterns were consistent with the systemic toxicity profiles. After 4 weeks of treatment, serum biochemistry showed clear hepatocellular injury in the MTD (IV) group, with AST 223 ± 73.7 U/L vs. 93.0 ± 19.3 U/L and ALT 52.5 ± 11.5 U/L vs. 31.2 ± 7.19 U/L (tumor control), together with moderate renal stress (BUN 26.6 ± 1.90 mg/dL vs. 20.0 ± 2.80 mg/dL; CRE 0.331 ± 0.021 mg/dL vs. 0.287 ± 0.028 mg/dL). ALP did not increase (151 ± 28.9 U/L vs. 140 ± 19.1 U/L), and TBIL remained low (0.006 ± 0.013 mg/dL), indicating hepatocellular rather than cholestatic injury (Figure S12). In contrast, the MCT-NE#9 (oral) group maintained all markers within physiological ranges (AST 92.2 ± 15.2 U/L; ALT 40.2 ± 6.30 U/L; BUN 22.5 ± 1.45 mg/dL; CRE 0.279 ± 0.016 mg/dL; ALP 150 ± 24.6 U/L; TBIL 0.004 ± 0.003 mg/dL), with electrolytes stable (PHOS 7.38 ± 0.335 mg/dL; Ca 9.46 ± 0.167 mg/dL), confirming the absence of systemic toxicity (Figure S12). Histopathology corroborated these trends, showing mild hepatocellular vacuolization with limited periportal inflammation, focal villous blunting in the ileum, and slight tubular epithelial swelling in the MTD (IV) group, whereas the oral MCT-NE#9 group displayed normal tissue architecture in the liver, kidney, intestine, spleen, heart, and lung without discernible pathological alterations (Figure 7E).

Collectively, these results demonstrate that IV administration results in transient high-concentration exposure with rapid clearance and acute organ burden, thereby creating drug-free intervals that may allow tumor regrowth. By contrast, daily oral administration of MCT-NE#9 produces lower but sustained systemic and tumor concentrations, reduces accumulation in metabolic and excretory organs, and minimizes systemic toxicity. This sustained exposure pattern underscores the therapeutic advantage of metronomic oral dosing over conventional high-dose intermittent IV administration.

### Metronomic treatment of MCT-NE#9 enhanced *in vivo* anti-cancer effects via induction of ferroptosis in the 4T1 mouse model

**Figure 8:**
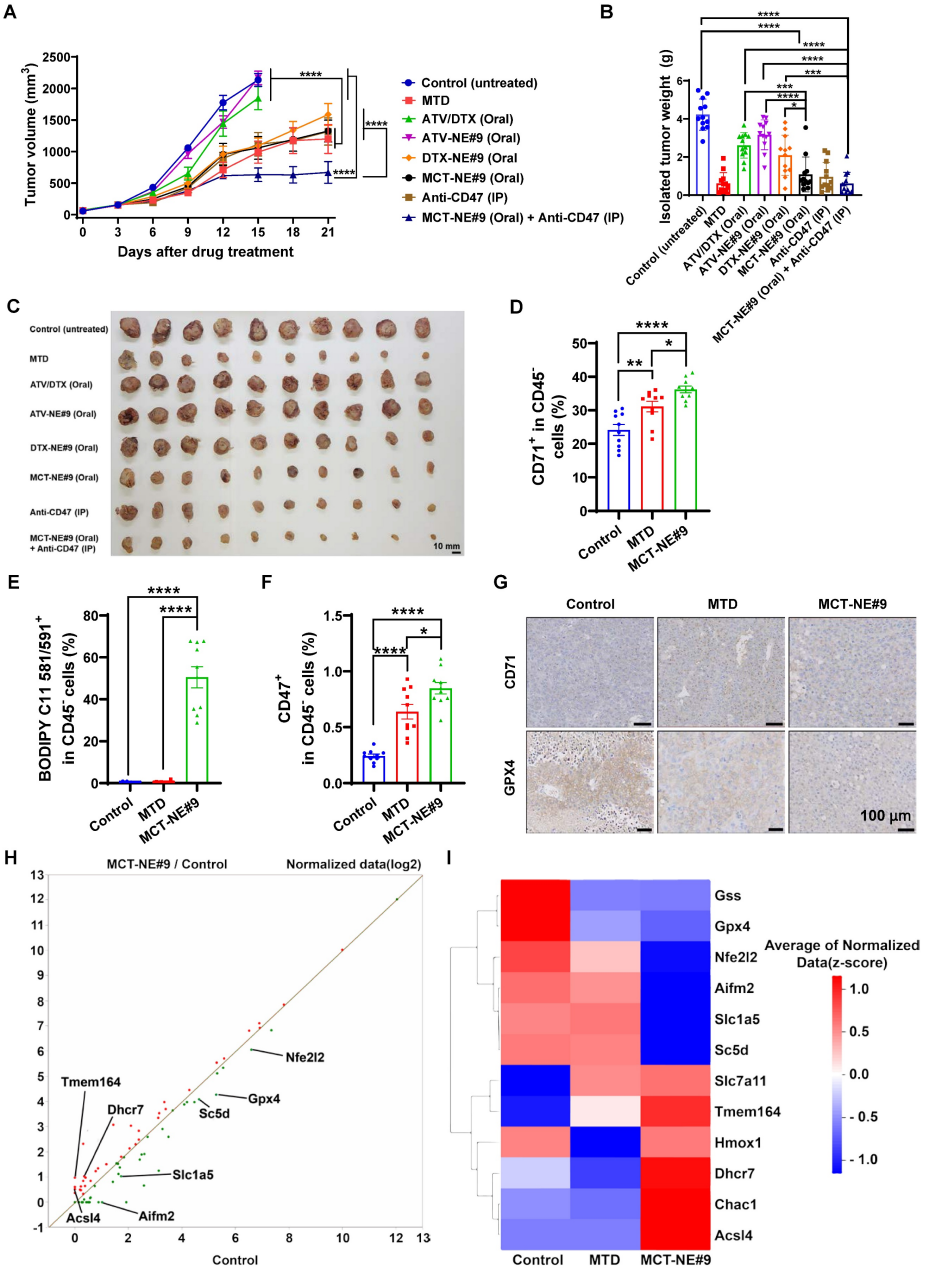
***In vivo* antitumor effects of MCT-NE#9.** (**A**) Tumor volume of each mouse for 21 days of treatment. Values are expressed as mean ± SEM (n = 10 per group). (**B**) Isolated tumor weight after 21 days. Values are expressed as mean ± SEM (n = 10 per group). (**C**) Photographs of tumors isolated from each group on day 21. Scale bar: 10 mm. (**D**) Quantification of CD71 in cancer cells. (**E**) Quantification of LPO in cancer cells. (**F**) Quantification of CD47 in cancer cells. (**G**) Immunohistochemical staining of CD71 and GPX4 in tumor tissues from control, MTD, and MCT-NE#9 groups. Values are shown as mean ± SEM (n = 10). (**H**) Scatter plot of ferroptosis-related gene expression in MCT-NE#9 versus control. Key markers (*Tmem164*, *Dhcr7*, *SLC7A11*, *Sc5d*, and *GPX4*) are highlighted. (**I**) Heatmap showing ferroptosis-related gene expression across control, MTD, and MCT-NE#9 groups. Red indicates upregulation, and blue indicates downregulation (n = 3). * p < 0.05, ** p < 0.01, *** p < 0.001, **** p < 0.0001.

The efficacy of orally administered MCT-NE#9 in tumor inhibition and modulation of ferroptosis pathway markers was evaluated in a murine 4T1 breast cancer model (Figure 8). Tumor growth control was observed from day 6, with the most significant differences noted on day 15. The untreated control group exhibited rapid tumor progression, reaching 2135 ± 103 mm^3^. IV-administered ATV/DTX (2.5/10 mg/kg) (MTD) reduced the tumor volume by 53.6%, whereas oral ATV/DTX (2.5/10 mg/kg) in 0.5% DMSO achieved only a 13.5% reduction. Among the NEs, ATV-NE#9 (2.5 mg/kg) exhibited no significant antitumor activity, while DTX-NE#9 (10 mg/kg) improved inhibition by 48.9%, likely due to the enhanced solubility and bioavailability of DTX. Oral MCT-NE#9, administered daily at a low dose, produced comparable efficacy to IV treatment (50.4% inhibition by day 15) and further surpassed DTX-NE#9 by 16.5% at day 21. Anti-CD47 monotherapy (IP) resulted in 47.8% suppression, whereas its combination with oral MCT-NE#9 achieved the greatest inhibition (70.3%), exceeding all single-agent groups (Figure 8A, S13). The stable tumor volume from days 15 to 21 implies a synergistic chemo-immunotherapy effect. Tumor weights were significantly lower (up to 90.6%) in mice receiving this combination than in all other treatment groups (Figure 8B, C). Moreover, all mice receiving metronomic oral MCT-NE#9 maintained stable body weight and exhibited a 100% survival rate up to day 21, confirming the absence of systemic toxicity. (Figure S14). These results indicate that pairing the formulation with immunotherapy may yield enhanced antitumor activity while maintaining a favorable safety profile. Nonetheless, additional studies examining long-term toxicity are still needed.

To confirm the induction of tumor ferroptosis *in vivo*, cancer cells were isolated from tumor tissue, stained for ferroptosis markers, and analyzed via flow cytometry. CD71 expression increased by approximately 1.29-fold in the MTD group and 1.50-fold in the MCT-NE#9 group compared to the control (Figure 8D, S15A). LPO levels showed no significant difference between the control and MTD groups but were approximately 140- and 112-fold higher in the MCT-NE#9 group compared to the control and MTD groups, respectively (Figure 8E, S15B).

Taken together, the data indicate that the metronomic dosing regimen promotes more robust and prolonged ferroptosis in the mouse tumor model, aligning with trends observed in the *in vitro* experiments.

We also evaluated the expression of CD47, known as the “don't eat me” signal. CD47 levels increased by approximately 2.67-fold in the MTD group and by 3.54-fold in the MCT-NE#9 group compared with the control (Figure 8F, S15C); however, this increase was not observed *in vitro* (Figure S16). To validate ferroptosis induction in tumors further, we analyzed CD71 and GPX4 expression via immunohistochemistry. CD71 expression was elevated in both the MTD and MCT-NE#9 groups compared with the control, whereas GPX4 expression was reduced in these groups (Figure 8G). These findings align with previous *in vitro* results.

We analyzed the mRNA expression levels of cancer cells to determine whether MCT-NE#9 and MTD treatments directly induce ferroptosis within tumors. Our results reveal that the expression levels of ferroptosis-inhibitory genes, including *GSS*, *GPX4*, and *Nfe2l2*, were lowest in the MCT-NE#9 group. Conversely, ferroptosis-promoting genes, such as *Acsl4*, *Hmox1*, and *CHAC1*, exhibited the highest expression in the MCT-NE#9 group. These findings imply that MCT-NE#9 induces sustained ferroptosis in tumors more effectively than does MTD, likely due to prolonged drug retention in the plasma. However, some genes displayed contradictory patterns regarding ferroptosis induction, highlighting the complexity of ferroptosis-related pathways (Figure 8H, I, S17).

Furthermore, when comparing genes involved in biological processes, we observed a reduction in the expression of genes associated with the oxidative stress response, negative regulation of ferroptosis, and cellular responses to oxidative stress (Figure S18). To determine whether cell death was definitively induced, we also analyzed apoptosis-related gene expression. The results show that apoptosis-inducing genes, including *Casp8*,* Casp9*,* Fas*,* Diablo*,* Smad2*, and *Smad3*, were most highly expressed in the MCT-NE group, while apoptosis-inhibitory genes, such as *Bcl2* and *Xiap*, exhibited the lowest expression. These findings imply that MCT-NE#9 not only induces ferroptosis but also promotes apoptosis (Figure S19).

### Metronomic oral administration of MCT-NE#9 effectively improves the TME and enhances immune responses by maintaining ferroptosis

**Figure 9:**
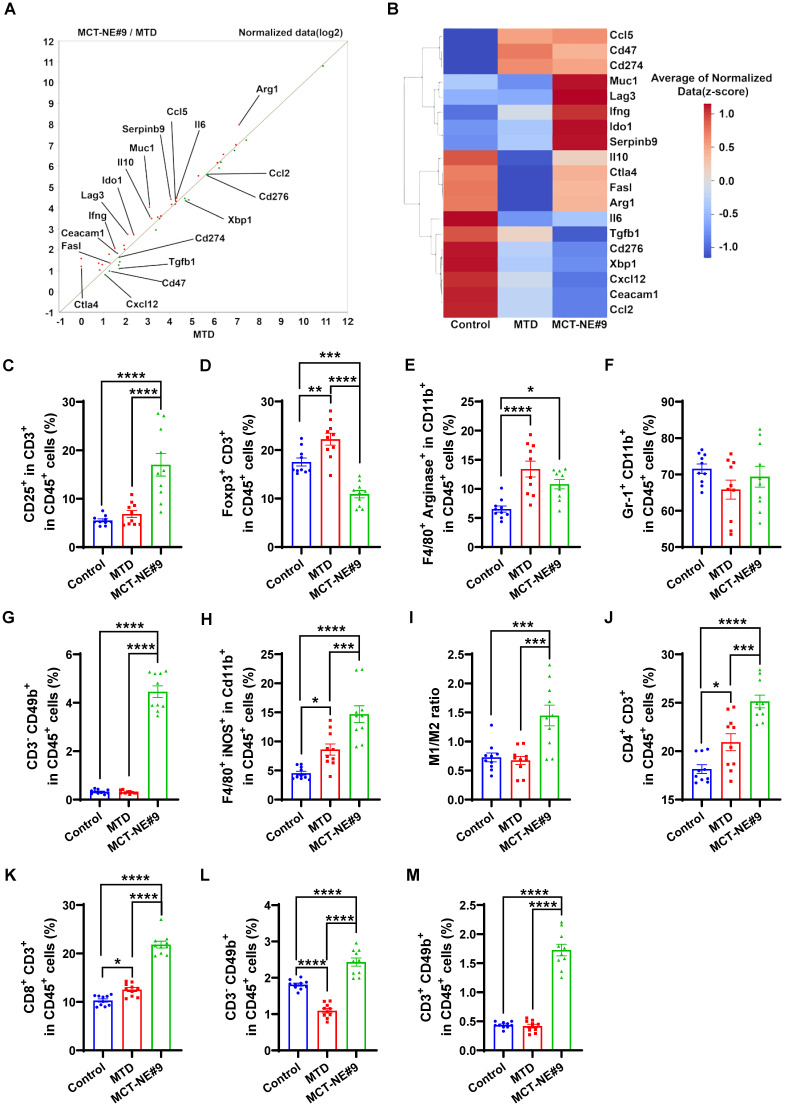
***In vivo* immune analysis in tumor and lymph nodes.** (**A**) Scatter plot of immune response-related gene expression in tumor cancer cells (MCT-NE#9—oral administration ATV 2.5 mg/kg + DTX 10 mg/kg once daily vs. MTD—IV administration of ATV 2.5 mg/kg + DTX 10 mg/kg once every 3 weeks). Key genes (*Arg1*, *CD274*, and *Ifng*) involved in immune regulation are highlighted (n = 3). (**B**) Heatmap showing the expression patterns of immune-related genes across control, MTD, and MCT-NE#9 groups. Red indicates upregulation and blue indicates downregulation of key immune markers (n = 3). Flow cytometry analysis of immune cell populations in tumor tissues and TDLNs. (**C**) Quantification of T-cell activation in the tumor. (**D**) Quantification of T_regs_ in the tumor. (**E**) Quantification of M2 macrophages in the tumor. (**F**) Quantification of MDSCs in the tumor. (**G**) Quantification of NK cells in the tumor. (**H**) Quantification of M1 macrophages in the tumor. (**I**) M1/M2 macrophage ratio in the tumor. (**J**) Quantification of CD4 T cells in TDLNs. (**K**) Quantification of CD8 T cells in TDLNs. (**L**) Quantification of NK cells in TDLNs. (**M**) Quantification of NKT cells in TDLNs. Data are presented as the mean ± SEM (n = 10). * p < 0.05, ** p < 0.01, *** p < 0.001, **** p < 0.0001.

To assess how sustained ferroptosis induced by MCT-NE#9 treatment affects immune cells within the TME, we analyzed immune-related mRNA expression. Results showed increased expression of antitumor immune genes like *CCl5* and *IFNγ*, alongside upregulated immune-suppressive genes such as *CD47*, *CD274* (PD-L1), *Muc1*, and *Ido1*. In contrast, genes that suppress antitumor immunity (*TGFβ1*, *Arg1*, *IL6*, *CXCL12*, and *CD276*) were downregulated. This suggested that MCT-NE#9 treatment enhances immune responses within the TME (Figure 9A, B).

Flow cytometry was used to investigate ferroptosis and immune cell changes. CD25 expression, indicative of T-cell activation, increased by 3.08- and 2.49-fold in MCT-NE#9 compared to control and MTD, respectively (Figure 9C, S20A). T_regs_ increased by 1.27-fold in MTD, but notably decreased by 1.61-fold in MCT-NE#9 (Figure 9D, S20B). M2 macrophages increased by 2.05-fold in MTD, while MCT-NE#9 reduced them by 1.24-fold (Figure 9E, S20C). MDSCs showed no significant changes (Figure 9F, S20D). NK cells increased by 13.5- and 14.3-fold in MCT-NE#9 compared to control and MTD, respectively (Figure 9G, S21A). M1 macrophages increased by 1.90-fold in MTD and by an additional 1.71-fold with MCT-NE#9 (Figure 9H, S21B). The M1/M2 macrophage ratio doubled in the MCT-NE#9 group (Figure 9I).

Immune cells in lymph nodes near the tumor were also analyzed. CD4 T cells increased by 1.15-fold in MTD and by 1.20-fold in MCT-NE#9 compared to MTD (Figure 9J, S22A). CD8 T cells increased by 1.22-fold in MTD and by an additional 1.74-fold in MCT-NE#9 (Figure 9K, S22B). NK cells decreased by 1.75-fold in MTD, but increased by 2.23-fold in MCT-NE#9 (Figure 9L, S22C). NKT cells increased by 3.93- and 4.12-fold in MCT-NE#9 compared to control and MTD, respectively (Figure 9M, S22C).

In the blood systemic immune response, CD11b^+^ cell levels showed no significant differences (Figure S23A, S24A). Granulocytes increased by 1.10-fold in MCT-NE#9 compared to control, and by 1.13-fold compared to MTD (Figure S23B, S24B).

Monocytes increased by 1.42-fold in MCT-NE#9 compared to control, and 1.67-fold compared to MTD (Figure S23C, S24B).

T cells in the blood showed slight changes. Total T-cell levels decreased by 1.05-fold in MTD, and 1.43-fold in MCT-NE#9 (Figure S23D, S25A). There was no difference in CD4 T cell levels among the groups (Figure S23E, S25B). CD8 T cell levels decreased by 1.19-fold in MCT-NE#9 compared to control (Figure S23F, S25C).

## Discussion

Overcoming therapeutic resistance in breast cancer, particularly in TNBC, remains a major clinical challenge 45. Although recent advances in targeted therapies and immunotherapies have provided new treatment options, their efficacy remains inconsistent, and conventional chemotherapy still causes significant systemic toxicity 46. In this context, the metronomic approach has gained attention as a viable strategy to enhance treatment efficacy while minimizing therapy-related toxicity 47,48. This study investigated an orally administered MCT strategy co-delivering DTX and ATV to sustain ferroptosis and overcome the limitations of current cancer therapies.

To enhance the oral delivery of DTX and ATV, we developed a ligand-directed nanocarrier (MCT-NE) incorporating bile acid and biotinylated lipids. This design resulted in markedly improved drug solubility, permeability, and bioavailability via a triple absorption enhancement mechanism. The dual-ligand system—consisting of deoxycholic acid-conjugated lysine (DCK) and Biotinyl PE—was engineered to simultaneously target ASBT and SMVT transporters, enabling complementary and sustained transcellular absorption across the intestinal epithelium. The MCT-NE system also includes surfactants known to inhibit efflux transporters. In mechanistic studies using Caco-2/HT29-MTX monolayers, MCT-NE#9 showed significantly reduced permeability in the presence of ASBT and SMVT inhibitors, confirming transporter-mediated uptake. Nonetheless, blocking P-gp did not further alter permeation, suggesting that the formulation inherently minimized P-gp-driven efflux activity.

This multivalent transporter targeting and intrinsic efflux blockade facilitated prolonged systemic exposure and sustained plasma drug concentrations, which are essential for successful metronomic ferroptosis induction. Indeed, MCT-NE#9 induced ferroptosis more potently than conventional MTD treatment, as demonstrated by higher levels of LPO, intracellular iron accumulation, ROS, and HMGB1 release. While the expression of CD71—a marker of transferrin-mediated iron uptake—was highest in the MTD group, iron accumulation peaked in the MCT-NE#9 group, suggesting alternative mechanisms may contribute to iron influx or retention during ferroptosis.

Analysis of ferroptosis-related proteins further supported this conclusion: GPX4, a key suppressor of LPO, was most strongly downregulated in the MCT-NE#9 group, indicating enhanced ferroptotic vulnerability, while the expression of another inhibitory, SLC7A11, was highest in the same group. Under MCT-NE#9 treatment, we observed compensatory SLC7A11 upregulation alongside persistent GPX4 downregulation. We interpret this as a compensation bottleneck: stress-response pathways such as ATF4 and NRF2 transcriptionally induce SLC7A11 to enhance cystine import, yet the terminal lipid-ROS detoxification axis (GPX4/CoQ10) remains restricted 49,50. This limitation is likely linked to suppression of the mevalonate pathway, compromising both the synthesis of CoQ10 (an FSP1-dependent radical-trapping antioxidant) and the selenoprotein maturation of GPX4. The effect may be further reinforced by attenuated mTORC1-4EBP-dependent translation of GPX4 51-53. Consequently, lipid-ROS clearance becomes insufficient, and ferroptosis proceeds, consistent with our findings of elevated LPO, iron, ROS, and HMGB1 release during ferroptotic death 54. Although the CoQ10 content and mTORC1 activity were not quantified in this study, the proposed compensation bottleneck model could be validated in future experiments using CoQ10 or selenium supplementation and phosphorylation assays of S6K and 4EBP1.

Macrophage TLR2 played a crucial role in recognizing ferroptotic cells. Co-culturing 4T1 cells treated with RSL3, STS, or MCT-NE#9 with TLR2-inhibited macrophages resulted in reduced phagocytosis, confirming that TLR2 recognizes ferroptotic cells. To evaluate how MCT-NE#9-induced phagocytosis modulates the immune system, we co-cultured B16F10.OVA cells with splenocytes. B16F10.OVA cells constitutively express ovalbumin (OVA), which, upon uptake by antigen-presenting cells, is processed and cross-presented as OVA-derived peptides (e.g., SIINFEKL) on MHC class I molecules to downstream immune cells 55. This co-culture platform enables quantitative comparison of antigen presentation capacity and immune cell activation under different treatment conditions. *In vitro*, MCT-NE#9 treatment increased M1 macrophages, enhanced antigen presentation, and decreased TGFβ1 expression, leading to reduced T_regs_ and enhanced antitumor immunity. M2 macrophages remained unchanged, highlighting the association between ferroptosis and M1 macrophage activation.

*In vivo*, MCT-NE#9 induced sustained and cumulative ferroptosis in tumors as evidenced by increased CD71 expression, decreased GPX4 levels, and elevated LPO. Gene expression analysis revealed downregulation of ferroptosis-inhibiting genes and upregulation of ferroptosis-promoting genes. MCT-NE#9 also suppressed oxidative stress responses, amplifying tumor cell death more effectively than MTD.

Analysis of immune-related mRNA expression within the TME revealed upregulation of both antitumor immunity-associated genes and immunosuppressive genes, including *CD47*, *PD-L1*, *Muc1*, and *Ido1*. CD47 functions as an innate immune checkpoint by binding to SIRPα on macrophages, transmitting a “don't eat me” signal that inhibits phagocytosis. Within the TME, CD47 expression can be induced through IFN-γ-JAK/STAT/IRF1 signaling or by therapy-induced DNA damage pathways such as ATR/Chk1/STAT, and both cytotoxic chemotherapy and radiotherapy have been shown to increase CD47 levels 56,57. Similarly, PD-L1 expression can be upregulated through related interferon-mediated mechanisms 58-60. In this study, the observed increase in intratumoral IFN-γ mRNA provides a mechanistic explanation for the concurrent elevations of CD47 and PD-L1, suggesting compensatory checkpoint upregulation in response to immune activation. These findings support the rationale for combining MCT-NE#9 with anti-CD47 therapy to counteract adaptive immune resistance. Notably, *TGFβ1*, *Arg1*, *IL6*, and *CXCL12* were downregulated, improving immune modulation and aligning with reduced T_regs_. MCT-NE#9 significantly remodeled the TME by enhancing T-cell activation (indicated by increased CD25), boosting NK cells, and improving the M1/M2 macrophage ratio. Nevertheless, whether the observed immunomodulatory effects arise specifically from ferroptosis remains uncertain. *In vitro*, induction of ferroptosis enhanced macrophage phagocytosis, but this effect was markedly reduced by TLR2 antagonism (CU-CPT-22), whereas pharmacologic ferroptosis inhibition with Fer-1 produced only partial attenuation. These findings suggest that TLR2-dependent recognition contributes to—but does not fully explain—the phagocytic response. Consistent with recent reports, most cytotoxic agents do not activate a single death program; apoptosis and ferroptosis often occur concurrently, and inhibiting one pathway alone rarely prevents cell death 61-64. Moreover, immunogenic cell death is not unique to ferroptosis; apoptosis, necroptosis, and pyroptosis can also elicit immune responses under specific conditions 65,66. Accordingly, clarifying the ferroptosis-specific role in TME remodeling will require future *in vivo* studies using genetic or pharmacologic approaches that selectively modulate ferroptosis (e.g., GPX4 overexpression or knockdown), combined with comprehensive analyses of DAMP release and antigen-presentation dynamics. Immune cell analysis of TDLNs revealed increased levels of CD4, CD8, NK, and NKT cells, suggesting enhanced systemic immune activation. No significant changes in peripheral blood T cells were observed, indicating no immunotoxicity.

The optimized MCT-NE#9 formulation significantly increased oral bioavailability (659% for ATV and 851% for DTX) and permeability, enhancing therapeutic efficacy. In combination with anti-CD47 therapy, MCT-NE#9 substantially reduced tumor growth (up to 3.37-fold), induced sustained ferroptosis, and promoted macrophage-mediated antitumor immune responses. These findings highlight MCT-NE#9 as a promising, less invasive strategy for TNBC, offering enhanced efficacy with fewer side effects at lower doses. However, the present efficacy evaluation was limited to a 3-week treatment period, primarily assessing short-term tumor regression. Future studies incorporating long-term survival and recurrence models are needed to determine the durability of therapeutic responses to metronomic oral MCT-NE#9 and to evaluate its potential for preventing tumor relapse.

## Conclusions

This study presents MCT-NE#9, a novel oral MCT platform designed to induce sustained tumor ferroptosis and remodel the immune microenvironment in TNBC. By using DCK and Biotinyl PE to engage ASBT and SMVT, and TPGS to limit efflux, MCT-NE#9 greatly increased the oral bioavailability of ATV and DTX—by 659% and 851%, respectively. This dual-transporter-targeted strategy enabled efficient intestinal absorption and prolonged systemic exposure, overcoming key limitations of conventional oral chemotherapy. MCT-NE#9 effectively induced and sustained ferroptosis, as evidenced by elevated LPO, increased ROS generation, iron accumulation, and downregulation of GPX4. It also enhanced phagocytosis via TLR2, promoted M1 macrophage polarization, and increased antigen-presenting cell activity, while reducing TGFβ1, T_regs_, and M2 macrophages in the TME. When combined with anti-CD47 therapy, the formulation achieved synergistic tumor suppression (up to 70.3%) without systemic toxicity. These findings demonstrate that MCT-NE#9 not only improves drug delivery, but also amplifies immune-mediated anticancer effects through sustained ferroptosis-driven remodeling of the TME. As a fully oral, low-toxicity regimen integrating chemotherapy and immunotherapy, MCT-NE#9 offers a highly translational approach for effective TNBC treatment and represents a significant advancement in the design of multifunctional nanomedicine for systemic cancer therapy.

## Supplementary Material

Supplementary figures and tables.

## Abbreviations

Act D: Actinomycin D
ASBT: Apical sodium-dependent bile acid transporter
ATV: Atorvastatin
Biotinyl PE: Biotinylated phosphatidylethanolamine
BME: Basement membrane extract
CD: Cluster of differentiation
CFZ: Clofazimine
CLSM: Confocal laser scanning microscopy
CU-CPT22: TLR2 antagonist
Cys A: Cyclosporine A
DAPI: 4′,6-diamidino-2-phenylindole
DCK: L-lysine-linked deoxycholic acid
DOCA: Sodium deoxycholate
DOCA-TAP: Deoxycholic acid-DOTAP ionic complex
DOTAP: 1,2-dioleoyl-3-trimethylammonium-propane
DTX: Docetaxel
DMSO: Dimethyl sulfoxide
ECM: Extracellular matrix
ER: Endoplasmic reticulum
FACS: Fluorescence-activated cell sorting
FBS: Fetal bovine serum
Fer-1: Ferrostatin-1
FT-IR: Fourier transform infrared spectroscopy
GPX4: Glutathione peroxidase 4
HBSS: Hank's balanced salt solution
HMGB1: High mobility group box 1 protein
HPLC: High-performance liquid chromatography
IHC: Immunohistochemistry
iNOS: Inducible nitric oxide synthase
IP: Intraperitoneal
IV: Intravenous
LPO: Lipid peroxidation
MBCD: Methyl-β-cyclodextrin
MCT: Metronomic chemotherapy
MCT-NE: Metronomic chemotherapy nanoemulsion
MDCK: Madin-Darby canine kidney
MDSC: Myeloid-derived suppressor cell
MFI: Mean fluorescence intensity
MTD: Maximum tolerated dose
NE: Nanoemulsion
NK: Natural killer
NKT: Natural killer T cell
OST_α/β_: Organic solute transporter α/β
PA: Pantothenic acid
PBS: Phosphate-buffered saline
*P_app_*: Apparent permeability
PDI: Polydispersity index
P-gp: P-glycoprotein
ROS: Reactive oxygen species
RSL3: RAS-selective lethal 3
RT-qPCR: Reverse transcription quantitative polymerase chain reaction
SMVT: Sodium-dependent multivitamin transporter
TEM: Transmission electron microscopy
TLR2: Toll-like receptor 2
TPGS: D-α-tocopheryl polyethylene glycol 1000 succinate
UPLC-MS/MS: Ultra-performance liquid chromatography tandem mass spectrometry
