# Supplementary material for: Ligand-directed oral lipidic nanoplatform enables sustained ferroptosis and immune reprogramming via multivalent transporter-mediated metronomic delivery
Source: Theranostics. 2026 Jan 1;16(6):2952–83. doi: 10.7150/thno.124189 (PMC12775813; doi:10.7150/thno.124189)
Supplement: Supplementary file 1 — Supplementary figures and tables. [file thnov16p2952s1.pdf]

Supporting Information

Ligand-directed oral lipidic nanoplatform enables sustained ferroptosis and immune reprogramming via multivalent transporter-mediated metronomic delivery

Authors' names and affiliations:

Laxman Subedi[1,2,§], In Ho Im[3,4,§], Arjun Dhwoj Bamjan[1], Jiwon Jeon[3,4], Susmita Phuyal[1], Yun-Hwa Jeong[3,4], Seung Hyun Kim[3,4], Jung-Hyun Shim[1,5], Jeong Uk Choi[3,4,*], Jin Woo Park[1,5,*]

1. Department of Biomedicine, Health & Life Convergence Sciences, BK21 Four, Biomedical and Healthcare Research Institute, Mokpo National University, Jeonnam 58554, Republic of Korea
2. Biomedicine Cutting Edge Formulation Technology Center, Mokpo National University, Jeonnam 58554, Republic of Korea
3. Department of Regulatory Science, Graduate School, Kyung Hee University, Seoul 02447, Republic of Korea
4. College of Pharmacy, Kyung Hee University, Seoul 02447, Republic of Korea
5. College of Pharmacy and Natural Medicine Research Institute, Mokpo National University, Jeonnam 58554, Republic of Korea

[*]Corresponding authors: cju0667@khu.ac.kr (Jeong Uk Choi), jwpark@mokpo.ac.kr (Jin Woo Park)

[§]These authors contributed equally to this work.

Running head: Oral metronomic chemotherapy induces ferroptosis and immune modulation

Supplementary Tables

Table S1. Antibody information for western blot and flow cytometry.

Antibodies	Manufacturer	Catalog number
PerCP anti-mouse CD45	Biolegend	103129
PE anti-mouse CD3	Biolegend	100205
APC/Cy7 anti-mouse CD4	Biolegend	100413
APC anti-mouse CD8	Biolegend	140419
FITC anti-mouse CD25	Biolegend	101907
PE/Cy7 anti-mouse IFN-gamma	Biolegend	505850
Pacific blue anti-mouse Foxp3	Biolegend	126409
FITC anti-mouse CD11b	Biolegend	101205
PE/Cy7 anti-mouse F4/80	Biolegend	123113
APC/Cy7 anti-mouse CD86	Biolegend	105029
Pacific blue anti-mouse iNOS	Biolegend	48-5920-82
APC anti-mouse CD206	Biolegend	141707
Pacific blue anti-mouse Arginase	Biolegend	48-3697-82
APC anti-mouse CD49b	Biolegend	108909
PE anti-mouse CD11c	Biolegend	117307
PE anti-mouse CD71	Biolegend	113808
APC/Cy7 anti-mouse CD47	Biolegend	127525
PE/Cy7 anti-mouse Ly6C	Biolegend	128018

APC anti-mouse Ly6G	Biolegend	127614
FITC anti-mouse CD4	Biolegend	100405
PE/Cy7 anti-mouse CD8	Biolegend	100722
APC anti-mouse CD86	Biolegend	159216
Pacific blue anti-mouse CD11c	Biolegend	117321
APC anti-mouse iNOS	Biolegend	17-5920-80
PE anti-mouse MHC-I SIINFEKLC	Biolegend	141603
FITC anti-mouse I-A/I-E (MHCll)	Biolegend	107605
APC anti-mouse H-2Kd (MHCl)	Biolegend	116619
GPX4(E5Y8K) Rabbit mAb	Cell signaling	#59735
xCT/SLC7A11 Antibody	Cell signaling	#98051S
GAPDH(D16H11) Rabbit mAb	Cell signaling	#5174
Goat anti-rabbit IgG, polyclonal antibody (HRP conjugate)	ENZO	ADI-SAB-300-J
Goat anti-mouse IgG F(ab')2, polyclonal antibody (HRP conjugate)	ENZO	ADI-SAB-100-J

Table S2. MCT-NE composition.

Formulation code	ATV (mg)	DTX (mg)	Capryol 90 (mg)	Tween 80 (mg)	Transcutol HP (mg)	TPGS (mg)	DCK (mg)	DOCA (mg)	DOTAP (mg)	DOCA-TAP (mg)	Biotinyl PE (mg)	DCK/Biotinyl PE (mg)
MCT-NE#1	1	4	16.8	100	200							
MCT-NE#2	1	4	16.8	100	200	20						
MCT-NE#3	1	4	16.8	100	200	20	8.63					
MCT-NE#4	1	4	16.8	100	200	20		6.27				
MCT-NE#5	1	4	16.8	100	200	20			10.56			
MCT-NE#6	1	4	16.8	100	200	20				16.83		
MCT-NE#7	1	4	16.8	100	200	20					15	
MCT-NE#8	1	4	16.8	100	200	20				16.83	15	
MCT-NE#9	1	4	16.8	100	200	20						23.63

Table S3. Inhibitors and their concentrations used in the transport study, along with their respective functions.

Inhibitor	Concentration	Function
Act D	3.2 μM	Blocks ASBT-dependent uptake of bile acid–conjugated molecules
CFZ	10 μM	Suppresses $OST_{\alpha/\beta}$ activity, limiting basolateral export of bile acid substrates
PA	0.2 mM	Interferes with SMVT-driven transport of multivitamins and related ligands
Chlorpromazine	32 μM	Inhibits clathrin-coated vesicle–mediated internalization
MBCD	10 mM	Disrupts caveola and lipid raft pathways by depleting membrane cholesterol
Genistein	0.1 mM	Reduces caveola/lipid raft endocytosis via tyrosine kinase inhibition
Amiloride	0.1 mM	Prevents macropinocytic uptake through Na^+/H^+ exchange inhibition
Brefeldin A	90 μM	Blocks ER-to-Golgi trafficking and vesicular transport processes
Cys A	10 μM	Inhibits P-gp efflux activity and increases intracellular drug retention

Act D, actinomycin D; ASBT, apical sodium-dependent bile acid transporter; CFZ, clofazimine; $OST_{\alpha/\beta}$, organic solute transporter α and β; PA, pantothenic acid; SMVT, sodium-dependent multivitamin transporter; MBCD, methyl-β-cyclodextrin; ER, endoplasmic reticulum; Cys A, cyclosporine A; P-gp, P-glycoprotein.

Table S4. Mean droplet sizes, polydispersity indices, zeta potentials, and encapsulation efficiencies of MCT-NEs.

Test material	Droplet size (nm)	Polydispersity index (PDI)	Zeta potential (mV)	Encapsulation efficiency (%)	
				ATV	DTX
ATV/DTX in water	9629 ± 489	0.705 ± 0.410		17.3 ± 0.672	23.9 ± 0.048
ATV/DTX in 0.5% DMSO	587 ± 7.24	1.000 ± 0.000		90.0 ± 0.343	100 ± 0.048
MCT-NE#1	8.74 ± 0.095	0.197 ± 0.011	−1.96 ± 0.015	99.5 ± 0.268	101 ± 0.094
MCT-NE#2	8.78 ± 0.036	0.207 ± 0.007	−2.16 ± 0.021	98.6 ± 0.279	101 ± 0.675
MCT-NE#3	22.6 ± 0.595	0.429 ± 0.015	0.146 ± 0.034	98.6 ± 0.246	101 ± 0.283
MCT-NE#4	21.8 ± 0.651	0.384 ± 0.017	−5.27 ± 0.035	98.3 ± 0.417	100 ± 0.102
MCT-NE#5	43.5 ± 1.23	0.341 ± 0.029	0.54 ± 0.032	54.1 ± 0.207	76.2 ± 0.775
MCT-NE#6	16.2 ± 0.214	0.328 ± 0.024	−3.35 ± 0.015	104 ± 0.820	100 ± 0.111
MCT-NE#7	12.6 ± 0.066	0.274 ± 0.007	−6.01 ± 0.038	99.4 ± 0.440	101 ± 0.245
MCT-NE#8	16.7 ± 0.081	0.304 ± 0.018	−4.22 ± 0.025	100 ± 0.268	99.2 ± 1.20
MCT-NE#9	15.8 ± 0.065	0.332 ± 0.011	−2.78 ± 0.015	98.3 ± 0.239	100 ± 0.299

Data are presented as mean ± SD (n = 3).

Table S5. Stability of MCT-NE#9 after 1 month of storage under ambient and accelerated conditions.

Storage condition	Week	Drug content (%)		Cumulative drug release at 2 h (%)		Droplet size (nm)	Polydispersity index (PDI)	Zeta potential (mV)
		ATV	DTX	ATV	DTX			
25 ± 2 °C with 65 ± 5% RH	0	101 ± 1.23	99.7 ± 2.26	91.7 ± 1.11	97.1 ± 2.00	15.5 ± 0.509	0.330 ± 0.023	−3.38 ± 0.201
	1	97.1 ± 1.29	97.9 ± 2.32	92.8 ± 2.75	97.6 ± 1.83	14.3 ± 0.066	0.358 ± 0.006	−2.83 ± 0.672
	2	98.7 ± 1.84	98.3 ± 2.20	93.6 ± 2.88	98.8 ± 2.95	14.5 ± 0.095	0.291 ± 0.013	−3.94 ± 0.405
	3	99.0 ± 1.26	99.3 ± 1.47	91.6 ± 1.53	99.6 ± 0.83	14.9 ± 0.147	0.373 ± 0.012	−3.83 ± 0.405
	4	102 ± 2.26	98.8 ± 1.73	94.1 ± 2.46	96.9 ± 1.64	13.0 ± 0.453	0.283 ± 0.015	−3.47 ± 0.523
40 ± 2 °C with 75 ± 5% RH	1	99.0 ± 2.27	100 ± 1.56	93.0 ± 2.28	97.7 ± 2.14	14.8 ± 0.092	0.330 ± 0.022	−4.11 ± 0.419
	2	96.5 ± 0.885	96.8 ± 0.942	92.6 ± 0.881	98.1 ± 1.60	15.4 ± 0.220	0.327 ± 0.018	−2.46 ± 0.481
	3	102 ± 1.60	102 ± 1.85	91.7 ± 1.39	97.5 ± 2.18	14.1 ± 0.244	0.264 ± 0.017	−4.30 ± 0.402
	4	100 ± 2.73	98.1 ± 2.71	92.6 ± 1.89	99.5 ± 1.85	13.3 ± 0.149	0.261 ± 0.023	−4.44 ± 0.757

Data are presented as mean ± SD (n = 3).

Table S6. Pharmacokinetic parameters for ATV and DTX in rats after intravenous (IV) or oral administration of the free drugs and MCT-NE#9.

Test material	ATV-IV	ATV-oral	MCT-NE#9 (ATV)	DTX-IV	DTX-oral	MCT-NE#9 (DTX)
Administration route	IV	Oral	Oral	IV	Oral	Oral
ATV dose (mg/kg)	2.5	2.5	2.5			
DTX dose (mg/kg)				10	10	10
T_{max} (h)		4.00 ± 0.00	4.00 ± 0.00		4.50 ± 1.00	4.00 ± 0.00
$T_{1/2}$ (h)	1.26 ± 0.156	1.24 ± 0.358	2.43 ± 0.781	8.59 ± 0.624	Not applicable	76.7 ± 116
C_{max} (ng/mL)	259 ± 50.2	1.07 ± 0.144	7.34 ± 0.830	776 ± 19.0	10.6 ± 1.28	60.1 ± 14.7
AUC_{last} (ng·h/mL)	267 ± 28.5	4.41 ± 1.02	33.5 ± 2.01	1570 ± 243	35.1 ± 11.1	335 ± 60.0
AUC_{inf} (ng·h/mL)	270 ± 30.0	4.45 ± 1.02	33.6 ± 2.03	1787 ± 294	Not applicable	1232 ± 1571
Bioavailability (%)	100	1.66 ± 0.381	12.6 ± 0.753	100	2.24 ± 0.709	21.3 ± 3.82

Values are shown as means ± SD (n = 4).

Supplementary Figures

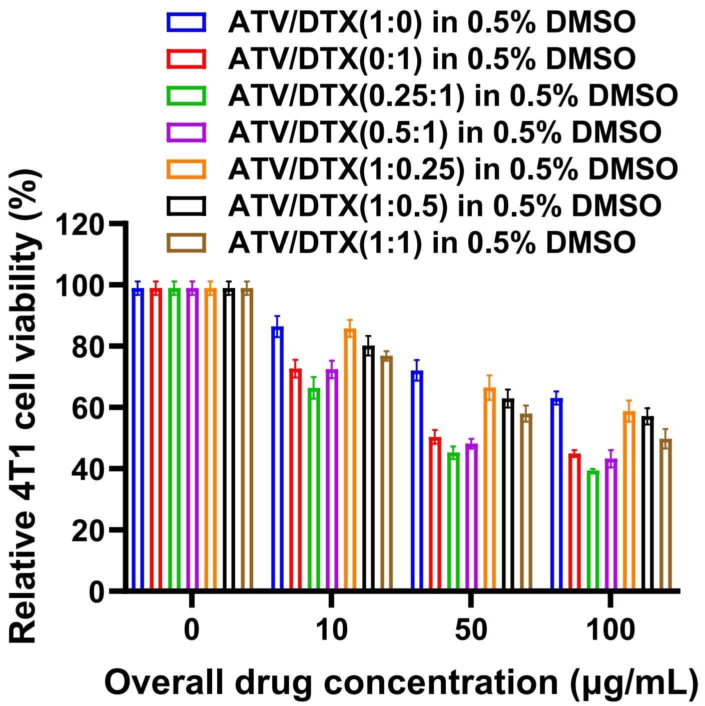

Figure S1. In vitro cytotoxicity of ATV/DTX prepared in 0.5% DMSO was evaluated in 4T1 cells at various ATV-to-DTX ratios. Data are presented as mean ± SD (n = 4).

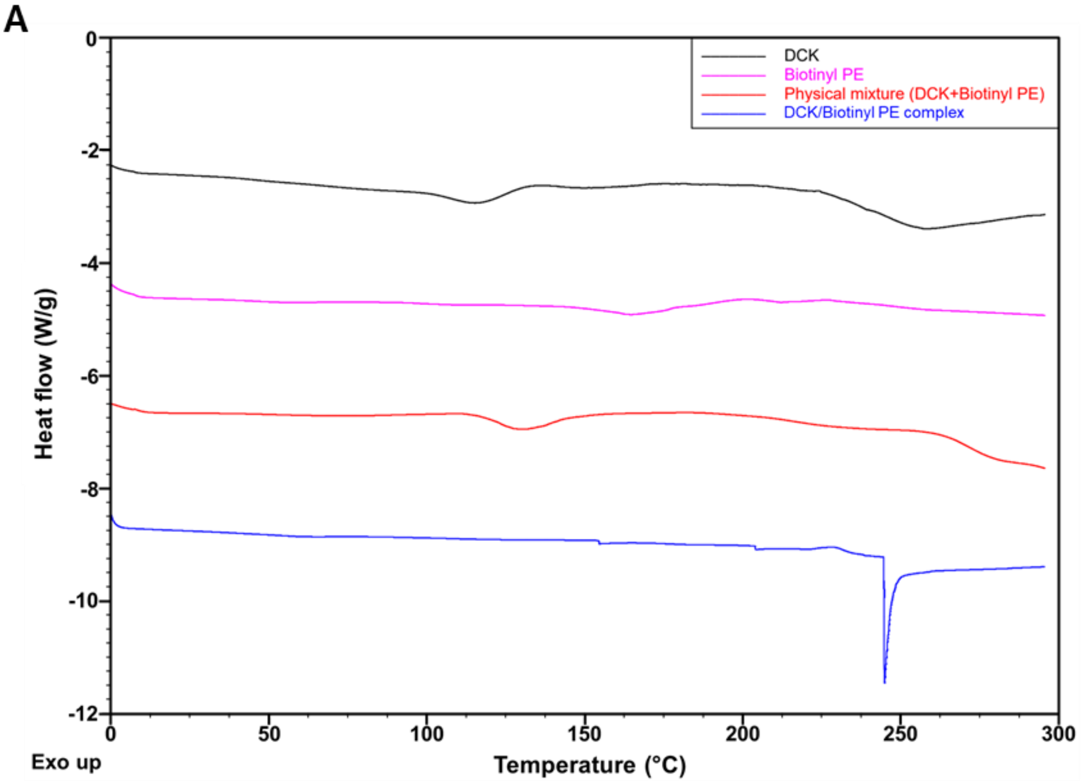

A
DCK
Biotinyl PE
Physical mixture (DCK+Biotinyl PE)
DCK/Biotinyl PE complex
Heat flow (W/g)
Exo up
Temperature (°C)

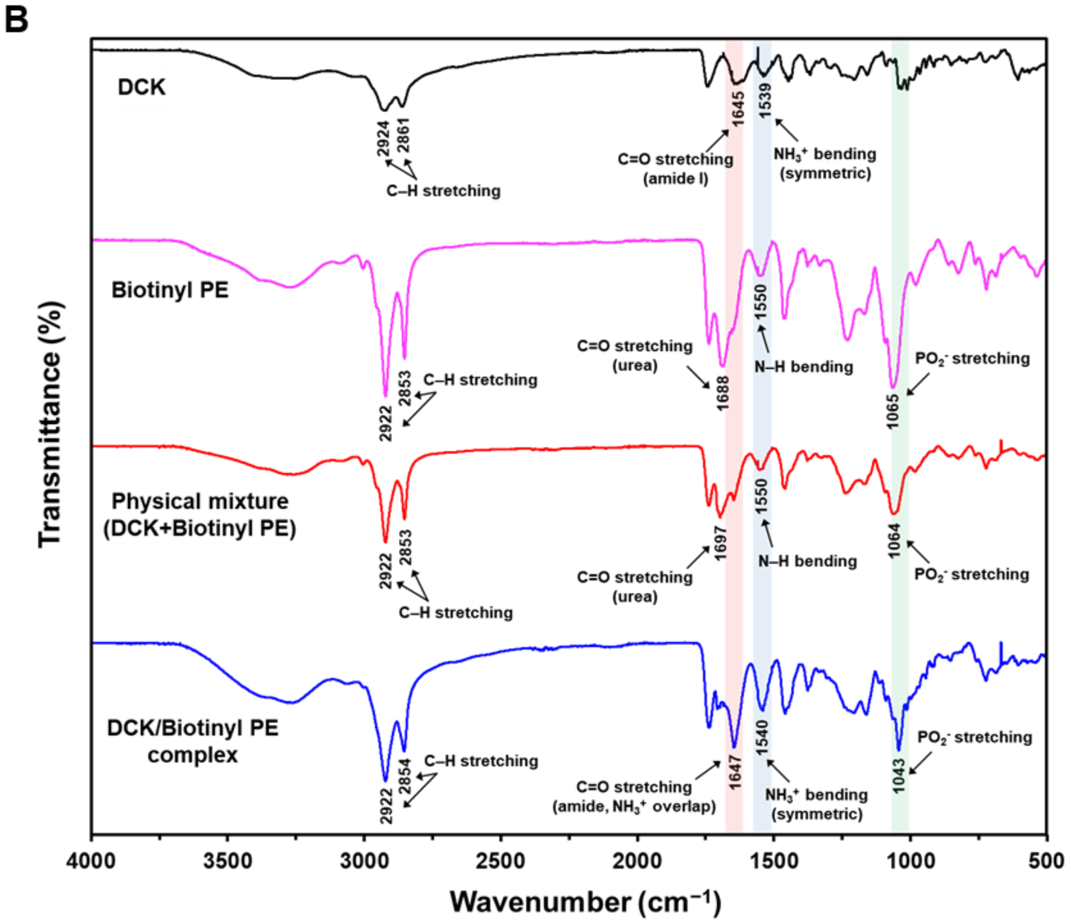

B
DCK
2924
2861
C–H stretching
1645
C=O stretching (amide I)
1539
NH3+ bending (symmetric)
Biotinyl PE
2922
2853
C–H stretching
C=O stretching (urea)
1688
1550
N–H bending
1065
PO2- stretching
Physical mixture (DCK+Biotinyl PE)
2922
2853
C–H stretching
1697
C=O stretching (urea)
1550
N–H bending
1064
PO2- stretching
DCK/Biotinyl PE complex
2922
2854
C–H stretching
1647
C=O stretching (amide, NH3+ overlap)
1540
NH3+ bending (symmetric)
1043
PO2- stretching
Transmittance (%)
Wavenumber (cm−1)

Figure S2. Thermal and spectroscopic analyses of the ionic complex formed between L-lysine-linked deoxycholic acid (DCK) and Biotinyl PE (**A**) Differential scanning calorimetry (DSC) thermograms of DCK, Biotinyl PE, their physical mixture, and the DCK/Biotinyl PE complex. (**B**) Fourier transform infrared (FT-IR) spectra of DCK, Biotinyl PE, their physical mixture, and the DCK/Biotinyl PE complex.

A

B

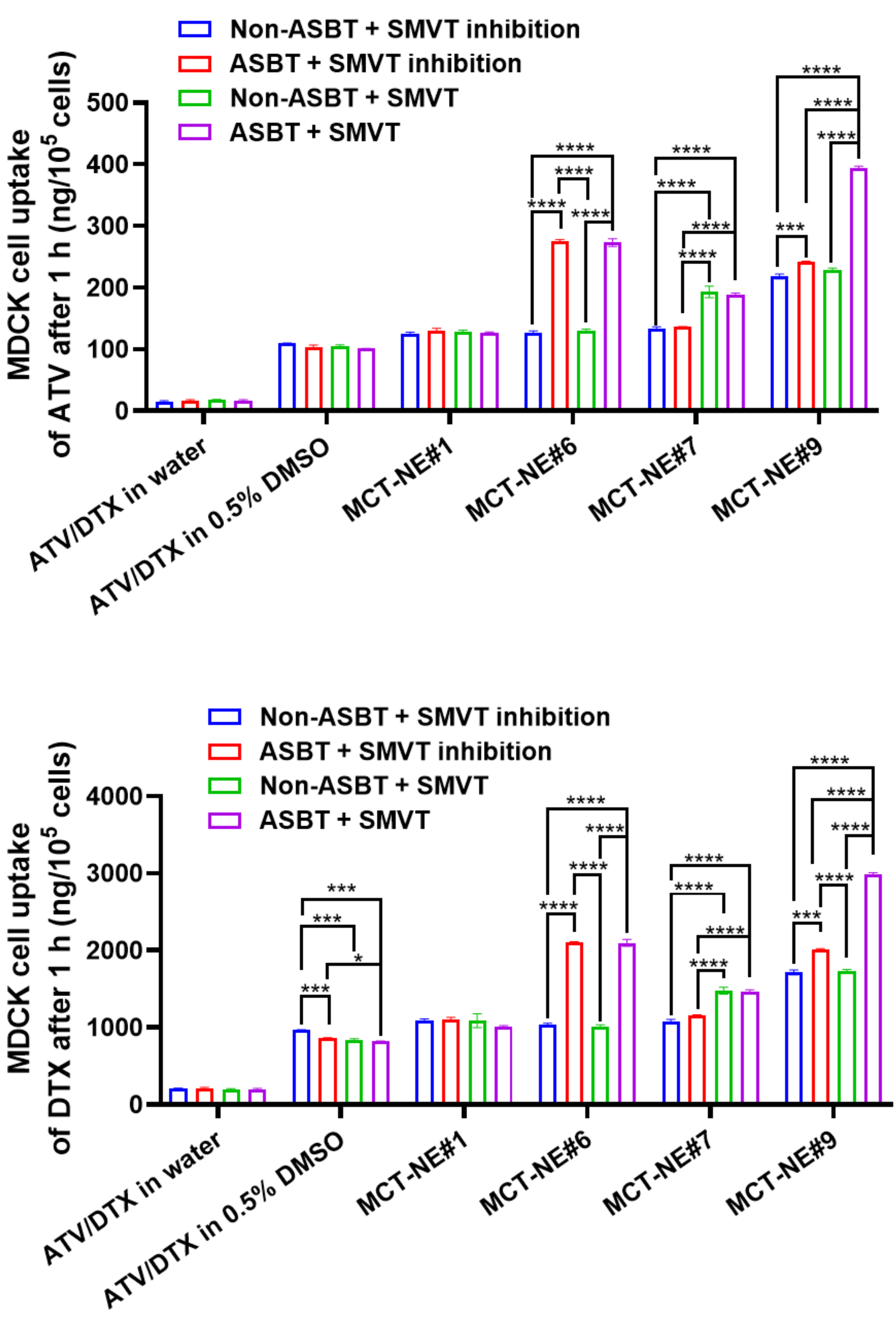

Figure S3. Intracellular accumulation of ATV and DTX in ASBT-expressing and non-

transfected MDCK cells. Quantification of (**A**) ATV and (**B**) DTX uptake in ASBT-positive and ASBT-negative MDCK cells treated with or without SMVT blockade for 1 h. Treatment conditions included ATV/DTX formulated in water, ATV/DTX prepared in 0.5% DMSO, and MCT-NEs. Data are presented as mean ± SD (n = 3). * $p < 0.05$, *** $p < 0.001$, **** $p < 0.0001$.

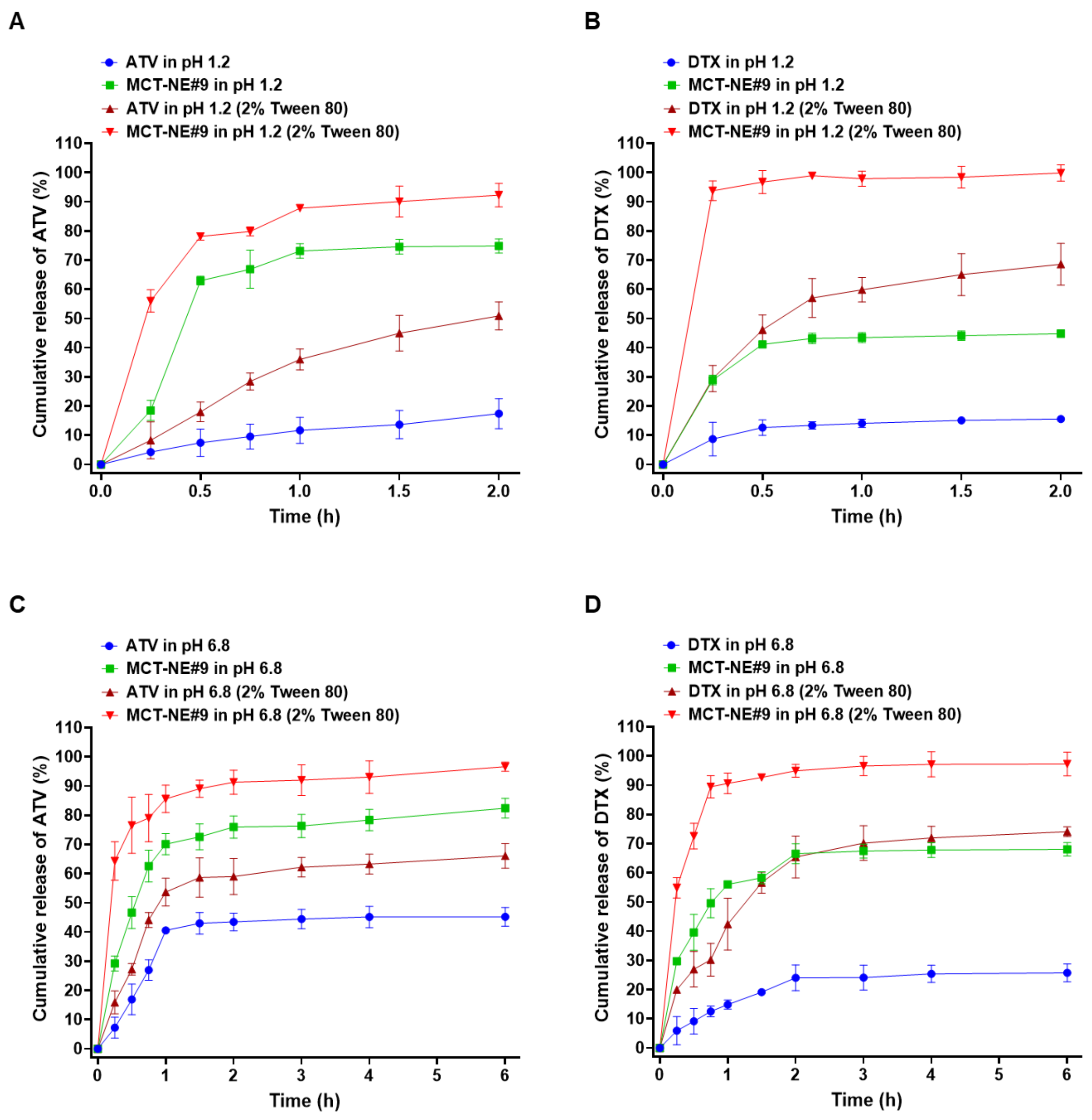

Figure S4. In vitro cumulative release profiles of (**A**) ATV and (**B**) DTX in dissolution medium at pH 1.2 (0.1 N HCl), and (**C**) ATV and (**D**) DTX at pH 6.8 (PBS), from MCT-NE#9 compared with their respective free drug forms, each tested either in the absence or presence of 2% (w/v) Tween 80. Data are expressed as the mean ± SD (n = 6).

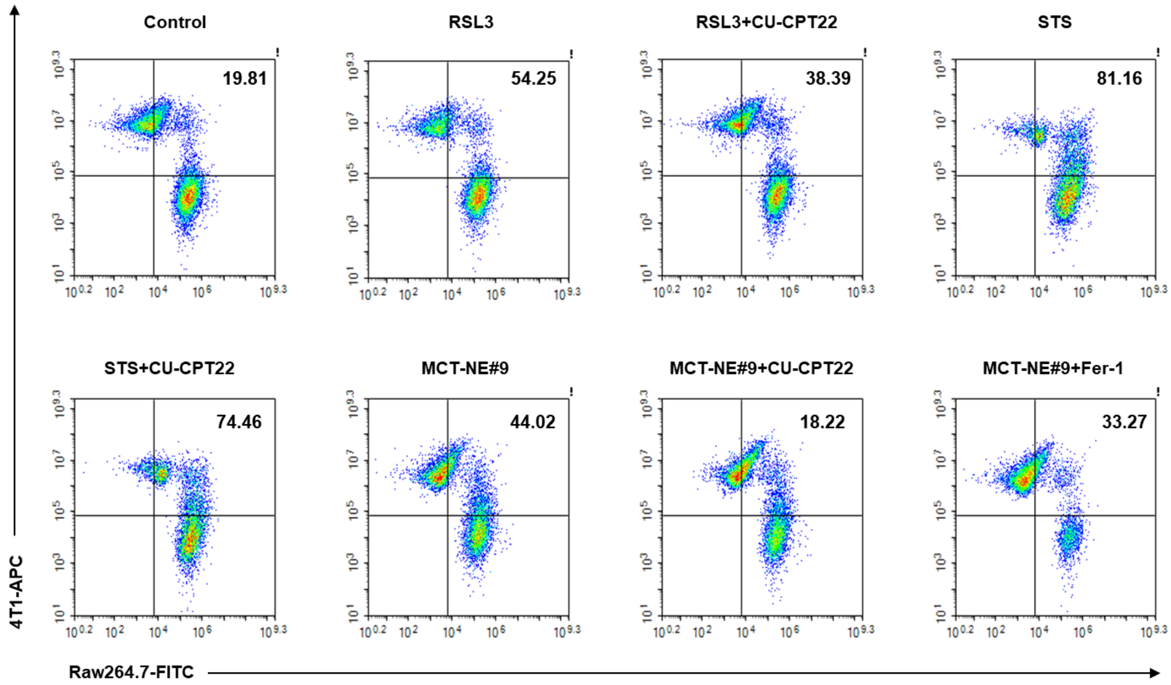

Figure S5. In vitro macrophage phagocytosis. Dot plot analysis of phagocytosis after treatment with RSL3, STS, MCT-NE#9, and inhibitors (CU-CPT22 and Fer-1). Flow cytometry was used to assess the interaction between 4T1 cancer cells (allophycocyanin) and Raw 264.7 macrophages (fluorescein isothiocyanate), with the percentages of phagocytosed cells indicated in each quadrant.

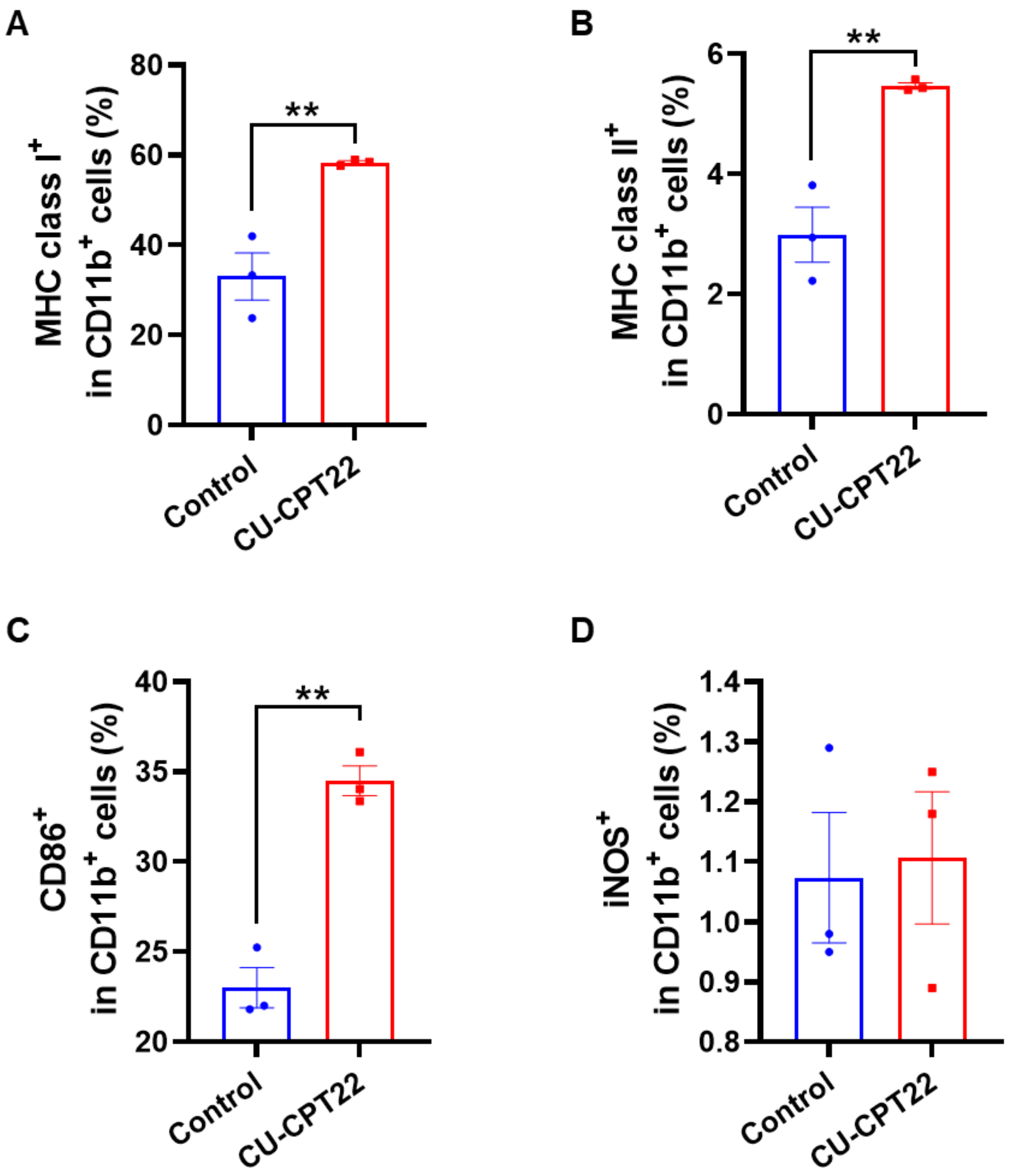

Figure S6. Flow cytometry analysis of Raw 264.7 macrophages treated with CU-CPT22. Quantification of (**A**) MHC class I and (**B**) MHC class II expression. (**C**) Quantification of CD 86 expression. (**D**) Quantification of iNOS expression. Values are shown as means ± SEM (n = 3). ** $p < 0.01$.

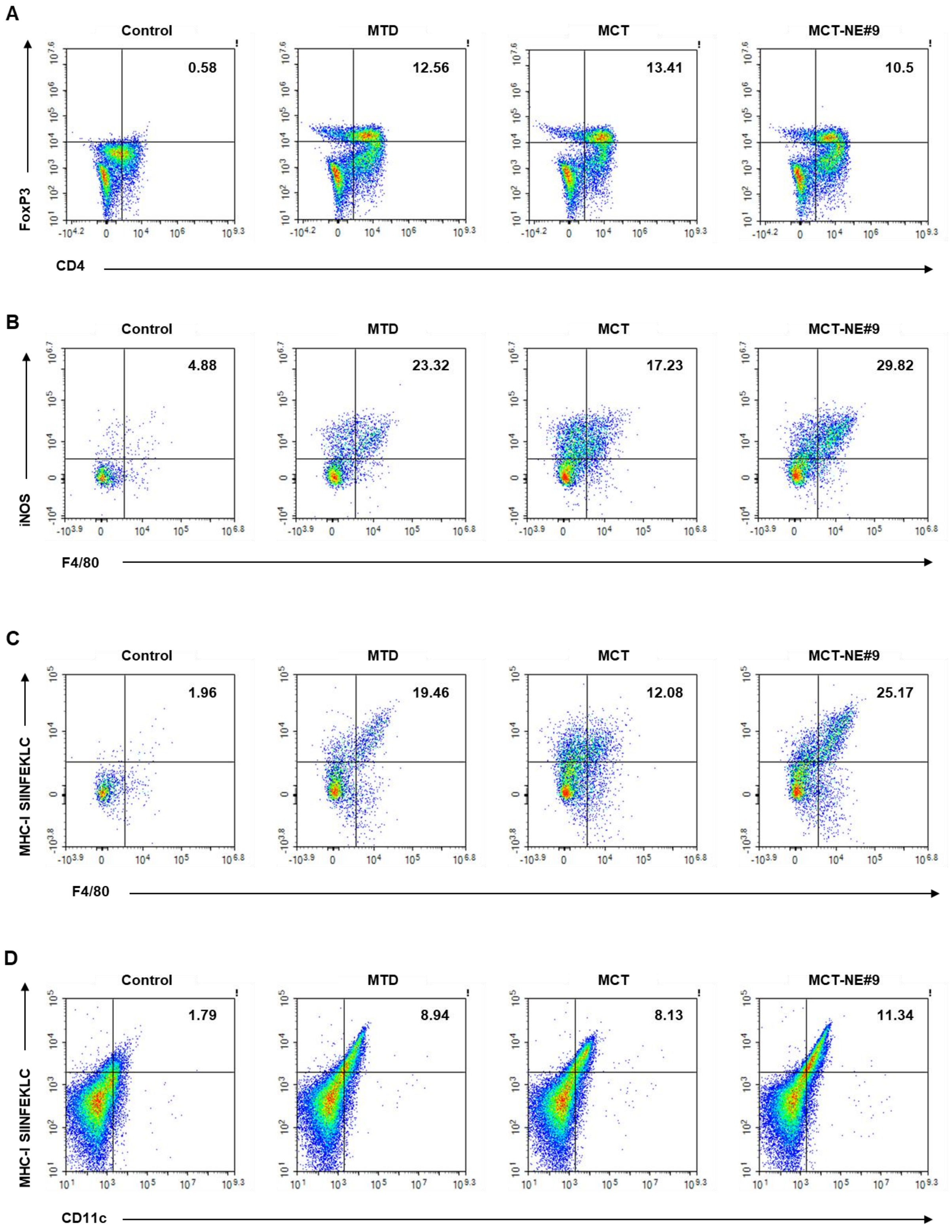

Figure S7. Ex vivo immune analysis in B16F10.OVA cells co-cultured with splenocytes. Dot plot of ex vivo analysis results after treatment with control, MTD, MCT, and MCT-NE#9. (**A**) Flow cytometry dot plots showing T_{regs} ($CD4^+$, $Foxp3^+$), (**B**) M1 macrophages ($F4/80^+$, $iNOS^+$), (**C**) antigen-presenting macrophages (MHC-I

SIINFEKL$^+$, F4/80$^+$), and (**D**) antigen-presenting dendritic cells (MHC-I SIINFEKL$^+$, CD11c$^+$). Numbers indicate the percentages of gated populations.

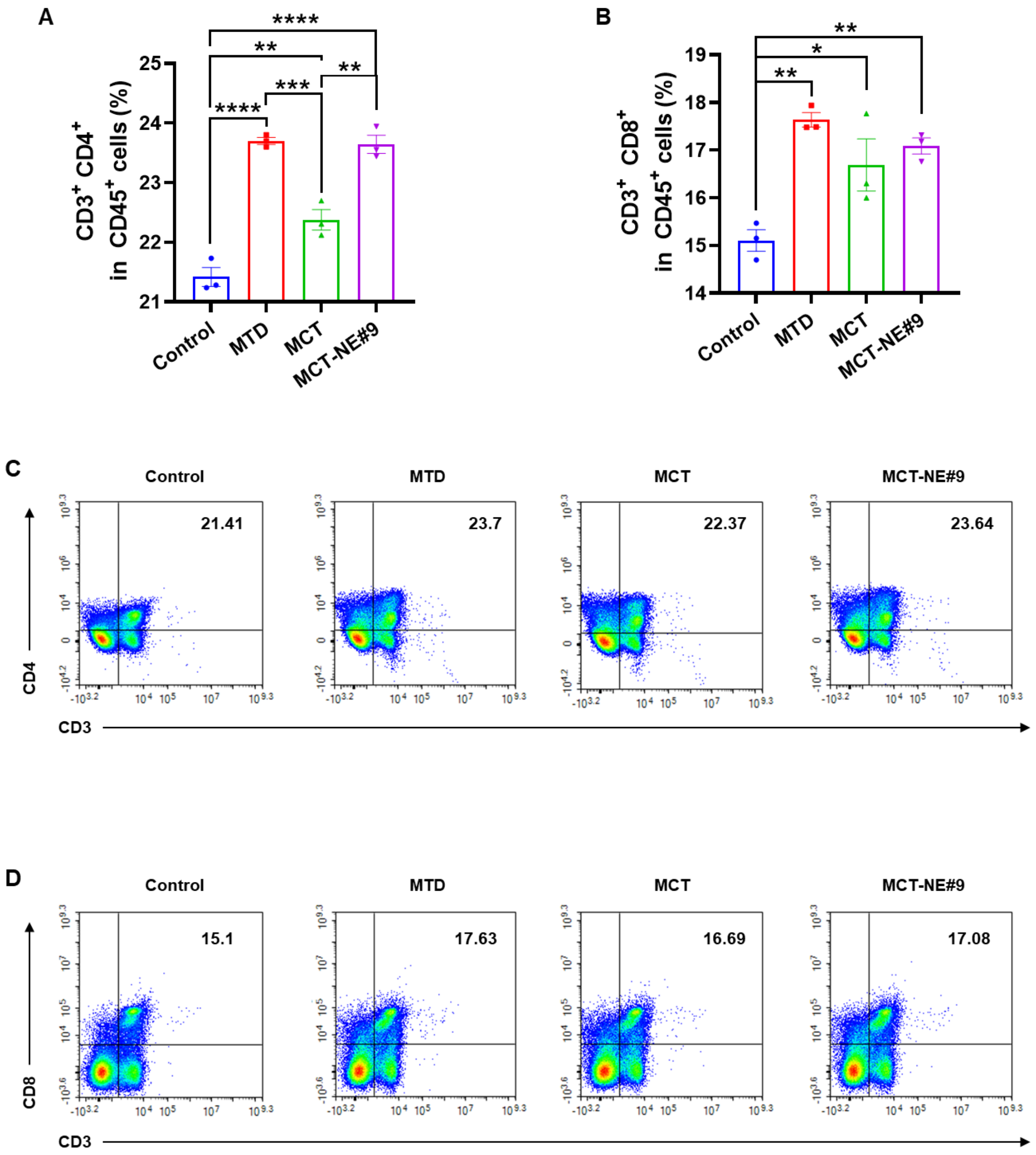

Figure S8. Ex vivo analysis of CD4 and CD8 T cells after co-culture with B16F10.OVA cells. Quantification of (**A**) CD4 T cells and (**B**) $CD8^+$ T cells in $CD45^+$ splenocytes after 24 h of co-culture with B16F10.OVA cells treated with MTD, MCT, and MCT-NE#9. Representative flow cytometry dot plots showing the percentages of (**C**) CD4 T cells ($CD3^+$, $CD4^+$) and (**D**) CD8 T cells ($CD3^+$, $CD8^+$) among $CD3^+$ splenocytes. Numbers indicate the percentages of gated population. Values are shown as means ± SEM (n = 3). * $p < 0.05$, ** $p < 0.01$, *** $p < 0.001$, **** $p < 0.0001$.

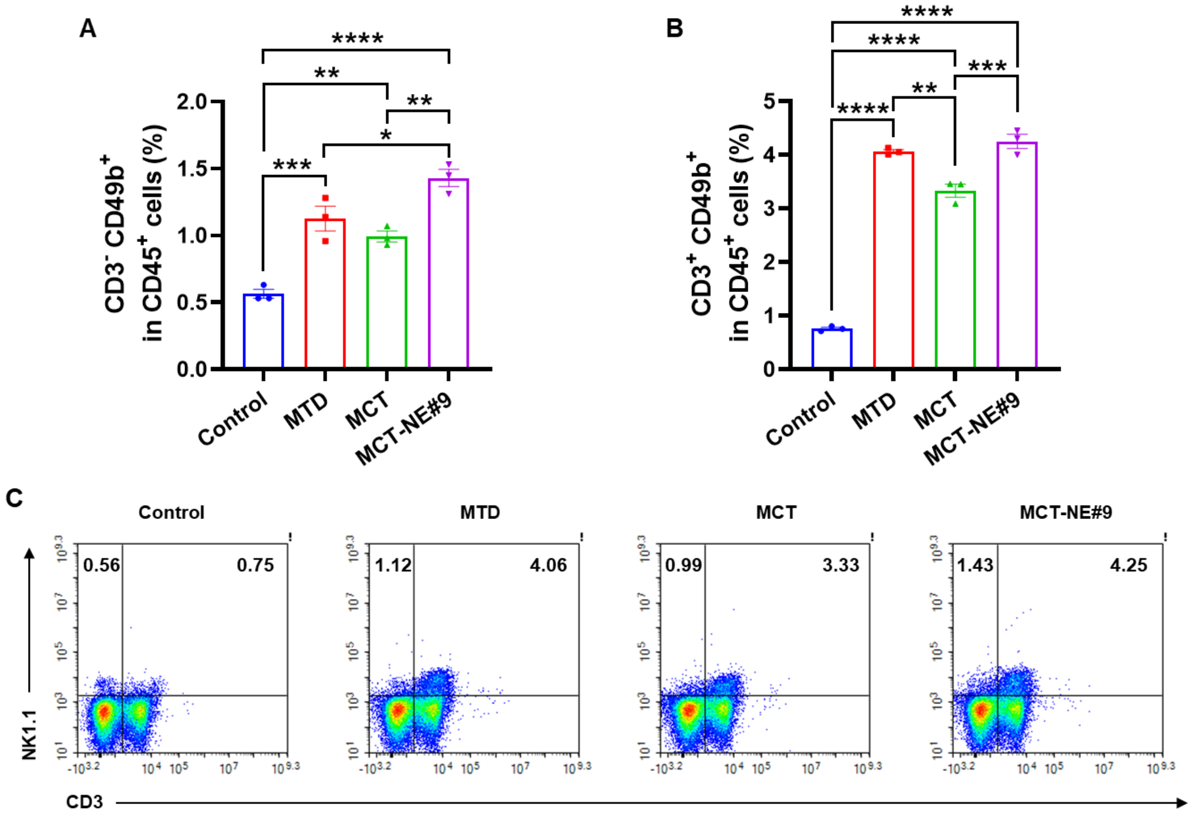

Figure S9. Ex vivo analysis of NK and NKT cells after co-culture with B16F10.OVA cells. Quantification of (**A**) NK cells and (**B**) NKT cells among CD45⁺ splenocytes after 24 h of co-culture with B16F10.OVA cells treated with MTD, MCT, and MCT-NE#9. (**C**) Representative flow cytometry dot plots showing the percentages of NK cells ($CD3^-$, $NK1.1^+$) and NKT cells ($CD3^+$, $NK1.1^+$) among $CD45^+$ splenocytes. Numbers indicate the percentages of gated populations. Results are presented as mean ± SEM (n = 3). * $p < 0.05$, ** $p < 0.01$, *** $p < 0.001$, **** $p < 0.0001$.

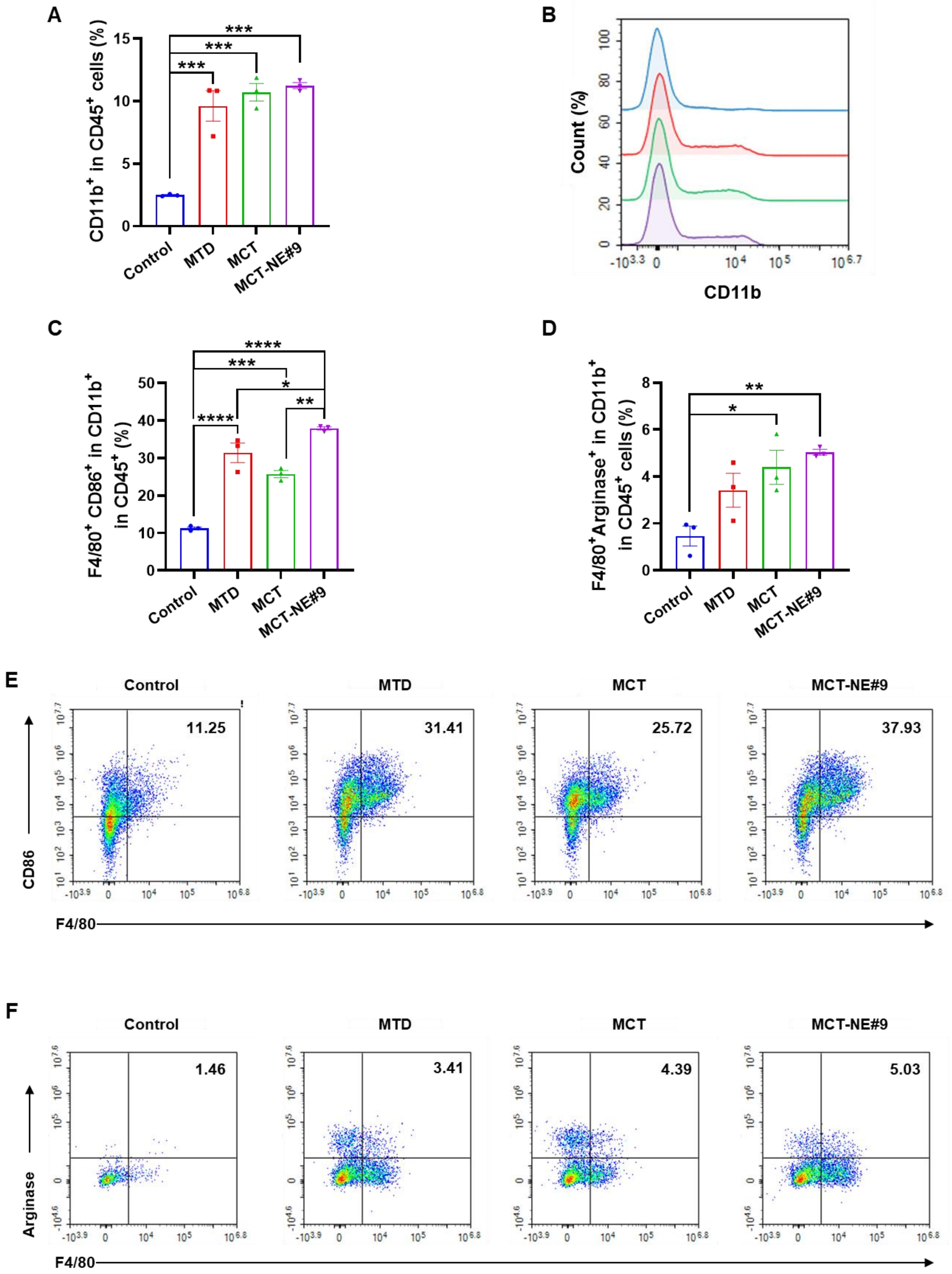

Figure S10. Ex vivo analysis of macrophages after co-culture with B16F10.OVA cells. (**A**) Quantification and (**B**) histogram of CD11b$^+$ cells among CD45$^+$ splenocytes. Quantification of (**C**) CD86$^+$ M1 macrophages and (**D**) Arginase$^+$ M2 macrophages

among $CD11b^+$ populations. Representative flow cytometry dot plots showing the percentages of (**E**) $CD86^+$ M1 macrophages ($F4/80^+$, $CD86^+$) and (**F**) $Arginase^+$ M2 macrophages ($F4/80^+$, $Arginase^+$) among $CD11b^+$ splenocytes. Numbers indicate the percentages of gated populations. Results are presented as mean ± SEM (n = 3). * $p < 0.05$, ** $p < 0.01$, *** $p < 0.001$, **** $p < 0.0001$.

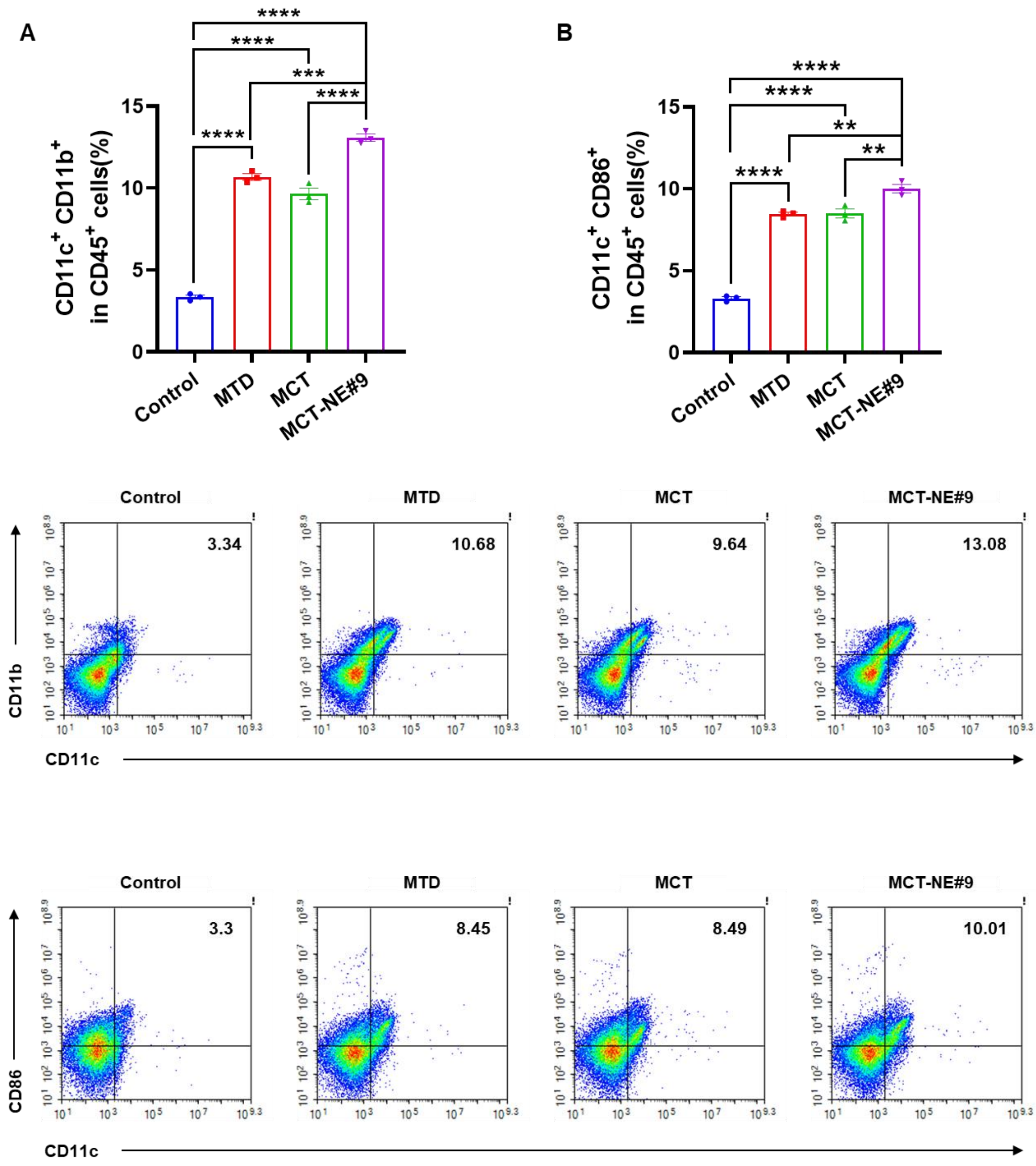

Figure S11. Ex vivo analysis of dendritic cells after co-culture with B16F10.OVA cells. Quantification of (**A**) $CD11c^+$, $CD11b^+$ dendritic cells and (**B**) $CD11c^+$, $CD86^+$ activated dendritic cells among $CD45^+$ splenocytes. Representative flow cytometry dot plots showing the percentages of (**C**) $CD11c^+$, $CD11b^+$ dendritic cells and (**D**) $CD11c^+$, $CD86^+$ activated dendritic cells among $CD45^+$ splenocytes. Numbers indicate the percentages of gated populations. Values are shown as means ± SEM (n = 3). ** $p < 0.01$, *** $p < 0.001$, **** $p < 0.0001$.

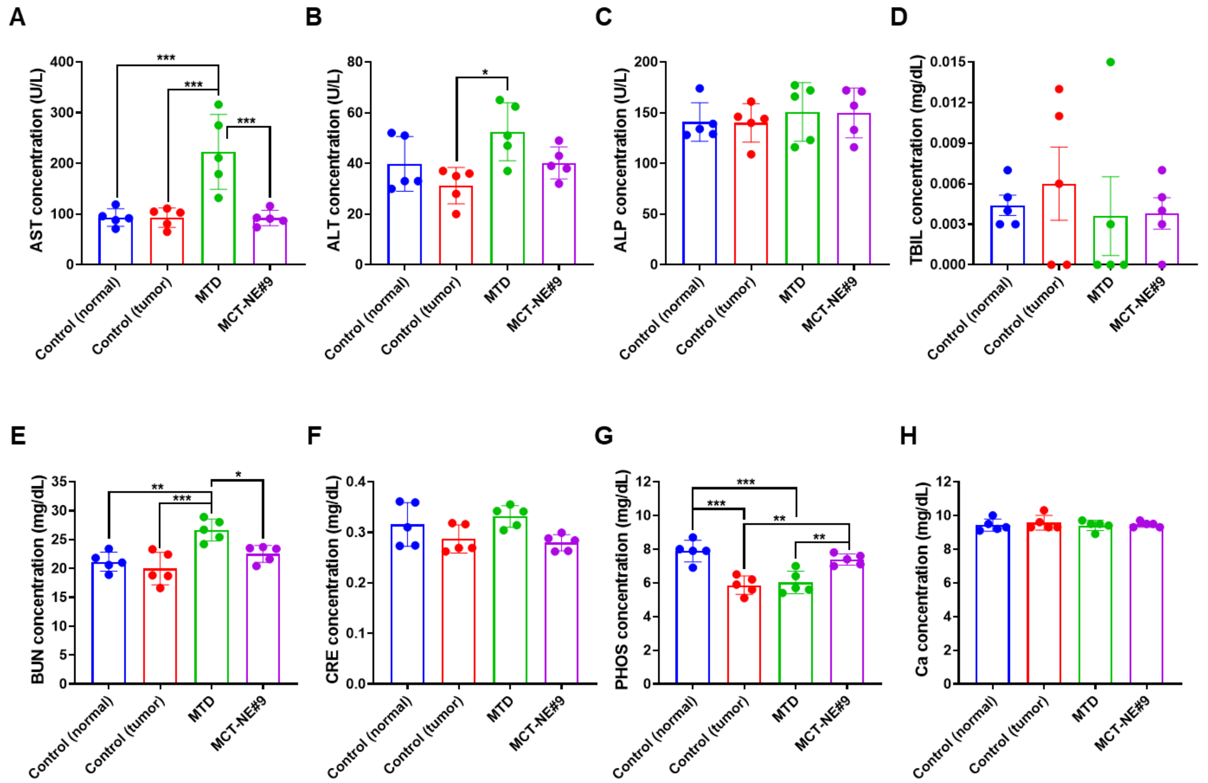

Figure S12. Serum biochemical parameters measured after 4 weeks of treatment in 4T1 tumor-bearing mice. (**A**) Aspartate aminotransferase (AST), (**B**) alanine aminotransferase (ALT), (**C**) alkaline phosphatase (ALP), (**D**) total bilirubin (TBIL), (**E**) blood urea nitrogen (BUN), (**F**) creatinine (CRE), (**G**) inorganic phosphorus (PHOS), and (**H**) calcium (Ca). Data are shown as mean ± SD (n = 5). * $p < 0.05$, ** $p < 0.01$, *** $p < 0.001$.

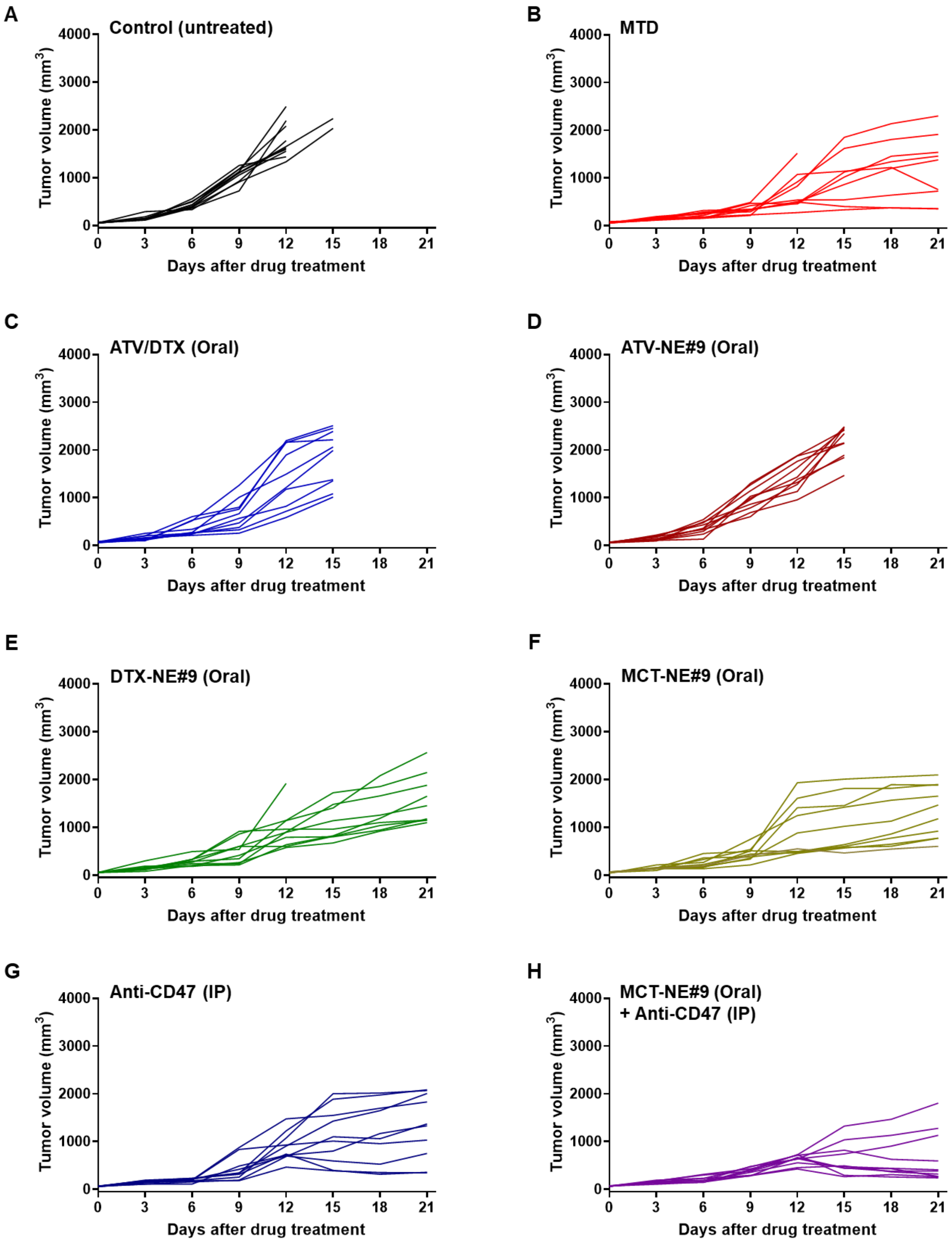

Figure S13. Individual tumor volume of each mouse in each group. Antitumor efficacy of oral MCT-NE#9 combined with anti-CD47 treatment in 4T1 tumor-bearing mice after IV administration of ATV 2.5 mg/kg + DTX 10 mg/kg (MTD) once every 3 weeks, oral administration of ATV 2.5 mg/kg + DTX 10 mg/kg (ATV/DTX [oral]) once daily, oral

administration of MCT-NE#9 based on ATV 2.5 mg/kg (ATV-NE#9 [oral]) once daily, oral administration of MCT-NE#9 based on DTX 10 mg/kg (DTX-NE#9 [oral]) once daily, oral administration of MCT-NE#9 based on ATV 2.5 mg/kg + DTX 10 mg/kg (MCT-NE#9 [oral]) once daily, IP administration of anti-CD47 (10 mg/kg) (anti-CD47 [IP]) at 3-day intervals, and combined therapy consisted of daily oral dosing of MCT-NE#9 (ATV 2.5 mg/kg + DTX 10 mg/kg) together with IP anti-CD47 antibody (10 mg/kg) administered at 3-day intervals (MCT-NE#9 [oral] + anti-CD47 [IP]) for a total of 3 weeks. (**A**) Control, (**B**) MTD, (**C**) ATV/DTX (Oral), (**D**) ATV-NE#9 (Oral), (**E**) DTX-NE#9, (Oral) (**F**) MCT-NE#9 (Oral), (**G**) Anti-CD47 (IP), and (**H**) MCT-NE#9 (Oral) + Anti-CD47 (IP).

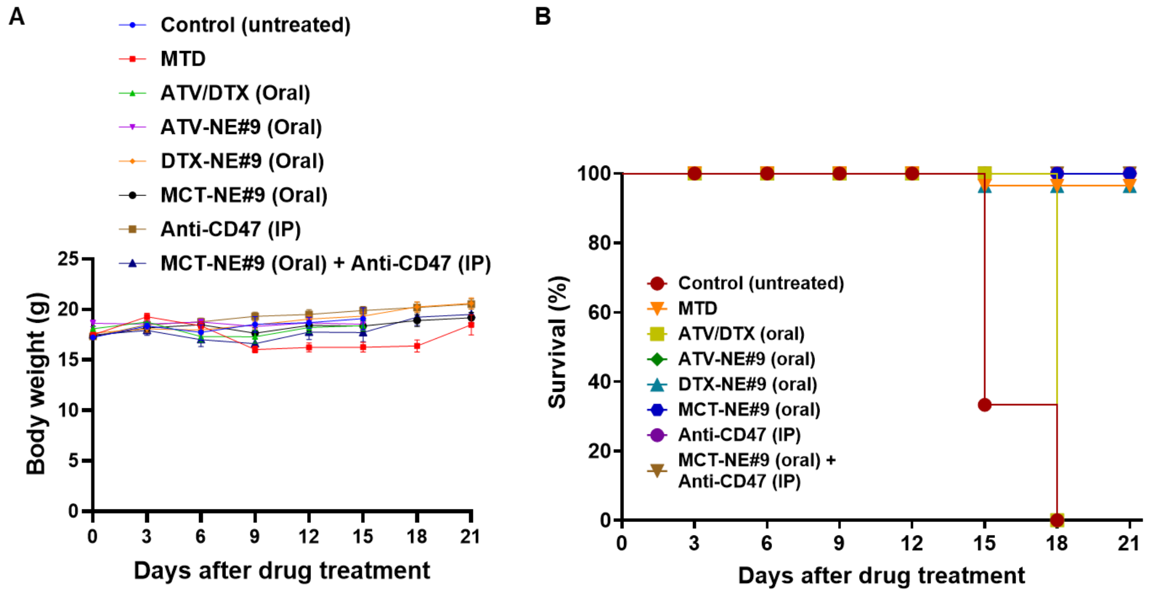

Figure S14. Time-course of (**A**) body-weight changes and (**B**) survival rates in tumor-bearing mice during treatment. Each measurement is shown as the mean ± SD (n = 10 per group).

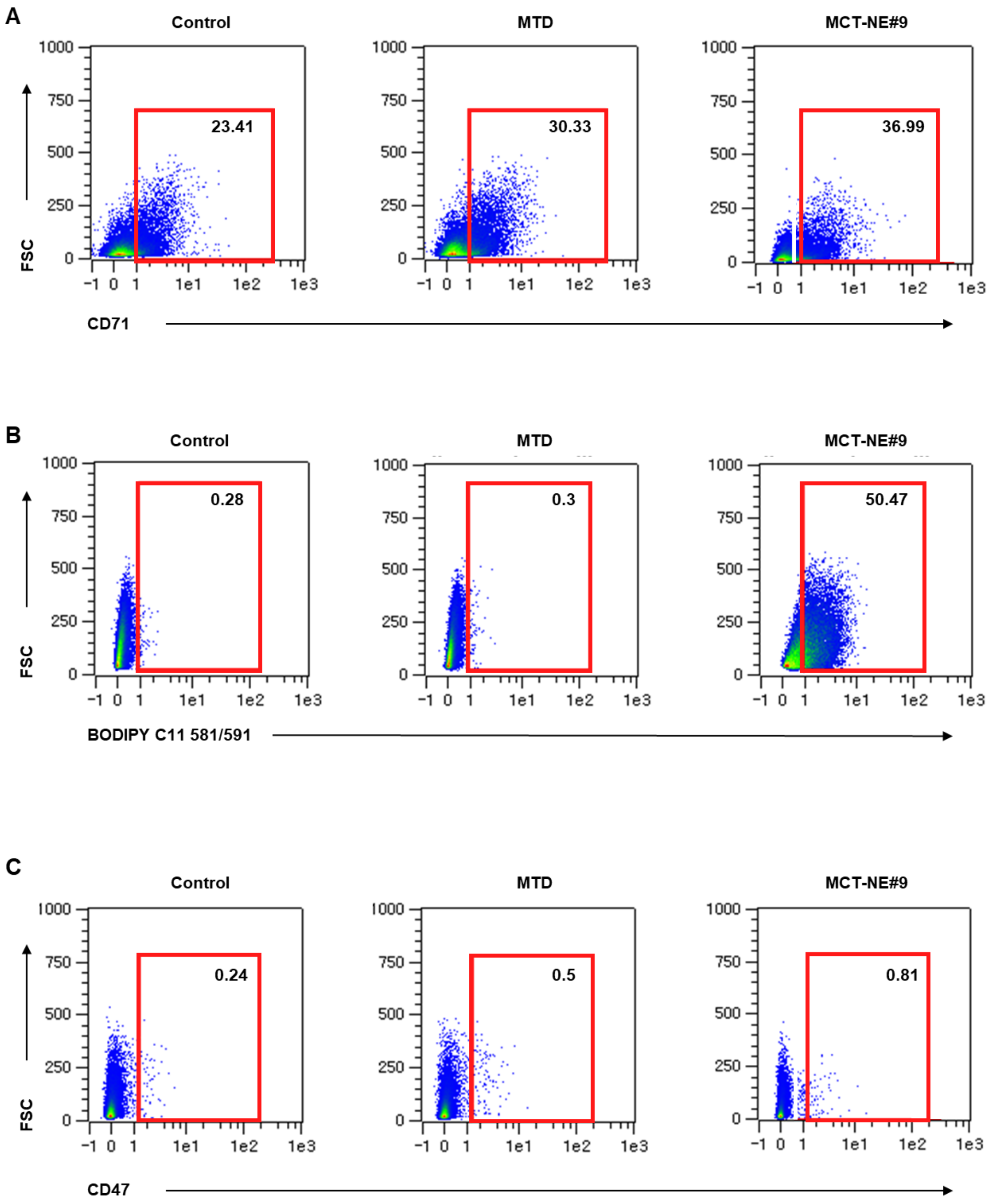

Figure S15. In vivo CD47, CD71, and LPO analysis in tumors. Flow cytometry analysis of cancer cells isolated from tumor tissues after treatment with control, MTD, and MCT-NE#9. (**A**) Quantification of CD47 expression in cancer cells. (**B**) Quantification of CD71 expression in cancer cells. (**C**) Quantification of LPO in cancer cells using BODIPY C11 581/591 staining. The numbers in each plot indicate the percentages of

positive cells.

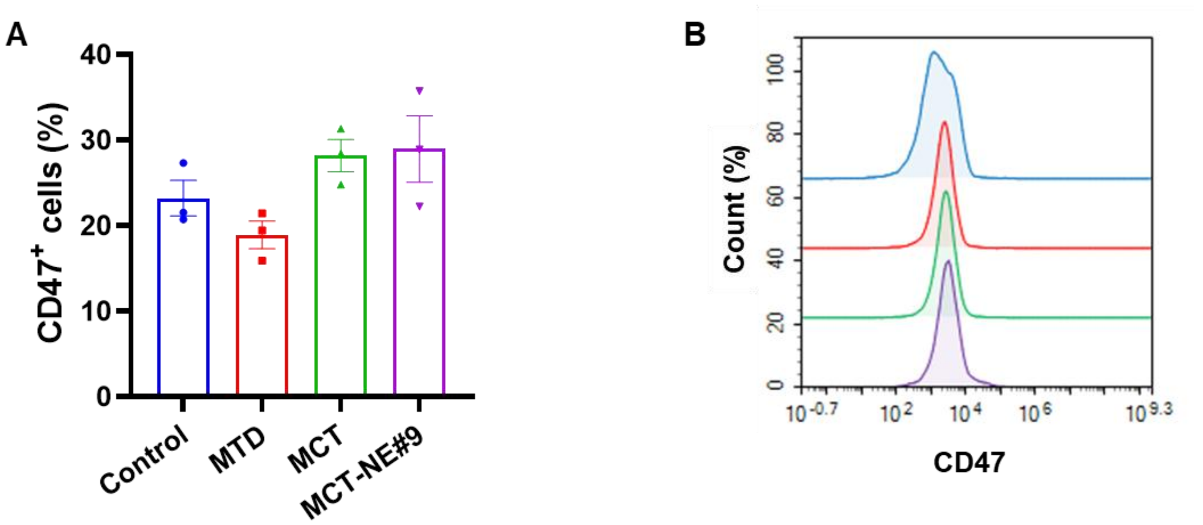

Figure S16. In vitro analysis of CD47 expression in 4T1 cells after treatment. (**A**) Quantification of $CD47^+$ cells (%) among 4T1 cells treated with control, MTD, MCT, and MCT-NE#9. (**B**) Representative flow cytometry histogram showing CD47 expression levels in 4T1 cells across different treatment groups.

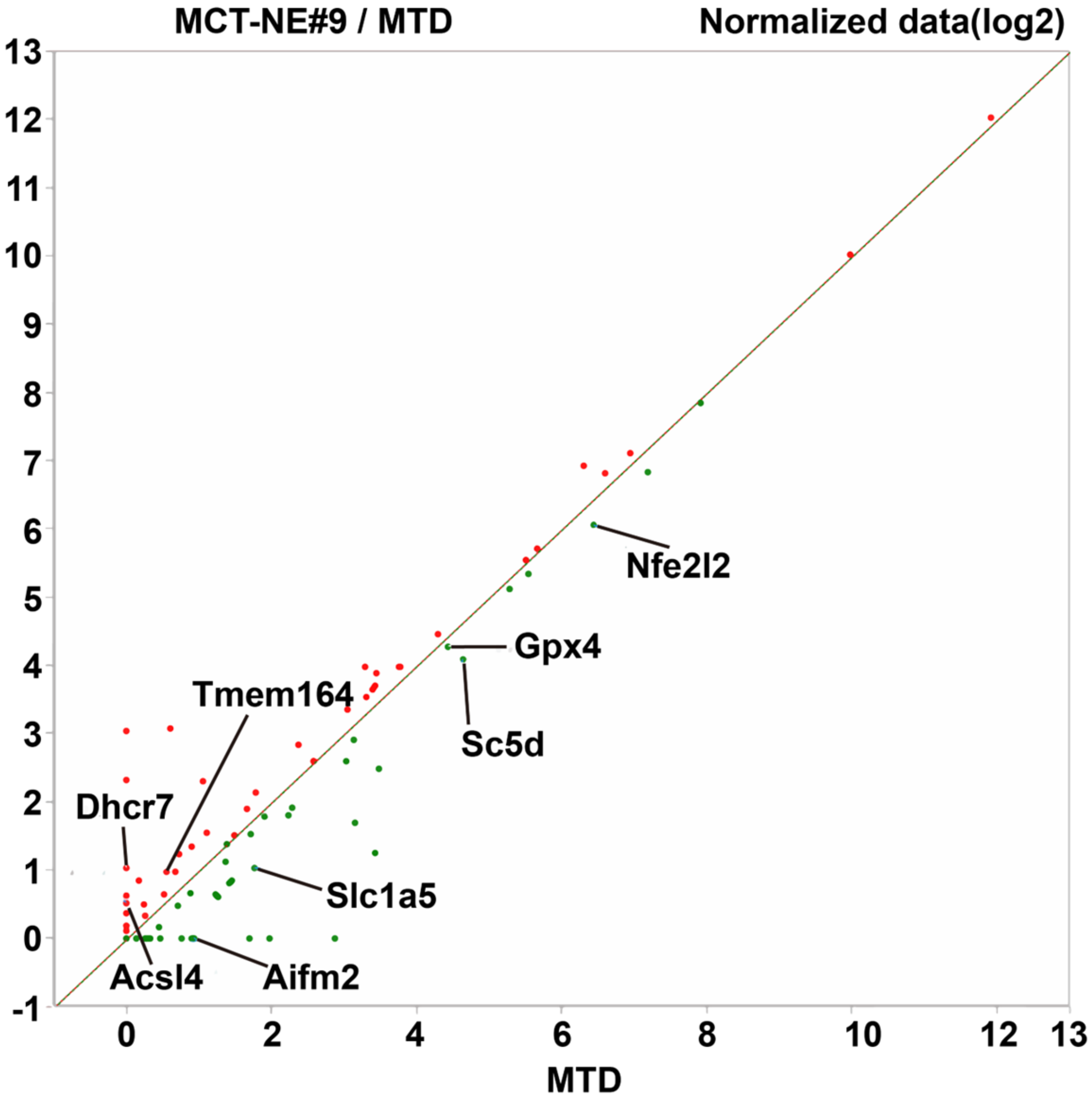

Figure S17. Ferroptosis-related gene expression analysis in tumors treated with MCT-NE#9 compared with MTD. Scatter plot comparing mRNA sequencing data (log2 normalized) between MCT-NE#9 and MTD groups. Key ferroptosis markers such as *Nfe2l2*, *GPX4*, *SLC1A5*, *Aifm2*, and *Dhcr7* are highlighted (n = 3).

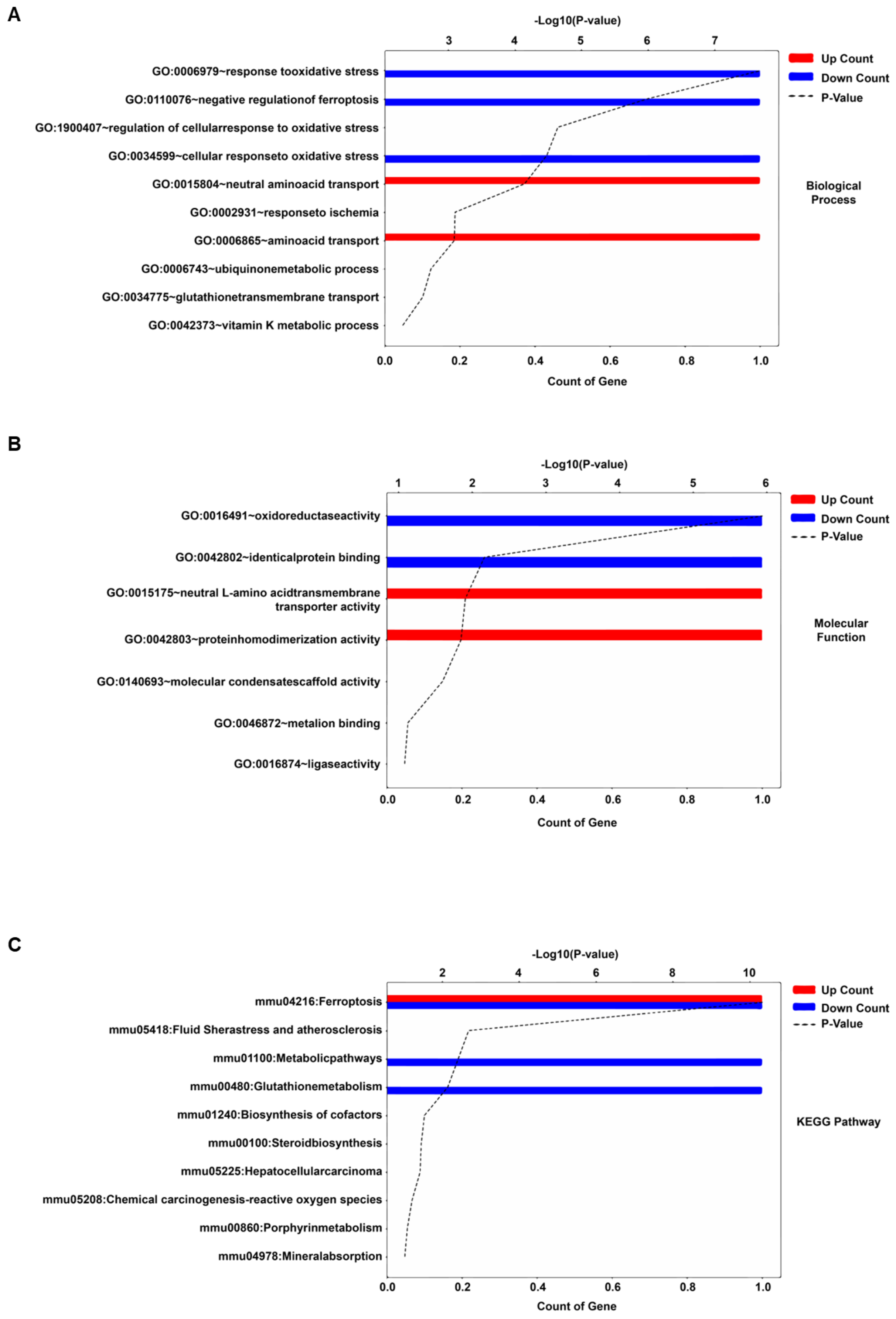

Figure S18. Gene ontology (GO) and Kyoto Encyclopedia of Genes and Genomes

(KEGG) pathway enrichment analysis of differentially expressed genes in the MCT-NE#9-treated group. (**A**) Biological process analysis of upregulated and downregulated genes. (**B**) Molecular function analysis highlighting enriched functional categories. (**C**) KEGG pathway showing significantly enriched pathways related to ferroptosis metabolism and immune regulation. Red bars indicate upregulated pathways, while blue bars indicate downregulated pathways. The dotted line represents the −log10 (p-value) (n = 3).

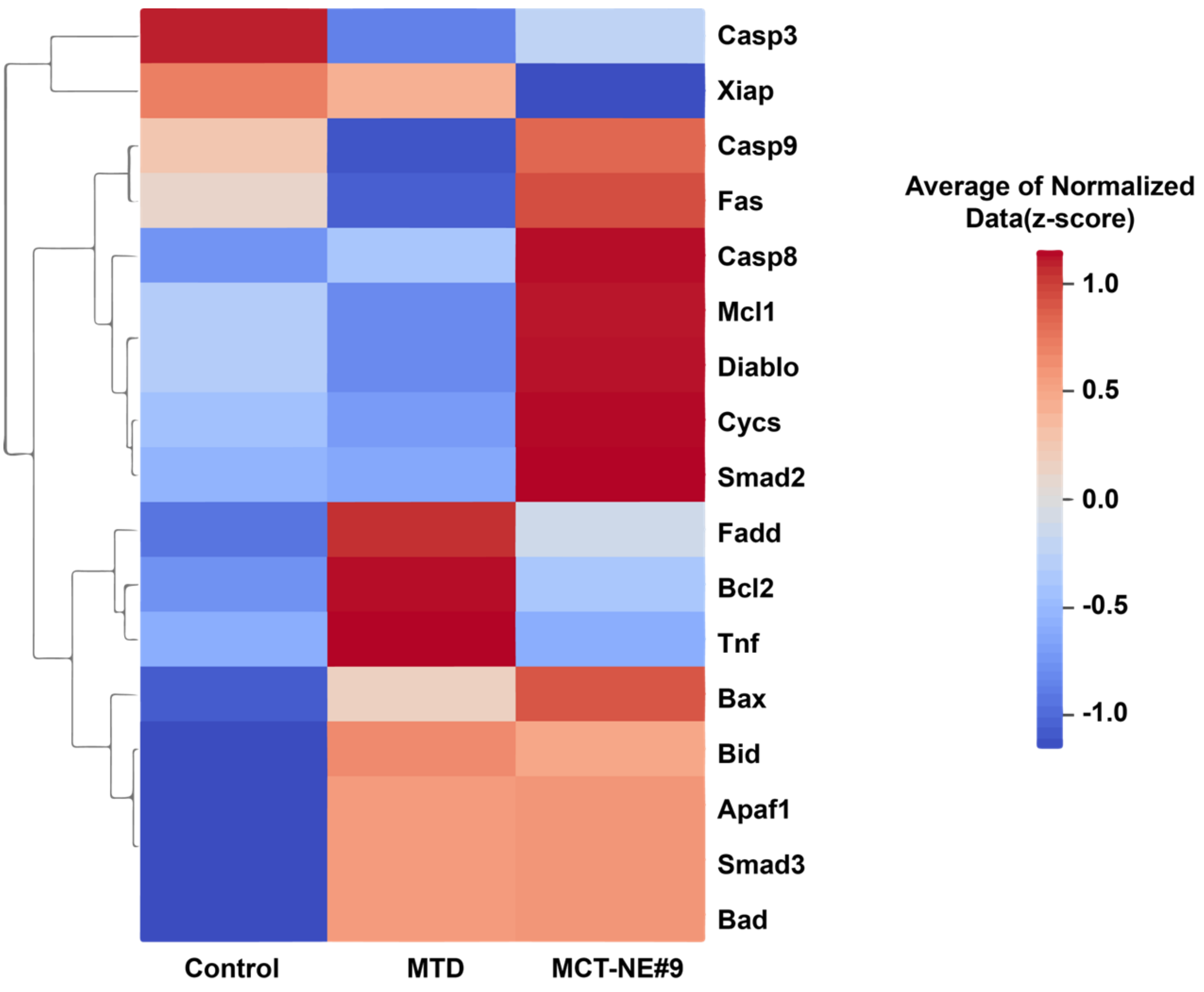

Figure S19. Heatmap analysis of apoptosis-related gene expression in tumor tissues. Heatmap showing differential expression of apoptosis-related genes across control, MTD, and MCT-NE#9 groups. Pro-apoptotic and anti-apoptotic genes were analyzed. Red indicates upregulation and blue indicates downregulation of key immune markers (n = 3).

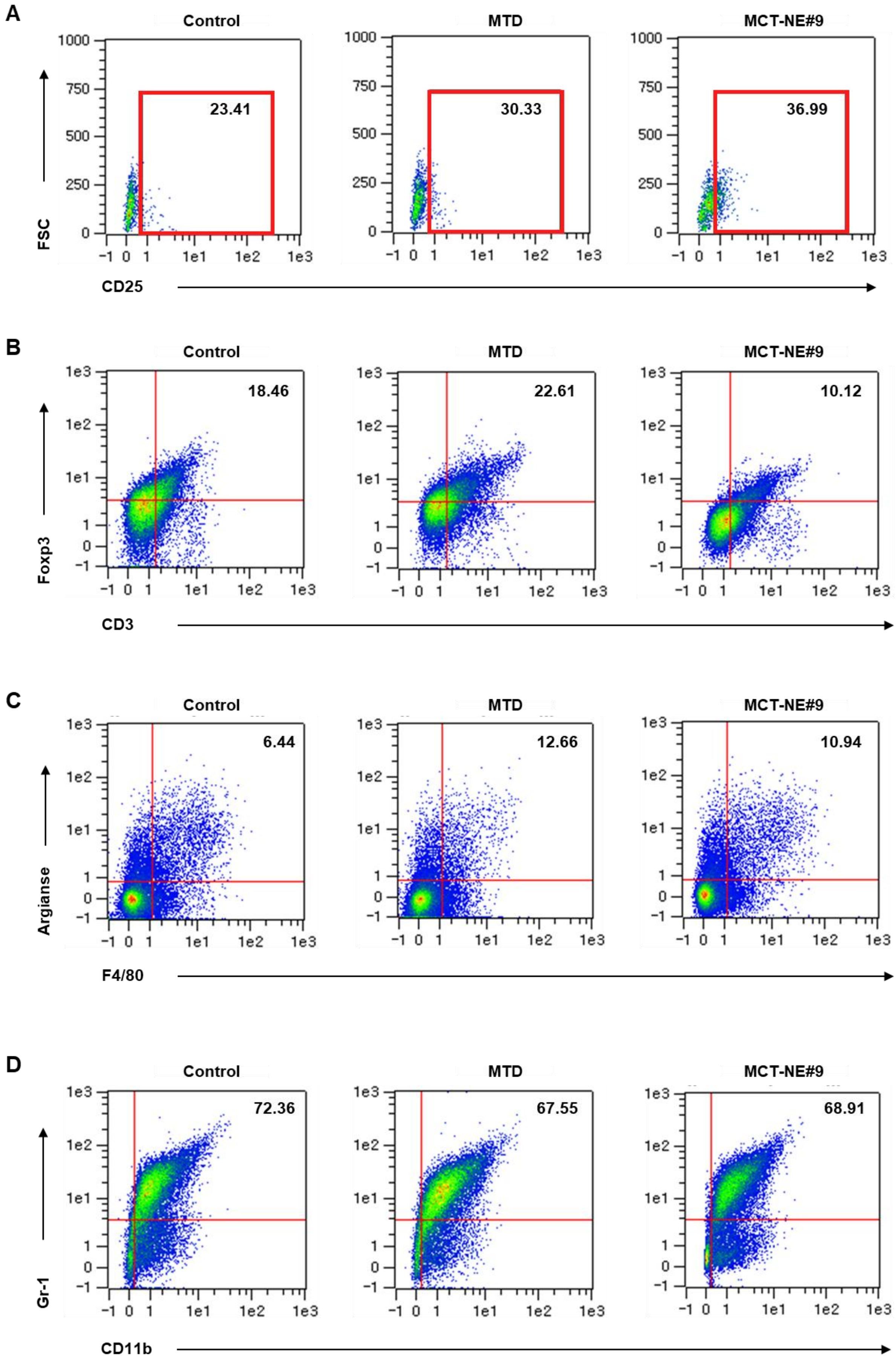
A
Control
MTD
MCT-NE#9
23.41
30.33
36.99
FSC
CD25
B
18.46
22.61
10.12
Foxp3
CD3
C
6.44
12.66
10.94
Argianse
F4/80
D
72.36
67.55
68.91
Gr-1
CD11b

Figure S20. In vivo analysis of T-cell activation, T_{regs}, M2 macrophages, and MDSCs in tumor tissue. Flow cytometry analysis of immune cell populations in tumor tissues after treatment with control, MTD, and MCT-NE#9. (**A**) Quantification of activated T cells ($CD3^+$, $CD25^+$) in tumor tissue. (**B**) Quantification of T_{regs} ($CD3^+$, $Foxp3^+$) in tumor tissue. (**C**) Quantification of M2 macrophages ($F4/80^+$, $Arginase^+$) in tumor tissue. (**D**) Quantification of MDSCs ($CD11b^+$, $Gr\text{-}1^+$) in tumor tissue. The numbers in each plot indicate the percentages of positive cells.

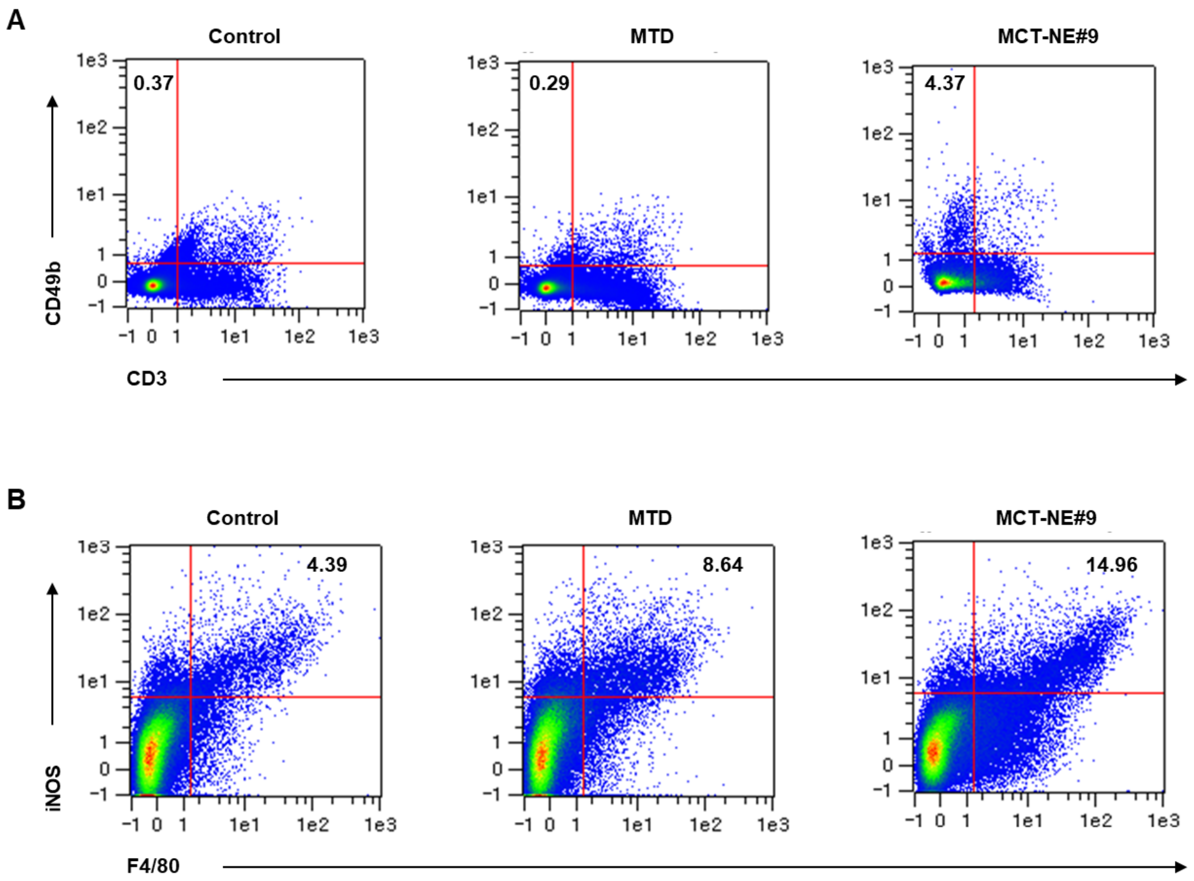

Figure S21. In vivo analysis of NK cells and M1 macrophages in tumor tissue. Flow cytometry analysis of immune cell populations in tumor tissues after treatment with control, MTD, and MCT-NE#9. (**A**) Quantification of NK cells ($CD3^-$, $CD49b^+$) in tumor tissue. (**B**) Quantification of M1 macrophages ($F4/80^+$, $iNOS^+$). The numbers in each plot indicate the percentages of positive cells.

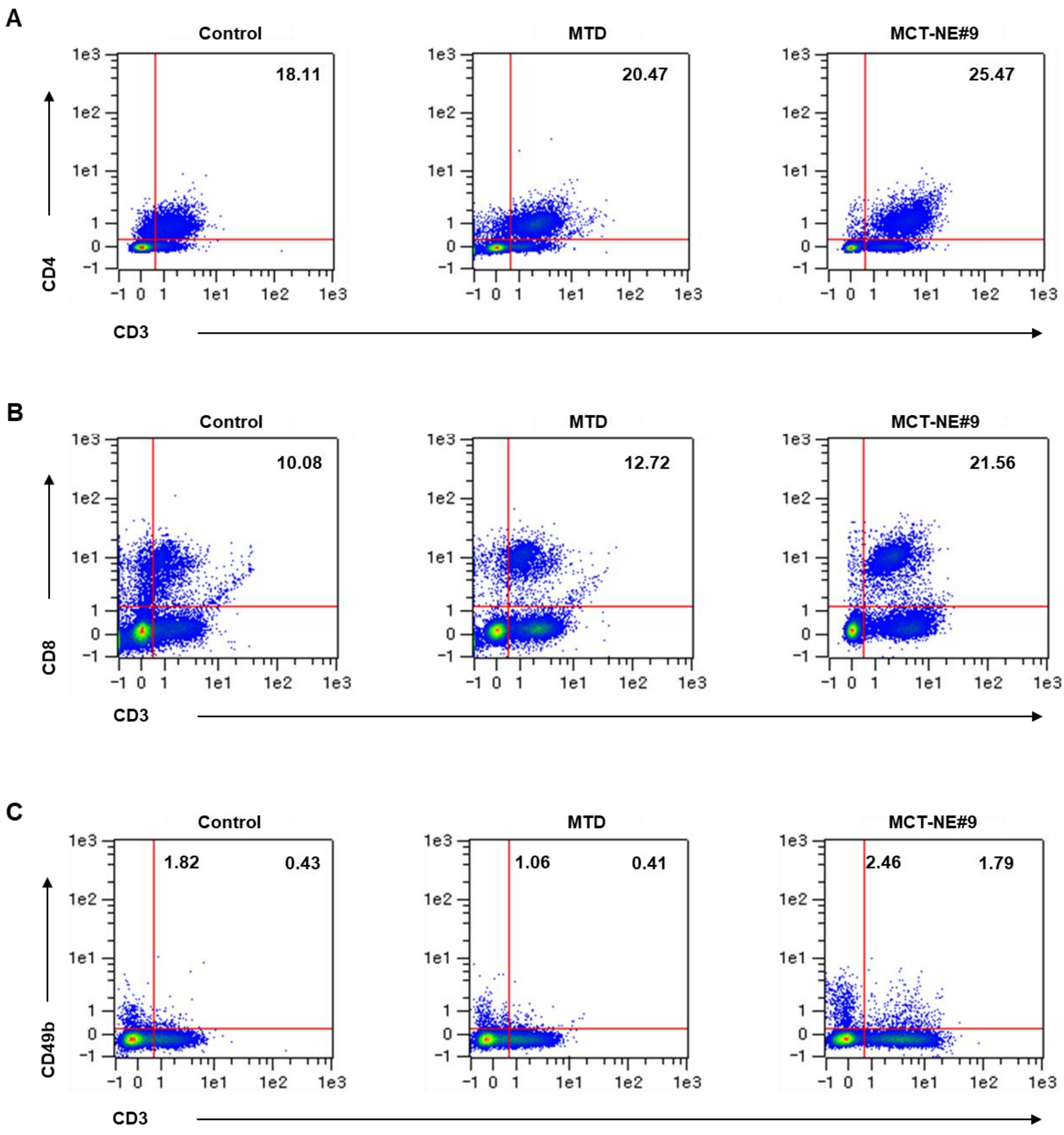

Figure S22. In vivo profiling of T cell and NK cell subsets in TDLNs. TDLN immune populations were evaluated by flow cytometry following control, MTD, or MCT-NE#9 treatment. (**A**) Quantification of CD4 T cells ($CD3^+$, $CD4^+$) in TDLNs. (**B**) Quantification of CD8 T cells ($CD3^+$, $CD8^+$) in TDLNs. (**C**) Quantification of NK cells ($CD3^-$, $CD49b^+$) and NKT cells ($CD3^+$, $CD49b^+$) in TDLNs. The numbers in each plot indicate the percentages of positive cells.

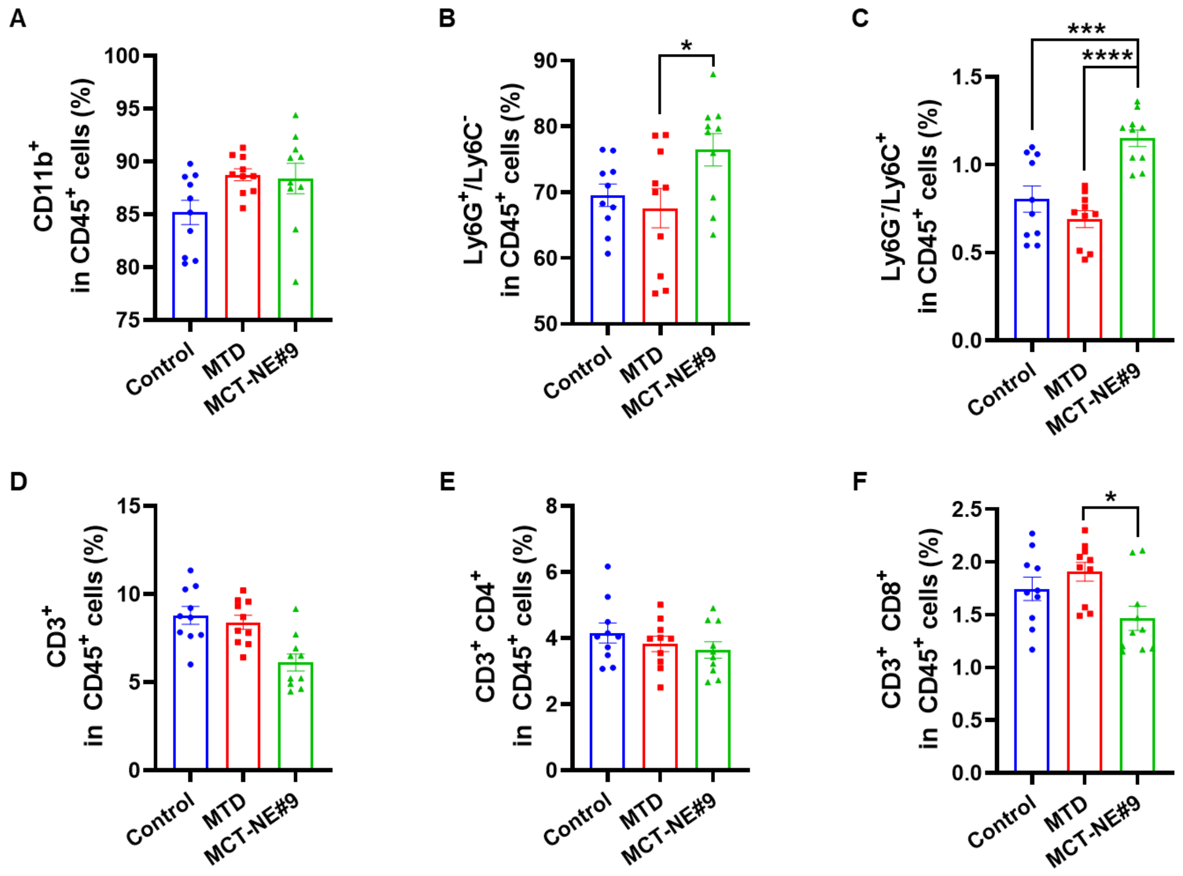

Figure S23. Analysis of immune cell populations in blood. Flow cytometry analysis of immune cell subsets in blood samples from control, MTD, and MCT-NE#9 groups (n = 10). (**A**) $CD11b^+$ myeloid cells, (**B, C**) granulocytes ($Ly6G^+$, $Ly6C^-$) and monocytes ($Ly6G^-$, $Ly6G^+$), (**D**) T cells ($CD3^+$), and (**E, F**) CD4 ($CD3^+$, $CD4^+$) and CD8 T cell ($CD3^+$, $CD8^+$) subsets. The results indicate changes in myeloid and lymphoid immune cell populations following treatment. Data are presented as mean ± SEM (n = 3). * $p < 0.05$, *** $p < 0.001$, **** $p < 0.0001$.

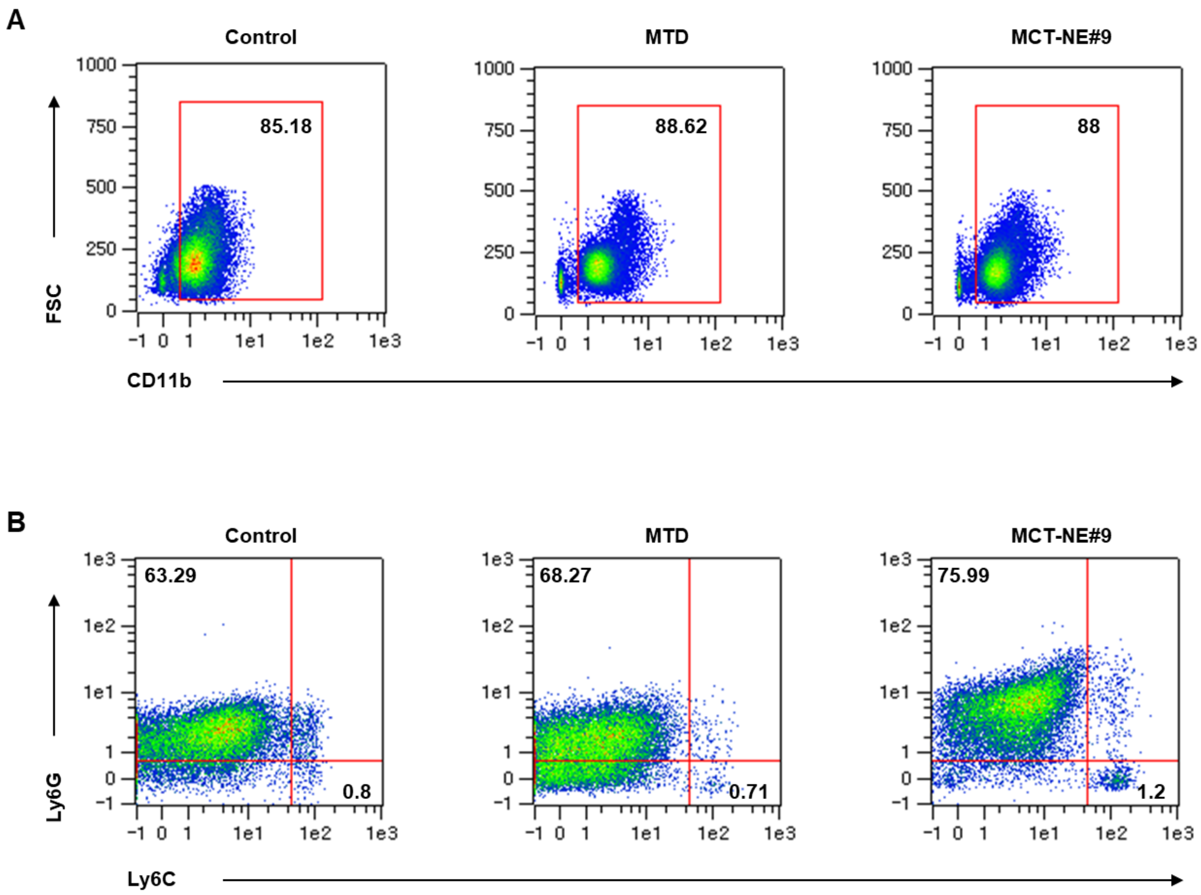

Figure S24. In vivo profiling of blood myeloid populations by flow cytometry after administration of control, MTD, or MCT-NE#9. (**A**) Quantification of $CD11b^{+}$ myeloid cells in blood. (**B**) Quantification of granulocytes ($Ly6C^{-}$, $Ly6G^{+}$) and monocytes ($Ly6C^{+}$, $Ly6G^{-}$) in blood. The numbers in each plot indicate the percentages of positive cells.

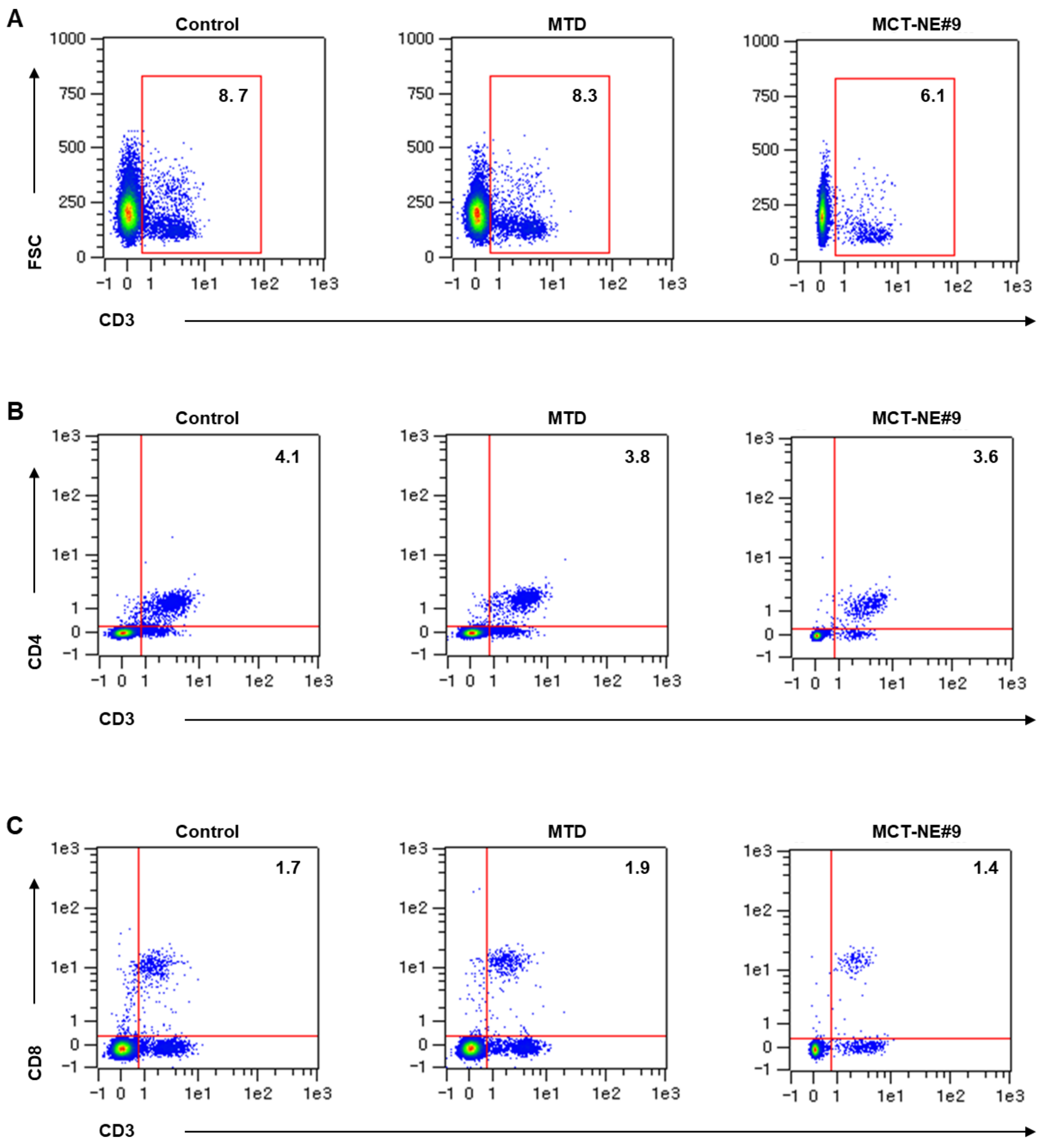

Figure S25. In vivo analysis of T cells in blood. Flow cytometry analysis of T-cell populations in blood after treatment with control, MTD, and MCT-NE#9. (**A**) Quantification of T cells in blood ($CD3^+$). (**B**) Quantification of CD4 T cells ($CD3^+$, $CD4^+$) in blood. (**C**) Quantification of CD8 T cells ($CD3^+$, $CD8^+$) in blood. The numbers in each plot indicate the percentages of positive cells.
